# Comparison of three data mining models for prediction of advanced schistosomiasis prognosis in the Hubei province

**DOI:** 10.1371/journal.pntd.0006262

**Published:** 2018-02-15

**Authors:** Guo Li, Xiaorong Zhou, Jianbing Liu, Yuanqi Chen, Hengtao Zhang, Yanyan Chen, Jianhua Liu, Hongbo Jiang, Junjing Yang, Shaofa Nie

**Affiliations:** 1 Department of Epidemiology and Health Statistics, School of Public Health, Tongji Medical College, Huazhong University of Science and Technology, Wuhan, Hubei, China; 2 Hubei Provincial Center for Disease Control and Prevention, Wuhan, Hubei, China; 3 Department of Mathematics, Wuhan University, Wuhan, Hubei, China; 4 Yichang Center for Disease Control and Prevention, Yichang, Hubei, China; 5 Department of Epidemiology and Biostatistics, School of Public Health, Guangdong Pharmaceutical University, Guangzhou, China; RTI International, UNITED STATES

## Abstract

**Background:**

In order to better assist medical professionals, this study aimed to develop and compare the performance of three models—a multivariate logistic regression (LR) model, an artificial neural network (ANN) model, and a decision tree (DT) model—to predict the prognosis of patients with advanced schistosomiasis residing in the Hubei province.

**Methodology/Principal findings:**

Schistosomiasis surveillance data were collected from a previous study based on a Hubei population sample including 4136 advanced schistosomiasis cases. The predictive models use LR, ANN, and DT methods. From each of the three groups, 70% of the cases (2896 cases) were used as training data for the predictive models. The remaining 30% of the cases (1240 cases) were used as validation groups for performance comparisons between the three models. Prediction performance was evaluated using area under the receiver operating characteristic curve (AUC), sensitivity, specificity, and accuracy. Univariate analysis indicated that 16 risk factors were significantly associated with a patient’s outcome of prognosis. In the training group, the mean AUC was 0.8276 for LR, 0.9267 for ANN, and 0.8229 for DT. In the validation group, the mean AUC was 0.8349 for LR, 0.8318 for ANN, and 0.8148 for DT. The three models yielded similar results in terms of accuracy, sensitivity, and specificity.

**Conclusions/Significance:**

Predictive models for advanced schistosomiasis prognosis, respectively using LR, ANN and DT models were proved to be effective approaches based on our dataset. The ANN model outperformed the LR and DT models in terms of AUC.

## Author summary

Worldwide, approximately 240 million individuals are infected with schistosomiasis, a parasitic neglected tropical disease that continues to be a significant cause of morbidity and mortality, especially in China. Effective tools that can accurately predict the prognosis of patients with advanced schistosomiasis would aid in the treatment and management of the disease. To this end, we constructed and compared the performance of three predictive models—an artificial neural network (ANN) model, a logistic regression (LR) model and a decision tree (DT) model—in their ability to predict the prognosis of patients with advanced schistosomiasis. We found that while all three models proved effective, the ANN model outperformed the LR and DT models in terms of AUC and sensitivity. Yet, to achieve the highest level of prediction accuracy and to better assist medical professionals, we recommend comparing the performance of the three predictive models to select the optimal one, which will be better than select a model at random. The findings of this study not only provide valuable information on the construction of effective predictive models for the prognosis of advanced schistosomiasis, but also offer new methodology for clinically determining patient diagnosis and prognosis.

## Introduction

Approximately 240 million individuals are infected worldwide by schistosomiasis, with an estimated 3.31 million disability-adjusted life years lost as a result of the disease [1–4].Further, one meta-analysis and several scientific reports have suggested that global burden caused by schistosomiasis may be several times higher[3]. This concern mainly comes from the following reasons. The first reason is that the low sensitivity schistosomiasis diagnostic methods and insufficient investment of health resources may result in underdiagnosis of schistosomiasis in epidemic areas. The second reason is that the value of disability weight (DW) of schistosomiasis might be set too low (0.005–0.006) in the calculation of DALY value, which is similar to those for disorders such as moderate discolouration of the face (facial vitiligo)[5]. The third reason is that whether infected with schistosomiasis was set as the only healthy outcome in the estimation of DALY value rather than considering disparity in different clinical stages of schistosomiasis (acute, chronic, advanced). Fourth, the disparity in different schistosome germline was also not taken into account for the pathological process varies greatly among Schistosoma mansoni, Schistosoma haematobium and Schistosoma japonicum. Nevertheless, Schistosomiasis was still regarded as one of the most important neglected tropical diseases worldwide.

In China, schistosomiasis has been endemic in 12 provinces and municipalities [6]. Currently, the prevailing regions endemic for schistosomiasis are located in the lake and marshland regions, such as Hunan, Hubei, Jiangxi, Jiangsu, and Anhui, and in the hilly and mountainous regions, such as in Yunnan and Sichuan. However, other regions, such as Fujian, Guangdong, Shanghai, Zhejiang, and Guangxi have successfully fulfilled the criteria for interrupting schistosomiasis transmission since 1985[7]. Hubei province is one of the five lake and marshland schistosomiasis endemic regions which located in the middle and lower regions of the Yangtze River [8]. In addition, Hubei has the largest area of the freshwater snail *Oncomelania hupensis*, which is the only intermediate host of *Schistosoma japonicum*. Moreover, Hubei has the highest rates of schistosomiasis transmission in China [9].

By 2015, Hubei had 9098 (29.50% of China’s total cases) documented cases of advanced schistosomiasis, ranking Hubei first in all schistosomiasis endemic provinces in China [10]. Advanced, or late-stage schistosomiasis japonica can be regarded as an extreme form of chronic schistosomiasis, which is more serious than the advanced hepatosplenic disease of Schistosoma mansoni infection found in Africa and the Americas [11]. According to ‘Diagnostic Criteria for Schistosomiasis’ (WS261-2006), one of health industry standards in People's Republic of China provided by National Ministry of Health, the advanced schistosomiasis case is defined as a patient with schistosomiasis who develops portal hypertensive syndromes of liver fibrosis, severe growth disorders or significant colon granulomatous hyperplasia. Due to repeated or mass infection of schistosome cercariae, without thorough and timely treatment, patients can evolve into advanced schistosomiasis usually after 2 to 10 years of pathological development process. Clinical symptoms of advanced schistosomiasis include ascites, splenomegaly, portal hypertension, gastro-esophageal variceal bleeding, granulomatous lesions of the large intestine, and serious growth retardation [12, 13]. Advanced schistosomiasis japonica is much more common in highly endemic areas, because repeated, heavy exposure to cercariae means that early-stage chronic cases may not be effectively treated in routine control programs. The eggs of *S*. *japonicum* retained in the intestine and liver tissue stimulate a granulomatous response, leading to continuous fibrosis of the periportal tissue and developing a pipestem fibrosis. Although down-modulation of the granulomatous response, which could prevent further chronic morbidity after 2–5 years or more, parasite-induced periportal fibrosis may progress to cause obstruction of the portal vessels and damage to the liver parenchyma, leading to development of advanced schistosomiasis. Mortality eventually results from bleeding of the upper gastrointestinal tract, spontaneous bacterial peritonitis, and hepatic failure, among other factors. Based on its major symptoms, advanced schistosomiasis japonica in China represents a widespread, serious health burden, and has been classified into four clinical sub-types, namely ascites, megalosplenia, colonic tumorous proliferation, and dwarfism [14, 15].

Predictive models used in disease prognosis studies can answer the following questions such as the seriousness of the patient’s condition and whether can be cured. Also it can be used to guide clinical treatment and help to select the right medical decision-making. Therefore, the predictive model is of great significance. Specifically, the predictive model can be used to understand the trends and consequences of a disease and help clinicians make treatment decisions and determine the urgency of treatment. The model can be applied to study the various influencing factors that affect the prognosis of the disease and assess the effectiveness of a treatment.

Logistic regression (LR) model is a probabilistic non-linear regression model. As a popular multivariate analysis method, it is widely used to study the relationship between dichotomous observations and some influencing factors. In epidemiology, LR model is always used to explore the risk factors of a disease, predict the probability of a disease occurring based on risk factors and so on. For example, to explore the risk factors for gastric cancer (GC), you can choose two groups of people, a GC group and a non-GC group with different signs and lifestyles. The dependent variable here is gastric cancer ("yes" or "no"), while independent variables can covers a lot, such as age, gender, eating habits, Helicobacter pylori infection. The arguments in the model can be either continuous or categorized. By logistic regression analysis, we can get a general understanding of which factors are risk factors for GC.

ANN model is a mathematical model that simulates the structure of the human brain and the way of information transmission. It consists of a set of interconnected “neurons” linked with weighted connections. The model was constructed by an input layer, a hidden layer and an output layer. The input layer contains neurons that receive input data available for analysis (e.g. various demographical, clinical or laboratory data), and output layer contains neurons that export different values.ANN can learn through examples and associate each input with the corresponding output by modifying the weight of the connections between neurons. The output value is compared with the expected output. If there is a discrepancy between these two values, an error signal is generated and then a back propagation (BP) method is applied to alter the weight of the connections between neurons to decrease the overall error of the network. As learning proceeds, the error between the ANN output and the expected output decreases until a minimum is reached. The process was called convergence of the network. After these two training processes, the ANN can generate outputs (prognosis) from new input data based on the knowledge accumulated during training, which is regarded as inference process. Thus, after training, the ANN can make predictions on data sets never seen before or identify patterns.

There are some similar studies. A study demonstrated that the ANN model is a more powerful tool in determining the significant prognostic variables for gastric cancer (GC) patients, compared to the Cox proportional hazard regression (CPH) model [16]. In another study, the ANN model was shown to be more accurate in predicting 3-month mortality of acute-on-chronic hepatitis B liver failure (ACHBLF) than Model for end-stage liver disease (MELD) based scoring systems [17]. In addition to these examples, a trained ANN performs at least as well as physicians in assessments of visual fields for the diagnosis of glaucoma in a ophthalmology research [18].

The decision tree (DT) is a machine learning model, composed of decision rules based on optimal feature cutoff values that recursively split independent variables into different groups to predict an outcome in a hierarchical manner. The principle of DT is similar to that of variance discomposition in ANOVA. The basic purpose is to divide the research population into several relatively homogeneous subgroups through some attribute values. The values of internal variables in each subgroup are highly consistent, and the corresponding variations (impurities) fall in different subgroups as far as possible. All DT model algorithms follow this principle, which is different from ANOVA by definition of variation (impurity), such as P values, variance in ANOVA and information entropy, G1NI coefficients, deviance in DT.

Some examples are also provided. A simple, clinically relevant DT model was developed and validated to reliably discriminate patients at high and low risk of death using routinely available variables from the time of diagnosis in unselected populations of patients with malignant pleural mesothelioma (MPM) [19]. Another simple decision tree can provide a quick assessment of the severity of the chronic obstructive pulmonary disease (COPD) by using variables commonly gathered by physicians, as measured by the risk of 5-yr mortality [20]. The DT modeling based on C4.5 algorithm which was applied to predict prostate cancer risk in another study showed different interaction profiles by race [21].

Traditional LR model is the most popular predictive among different classification methods because the effects of each factors in LR model could be quantitatively explained and an approximately estimate of the relative risk (OR) could be derived easily. However, whether the data could fit the model requires that the data satisfy a given condition and the collinearity and interaction between the variables cannot be solved. ANN model possesses strong ability to solve such problems and has no limitation on the distribution of data. It is generally believed that the ANN model is better than LR model for the disease with many pathogenic factors and complicated relationships among these factors. The DT model also generally considers the interaction between the variables, and it shows a clear screening process in the form of a tree. Compared with the OR value of LR model, the DT model is more conducive for clinicians' understanding. Therefore, the aim of this study was to compare the performance of three predictive models (ANN, LR and DT) for the prognosis of advanced schistosomiasis cases, along with a 10-fold cross-validation technique. The performance of the predictive models was evaluated according to the area under the receiver operating characteristic curves (AUC), accuracy, sensitivity, and specificity.

## Methods

### Ethics statement

The study was approved by Research Ethics Committee in Tongji Medical College of Huazhong University of Science and Technology. The methods of the present study were put into effect according to the approved protocols. All participants in this study were adults. Note: Though a child from Xingzi county, Jiangxi Province had ever been reported to diagnosed as advanced schistosomiasis[22], such case is exceedingly rare and never been reported in Hubei. In general, all the advanced schistosomiasis patients are adults.

The participants read the investigation purpose statement and signed informed consents. All data were anonymized and handled confidentially.

### Data collection and variable selection

Schistosomiasis surveillance data was collected from a previously constructed database of advanced schistosomiasis cases in the Hubei province from a study conducted by the Hubei Institute of Schistosomiasis Prevention and Control. The information was obtained by a standard sociodemographic and epidemiological questionnaire for patients in Hubei with advanced schistosomiasis. Participants were recruited from schistosomiasis epidemic areas all over the province, primarily along the Yangtze River regions. The treatment methods of advanced schistosomiasis patients vary with different disease conditions. Liver protection and symptomatic treatment was applied for ascites type patients. Splenectomy was needed to be done in splenomegaly patients if there is hypersplenism symptom existed. The praziquantel (PZD) treatment can be utilized after six months of stable period in which the general situation of the patient is fine (e.g. no ascites or hemorrhage symptoms).

The medical records of the patients with advanced schistosomiasis were reviewed by attending physicians. Criteria of cases inclusion are as follows:

1. Diagnosed as advanced schistosomiasis;
2. A long-term repeated schistosome water contact history or a clear history of schistosomiasis treatment was existed.
3. The schistosome eggs or miracidia was detected by fecal examination, or schistosome eggs were detected by rectal biopsy, or serum immunological tests were positive.
4. Patients with abdominal distension, fatigue, loss of appetite and other symptoms;
5. The informed consent of the patients were obtained.

To avoid the confounding effect of other diseases on the prediction of advanced schistosomiasis prognosis, the patients with following diseases were excluded from the study.

1. Primary liver cancer or other intrahepatic space occupying lesions;
2. Obstructive jaundice or hemolytic jaundice;
3. Combined with cardiovascular disease, serious primary diseases of kidney and hematopoietic and other systems, as well as mental diseases.

A total of 4136 cases were included in the study which consisted of 2674 men and 1462 women and were divided into two groups: favorable prognosis and poor prognosis. Favorable prognosis referred to cases of recovery and improved disease outcomes while poor prognosis referred to cases of deterioration and death. The presence of the event (dead or deterioration) was coded as 1 and the absence of the event (recovery or improved) was coded as 0. The death of advanced schistosomiasis patients was mainly due to schistosomiasis and schistosomiasis-induced complications, such as upper gastrointestinal hemorrhage, hepatorenal syndrome (HRS), hepatic coma and liver cancer. Therefore, the death outcome that appears in this article refers to all-cause death. The deterioration outcome means that the primary symptoms persist (e.g. no ascites regression sign) or patients in splenomegaly type have no surgical indications.

Data collection included demographical data, hospitalization costs, clinical features, surgical procedures, and outcomes. This study was entirely retrospective which was utilizing records from the hospitals specializing in schitosomiasis of various epidemic counties, Hubei province.

In the first step, the continuous explanatory variables were transformed into categorized variables to decrease the effect of extreme values and enhance the computational efficiency of the ANN. The cutoff points of these variables were set as 0.5. The variables included occupation, annual income, body mass index (BMI) and so on. The sociodemographic and epidemiological characteristics of the 4136 advanced schistosomiasis cases are presented in **Table 1**. The criterion used for the histopathologic diagnosis of advanced schistosomiasis was the national standardized diagnostic criteria for schistosomiasis (WS261-2006). In the second step, a univariate Cox proportional hazard model was used to improve the computational efficiency and prediction performance of the ANN model by testing the potential relationships between independent variables. Variables with statistically significant differences (log-rank test, *P<*0.05) were reserved to build the ANN model (**Table 1**). In total, 16 variables were selected to build the ANN model.

**Table 1:** Comparison of essential features between training and validation groups.

Variables	Definition	Training(N = 2896)		Validation(N = 1240)		*P*
		N	%	N	%	
Occupation	Farmer	2802	96.8	1192	96.1	0.312
	Other	94	3.2	48	3.9	
Annual Income	<10000 Yuan	1871	64.6	778	62.7	0.252
	≥10000 Yuan	1025	35.4	462	37.3	
BMI	18.5–23.9	1944	67.1	838	67.6	0.858
	<18.5	290	10.0	127	10.2	
	24≤BMI<28	383	13.2	152	12.6	
	≥28	279	9.7	123	10.0	
Development^1^	Normal	2829	97.7	1208	97.4	0.367
	Poor	66	2.3	30	2.4	
	Supernormal	1	0.0	2	0.2	
Nourishment	Well	380	13.1	173	14.0	0.632
	Medium	2356	81.4	989	79.7	
	Bad	157	5.4	77	6.2	
	Cachexia	0	0.1	1	0.1	
Diagnostic Evidence 1	Blood test	1369	47.2	567	45.7	0.319
	Stool test	610	21.0	288	23.2	
	Blood+Stool	858	29.6	354	28.6	
	Proctoscopy	59	0.2	31	2.5	
Diagnostic Evidence 2	Ascites type^2^	2177	75.2	899	72.5	0.071
	Non-ascites type	719	24.8	341	27.5	
Prior treatment	No	183	6.3	68	5.5	0.303
	Yes	2713	93.7	1172	94.5	
History of splenectomy	No	1720	59.4	741	59.8	0.826
	Yes	1176	40.6	499	40.2	
History of ascites	No	271	9.4	100	8.1	0.182
	Yes	2625	90.6	1140	91.9	
Other disease	No	1538	53.1	668	53.9	0.869
	Cardiovascular	344	11.9	148	11.9	
	Digestive system	621	21.4	260	21.0	
	Neuropsychiatric system	16	0.6	5	0.4	
	Respiratory system	72	2.5	31	2.5	
	Urinary system	49	1.7	14	1.1	
	Other system	256	8.8	114	9.2	
The extent of ascites	Mild to moderate	2881	99.5	1233	99.4	0.850
	Severe	15	0.5	7	0.6	
Clinical classification	Ascites	443	15.3	194	15.6	0.271
	Megalosplenia	2439	84.2	1043	84.1	
	Colonic tumoroid proliferation	2	0.1	2	0.2	
	Dwarfism and others	12	0.4	1	0.1	
Type of treating patients	Approved retreated patients^3^	2682	92.6	1154	93.1	0.563
	Approved relapsed patients^4^	158	5.5	68	5.5	
	Approved new patients^5^	56	1.9	18	1.5	
Means of treatment	Medical treatment	2775	95.8	1200	96.8	0.147
	Surgical treatment	121	4.2	40	3.2	
Cost of treatment	≤5876.9 Yuan	1578	54.5	657	53.0	0.374
	>5876.9 Yuan	1318	45.5	583	47.0	

^1^ Development refers to the growth development status of advanced schistosomiasis patients.

^2^ Ascites type refers to the advanced schistosomiasis patients with clinical manifestations of ascites due to schistosomiasis hepatic fibrosis and impaired liver function.

^3^ Approved retreated patients refer to the patients who was approved to be treated in the next year with the disease was improved after treatment in the registration year.

^4^ Approved relapsed patients refer to the patients who was approved to be needed further treatment for disease reccurence, while he is clinically cured after previous treatment.

^5^ Approved new patients refer to newly discovered advanced schistosomiasis patients.

The approval works mentioned above are completed by the expert panel based on diagnostic criteria for advanced schistosomiasis.

### Training and validation data sets

Patients were randomly assigned to the training group (70% of the total cases) for the development of the ANN, DT, and LR models. The rest of the patients (30% of the total cases) were assigned to the validation groups for the assessment of model performance. Of the 4136 patients with advanced schistosomiasis, 2896 were assigned to the training group and1240 were assigned to the validation group. As listed in Table 1, the effects of the input variables did not significantly differ between the training group and the validation group of all three models (*P>*0.05), indicating the reliability of the data partition.

### Development of three data mining models

The data mining software package MATLAB (Matrix Laboratory, Math Works Company, USA, R2014a software) was used to run ANN and C4.5 DT models.

SPSS 19.0 (IBM Corp, Armonk, NY, USA) was used to establish the LR model.

For all comparisons, differences were tested with two-tailed tests and P values less than 0.05 were considered statistically significant.

### a. ANN model

**Fig 1:**
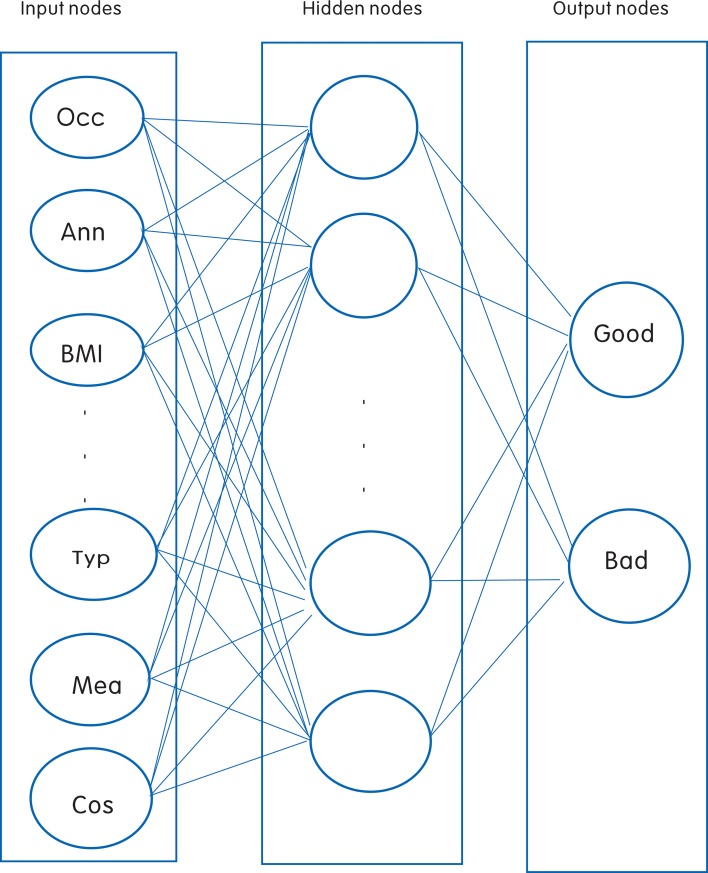
ANN model showing input variables (input nodes), hidden nodes, and connection weights with output nodes for data on patients with advanced schistosomiasis. The ANN model consisted of 16 input nodes, 20 hidden nodes, and two output nodes. Data from a total of 4136 patients with advanced schistosomiasis were used in the ANN analysis. The 16 input nodes were occupation, annual income, BMI, development, nourishment, diagnostic evidence 1, diagnostic evidence 2, prior treatment, history of splenectomy, history of ascites, other disease, the extent of ascites, clinical classification, type of treating patients, means of treatment, and cost of treatment.

An ANN is one of the most widely applied models in the medical domain, such as for the interpretation of imaging techniques, prognosis, diagnosis, or diagnostic tests. ANN differs from other conventional statistical models in that ANN usually has more parameters. This study used an ANN model with a standard feed-forward back propagation (BP) network structure, including an input layer of 16 neurons, a hidden layer of 20 neurons, and an output layer of 2 neurons, to predict the prognosis of patients with advanced schistosomiasis. Sigmoid transfer functions were applied to the hidden and output layers. Gradient descent was used to calculate the synaptic weights. The initial learning rate was defined as 0.07 and the momentum was 0.95. The batch size was defined as 256 and the number of iterations was 200. Ten-fold cross-validation was employed. Fig 1 shows the structure of the ANN model. As there is currently no accepted theory that predetermines the optimal number of hidden layer neurons, the number of hidden layer neurons was determined by repeated trial and error test until the best sensitivity and specificity was achieved.

### b. LR model

For the categorical dependent variables, a LR model was conducted to identify the risk factors of various diseases by using patient demographic characteristics and other disease parameters. The LR model formula calculates the probability of a given disease, y (y = 1 if the selected case suffers from the disease, otherwise, y = 0). If the subject suffers from the disease, the conditional probability is represented as p(y = 1∣X) = p(X), and the formula of the LR model is expressed as log [(p(x) ∣1− p(x)] = β_0_+β_1_x_1_+β_2_x_2_+…+β_k_x_k_],where X = (x_1,_ x_2,…,_ x_k_) denotes the vector of independent variables. An ‘entry’ approach was used to construct the LR model using the 16 variables. The LR model was built using the training dataset and tested using the validation data.

### c. DT model

**Fig 2:**
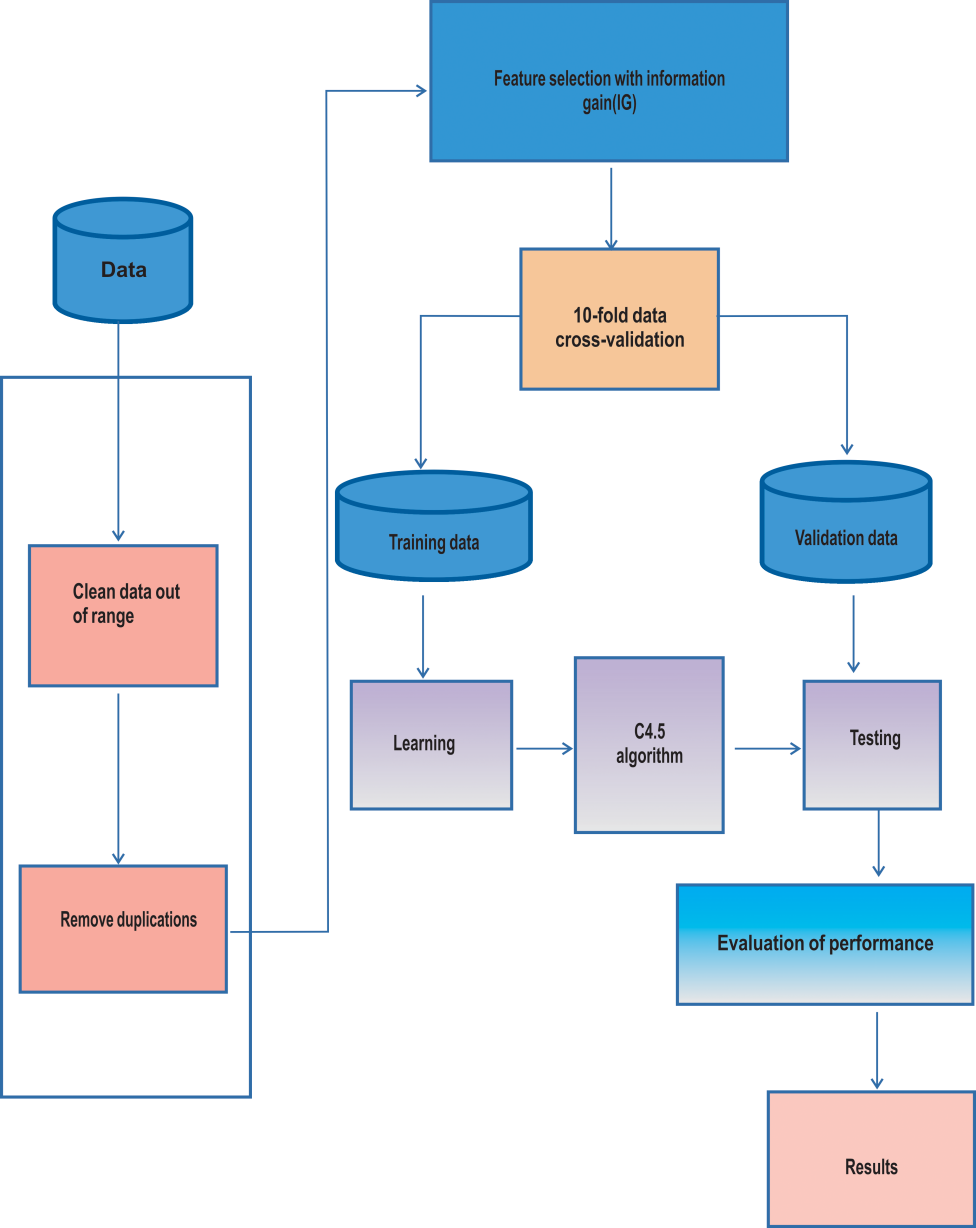
The establishment of the DT model for the prognosis of patients with advanced schistosomiasis (C4.5 algorithm).

The model-based clinical data interpretation system C4.5 algorithm for the prognosis of advanced schistosomiasis is shown in Fig 2.C4.5 was used as the multiclass classification algorithm, which was a development of the DT algorithm ID3. The algorithm contained the same working principle, but calculated information gain differently. In the ID3 algorithm, the learning process is conducted in reference to the gain calculation, which is the same gain calculation in the feature selection process of the information gain, as shown in Eqs (1) and (2). In the C4.5 algorithm, the learning process uses the ID3 normalized gain, as shown in Eqs (3) and (4):

$$Entropy\left(S\right)=\sum_{t}^{c}-p_{i}log_{2}\left(p_{i}\right)$$ (1)

$$Gain\left(S,A\right)=Entropy\left(S\right)-\sum_{v\in Values\;(A)}\frac{S_{v}}{S}Entropy\left(S_{v}\right)$$ (2)

GainRation(S,A)=Gain(S,A)/SplitInfo(S,A) (3)

$$SplitInfo\left(S,A\right)=\sum_{t=1}^{c}\frac{S_{v}}{S}log_{2}(\frac{S_{v}}{S})$$ (4)

### Statistical analysis

The AUC was used to compare the prediction performance of the three data mining models. The classification accuracy referred to the fraction of cases classified correctly. Sensitivity referred to the proportion of positive cases that were classified as positive. Specificity referred to the proportion of negative cases that were classified as negative. The formulas are shown as follows, where TP, FP, TN, FN represent true positives, false positives, true negatives, and false negatives, respectively. The AUC value of ANN can be interpreted

Accuracy=(TP+TN)/(TP+FP+TN+FN)

Sensitivity=TP/(TP+FN)

Specificity=TN/(FP+TN)

## Results

**Fig 3:**
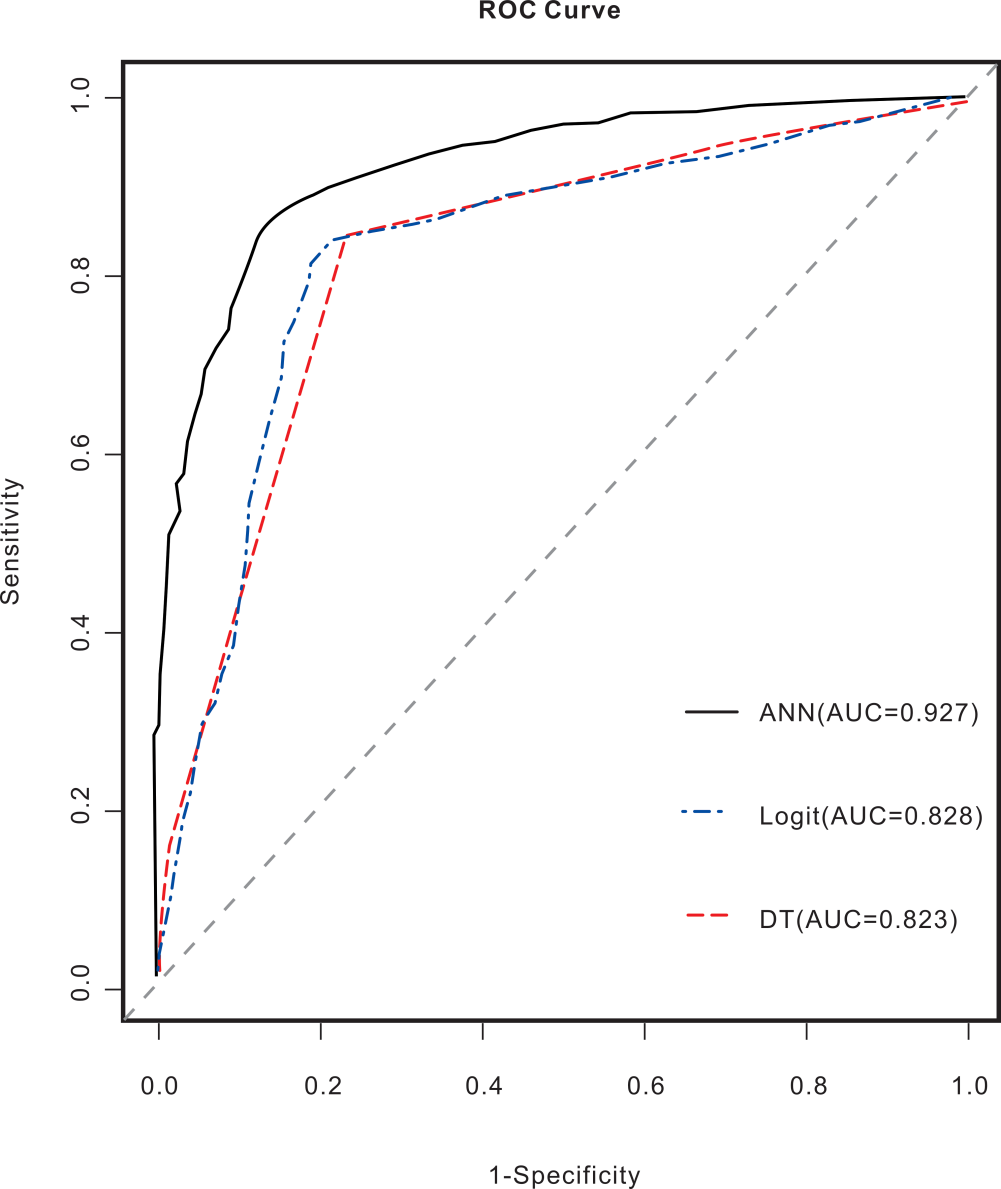
ROC curves and AUC values for the advanced schistosomiasis prognosis models constructed with the training groups using the ANN, DT, and LR models. The AUC value for the prognosis of patients with advanced schistosomiasis was 0.927 for the ANN model, 0.828 for the LR model, and 0.823 for the DT model. The AUC value of the ANN model was superior to those of the DT and LR models.

**Fig 4:**
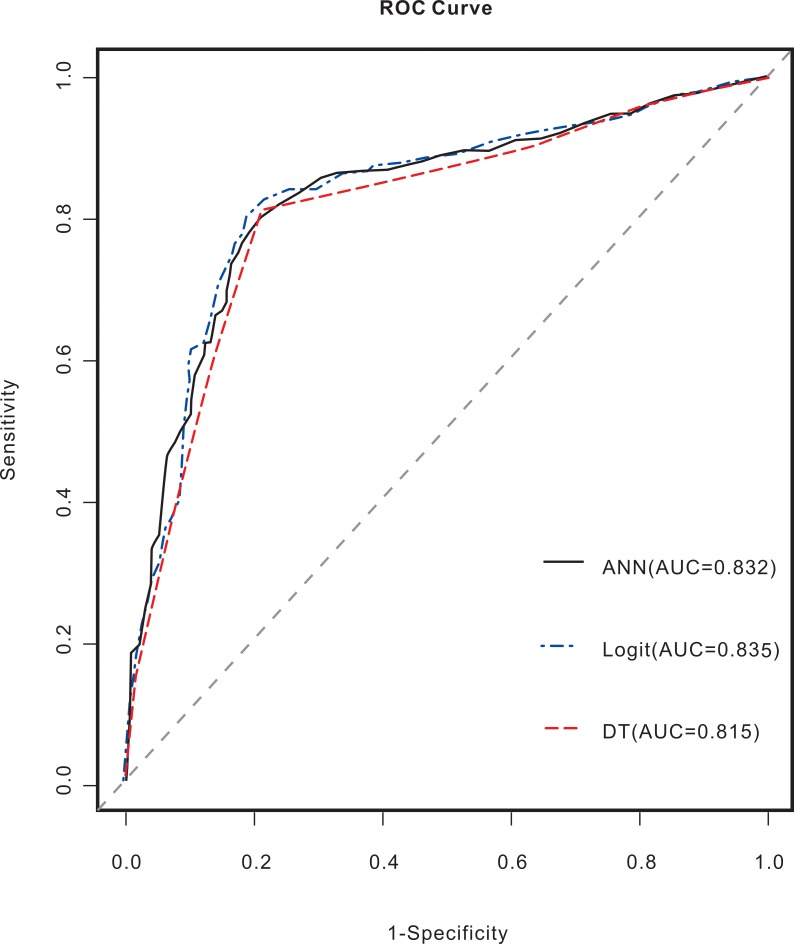
ROC curves and AUC values for the advanced schistosomiasis prognosis models constructed with the validation groups using the ANN, DT, and LR models. The AUC value for the prognosis of patients with advanced schistosomiasis was 0.832 for the ANN model, 0.835 for the LR model, and 0.815 for the DT model. The AUC values of the ANN, DT, and LR models were approximate.

For the training and validation group, the ROC curves for the ANN, LR, and DT models are shown in Figs 3 and 4. In the training group, the AUC value for the prognosis of patients with advanced schistosomiasis was 0.927 for the ANN model, 0.828 for the LR model, and 0.823 for the DT model. The AUC values of the ANN model were superior to those of the DT and LR models. In the validation group, the AUC value for the prognosis of patients with advanced schistosomiasis was 0.832 for the ANN model, 0.835 for the LR model, and 0.815 for the DT model. The AUC values of the ANN, DT, and LR models were approximate.

The performance comparison of the three models in the two groups is listed in **Table 2**. We evaluate the differences in order to see whether there was significance. AUC value could be shown as the normalized Mann–Whitney U statistics. Concerning the normalization denominator is universal for all models, we could thus show the superiority by the AUC value from nonparametric test perspective. Specifically, given the true label of each sample, the larger AUC value, the lager Mann–Whitney U statistics, the better classified capability of the model. We additionally conduct two pairwise tests for AUC values to substantiate the superiority.

**Table 2:** Performance comparison of the three models in two groups.

	Training group			Validation group		
	ANN	DT	LR	ANN	DT	LR
AUC	0.9267	0.8229	0.8276	0.8318	0.8148	0.8349
Sensitivity	0.7527	0.6734	0.6865	0.6442	0.6360	0.6745
Specificity	0.9207	0.8944	0.8388	0.8877	0.9005	0.8465
Accuracy	0.8660	0.8194	0.7990	0.8032	0.8032	0.7992

For ANN and DT, the result shows the difference is significant. (Z = 15.742,P = 0.000).For ANN and LR, we obtain the similar result as following.(Z = 15.117,P = 0.000)

## Discussion

Advanced schistosomiasis, resulting from either repeated infection or acute infection without chemotherapy, is the most severe form of schistosomiasis and clinically presents with portal hypertension [23], periportal liver fibrosis, spleen enlargement, congestion, and other serious conditions [24–26].

Data mining systems aim to extract implicit, previously unknown and potentially valuable relationships and patterns from large amounts of data to provide clear and useful information through advanced processes of selecting, exploring, and modeling [27, 28]. Recent years have seen a rapid development of data mining technology [29, 30].Currently, predictive models are being used in the clinical setting to improve diagnostic and prognostic accuracy and enhance clinical decision-making [28, 31]. Of these predictive models, LR, ANN, and DT models are among the most widely used models for predicting a patients’ prognosis [14, 32–34]. However, little research has been conducted on the use of data mining methods to establish predictive models for prognosis of advanced schistosomiasis. Thus, the current study used data from the Hubei Institute of Schistosomiasis Prevention and Control to develop and compare three predictive models in their ability to predict the prognosis of patients with advanced schistosomiasis.

One of the most attractive features of ANN is the system’s ability to apply machine learning, also referred to as training. ANNs can continuously adjust parameters, such as connection weights, and store the sample set as a connection weight matrix under circumstance of external environment stimulation, such as the input of the sample set. When the ANN accepts the input again, the system can provide the appropriate output. In the present study, there were many neurons in the model and the sample size had rigorous requirements. Therefore, only the variables that were selected by single factor analysis and closely related to the prognosis of advanced schistosomiasis were used as input variables. A good predictive model can distinguish population at high risk from the one at low risk, which is so called discrimination. Discrimination is generally expressed as the area under the ROC curve, referred to as AUC. The higher the AUC value, the better the model can discriminate between high and low risk groups. Due to the serious adverse prognosis of advanced schistosomiasis patients, the sensitivity of the predictive model should be as high as possible in order to avoid false negatives on condition that the discrimination of the model is fine (e.g. AUC≥0.75).

Data from the designated training set was then used to evaluate the ANN model, and the prediction accuracy of the ANN model was 0.8660, which was better than the LR model (0.7990,) and the DT model (0.8194). The AUC of the ANN, LR, and DT models was 0.9267, 0.8276, and 0.8229, respectively, which indicates that the ANN model had the best prediction performance by Mann–Whitney U test.

In comparison to the LR and DT models, the ANN model had the best fitting effect for the relationship between advanced schistosomiasis and pathogenic factors. Schistosomiasis’ pathogenesis of disease is a complicated process influenced by multiple factors; thus, the use of traditional LR models to predict the development of disease is significantly limited by the inability to determine effects of multiple co-linearity between the independent variables. DT models can be easily applied to discrete values, but when there are more attribute values, the effect may be poor [35]. While ANN models can handle more attribute values, they have the potential to over-fit effects and their network training speed can decrease when there are more independent variables [36].

Despite its limitations, the LR model has been widely adopted because it offers other advantages [37, 38]. LR models have the function of discrimination and prediction and LR models are suitable for qualitative and semi-quantitative indicators [39]. In addition, LR models can use log transformation to convert nonlinear relationships between dependent variables and independent variables into linear relationships, which has less restriction conditions and a relatively low requirement of data types. To build predictive models, LR frameworks can automatically select highly correlated indices to be included as independent variables in the equation, which makes LR models convenient, feasible, and easy to popularize[40, 41]. It should be noted that once we develop a LR model in medical practice, it always means the LR model for every disease itself rather than for any disease.

In comparison to LR models, DT models can not only detect statistically significant risk factors, the model can also intuitively compare the intensity of various risk factors on the prognosis of patients with advanced schistosomiasis [42, 43]. The DT algorithm can simultaneously handle diverse types of data and missing data values without having to address the parameters in advance. DT models have a fast training speed, high classification efficiency, and ability to handle large sets of complex non-linear data [44–46].

ANN simulates the function and structure of biological neural network to establish non-linear mathematical models with strong fault tolerance, adaptiveness, nonlinear comprehensive reasoning ability, and the powerful ability to solve co-linearity and interactions between variables [47, 48]. Although complex relationships often exist between output and input factors in the medical field, ANNs have been used in clinical settings to effectively solve this issue and successfully applied to large and complex sample statistics.[49–51]. ANN models can not only realize the objective detection and classification of disease, but they can also improve the efficiency of disease prognosis and differential diagnosis. While the predictive ability of ANNs has many advantages, the model still has several limitations. First, the network changes with the setting of parameters, functions, and initial values. The correctness of these settings lack a theoretical basis, as the settings can only be determined by experience and repeated tests. Second, unlike the LR model, the ANN model does not have a recognized model of input variable access and elimination. Third, as a result of their structure, ANN models do not provide any medical explanation pertaining to each independent variable; thus, the hypothesis test methods, confidence intervals, and other issues require additional research [52, 53].

The advantages and disadvantages between these models on the implementation of them in the medical practice are noteworthy. A study that used ANN models and generalized additive models (GAM) to estimate glomerular filtration rate (GFR) in patients with chronic kidney disease found that the advantage of ANN is obvious only when multiple variables added to the model, especially the multicollinearity existed [54]. ANN is difficult to solve the problem of internal authenticity (repeatability) within the model due to the single data set source. However, the advantages of the ANN model over LR were also demonstrated: dealing with noise and incomplete input variables, high fault tolerance and good generalizability. LR model still plays an important role in the study of prognosis of disease due to its better interpretability. In a study that used large national samples to find the cause of arthritis pain, the DT model incorporated more than 200 variables with a high accuracy of 85.68% [55]. In the era of big data, the DT model facilitates algorithms transforming from hypothesis-driven to data-driven. Like ANN model, the robustness of DT model is better when there are more covariables [56]. Tree models can produce visual classification rules which are closer to people's way of thinking. However, DT model also has its disadvantages such as potentially introducing bias due to division of the tree every time, with the other drawbacks of high variance and instability.

The present study constructed three predictive models—the ANN model, the LR model, and the DT model—to predict advanced schistosomiasis prognosis. While each of the predictive models proved effective and had their own advantages, the ANN model outperformed the LR and DT models in terms of AUC and sensitivity. However, to achieve the highest level of prediction accuracy and better assist medical professionals, the three predictive models should be applied after model comparison.

## Supporting information

S1 Table (XLS)

S2 Table (XLS)

S1 Dataset (ZIP)

S2 Dataset (ZIP)
