# Supplementary material for: Comparison of three data mining models for prediction of advanced schistosomiasis prognosis in the Hubei province
Source: PLoS Negl Trop Dis. 2018 Feb 15;12(2):e0006262. doi: 10.1371/journal.pntd.0006262 (PMC5831639; doi:10.1371/journal.pntd.0006262)
Supplement: S1 Dataset — (ZIP) [file pntd.0006262.s003.zip › grouped data/training group.docx]

Occupation, Annual Income, BMI, Viability, Nourishment, Diagnostic Evidence1, Diagnostic Evidence2, Prior treatment, History of splenectomy, History of ascites,

Other disease, The extent of ascites, Clinical classification, Type of treating patients, Means of treatment, Cost of treatment

1,1,1,1,2,3,2,1,1,1,2,0,1,1,1,1,1

1,0,2,1,2,2,1,1,1,1,0,0,2,1,1,0,1

1,1,3,1,2,3,1,1,0,1,1,0,2,1,1,1,0

1,0,3,1,3,2,1,0,0,1,0,0,2,2,1,1,1

1,0,1,1,2,1,1,1,1,1,2,0,2,1,1,0,1

1,1,0,1,2,3,1,1,1,1,2,0,2,1,1,0,1

1,0,2,1,2,1,1,0,0,1,1,0,2,2,1,1,0

1,0,3,1,2,1,1,1,0,1,0,0,2,1,1,1,0

1,1,1,1,2,1,1,1,0,1,0,0,2,1,1,1,0

1,1,1,1,2,1,1,1,0,1,0,0,2,1,1,0,1

1,0,1,1,2,1,1,1,0,1,0,0,2,1,1,1,0

1,0,1,1,2,2,1,1,0,1,0,0,2,1,1,1,1

1,0,1,1,2,1,2,1,1,1,0,0,2,1,1,0,1

1,1,1,1,2,2,1,1,0,1,0,0,2,1,1,0,1

1,1,1,1,2,1,1,1,1,1,2,0,2,1,2,1,0

1,0,1,1,2,1,1,1,0,1,2,0,2,2,1,1,0

1,0,1,1,2,2,1,1,0,1,0,0,2,1,1,1,1

1,0,1,1,2,1,1,1,0,1,0,0,2,1,1,1,1

12,0,1,1,2,3,2,1,1,1,0,0,2,1,1,1,1

1,0,0,1,2,3,2,1,1,1,2,0,2,1,1,0,1

1,0,1,1,2,2,1,1,0,0,0,0,2,2,1,1,1

1,0,0,1,2,3,1,1,0,1,6,0,2,1,1,1,0

1,0,1,1,1,4,1,1,0,1,0,0,2,1,1,1,0

1,1,1,1,2,3,1,1,0,1,0,0,2,1,1,1,0

1,1,1,1,1,1,1,1,0,1,0,0,2,1,1,1,1

1,1,3,1,2,1,1,1,0,1,0,0,2,1,1,0,1

1,0,2,1,2,1,1,1,1,1,1,0,2,1,1,1,0

1,0,2,1,2,3,1,1,0,1,0,0,2,1,1,1,1

1,0,1,1,2,2,2,1,1,1,2,0,1,1,1,1,0

1,0,2,1,1,1,1,1,0,1,0,0,2,1,1,1,1

1,0,3,1,2,2,1,1,0,1,0,0,2,1,1,1,0

1,0,1,1,2,3,1,1,0,1,5,0,2,1,1,1,1

1,0,0,1,2,3,1,1,1,1,2,0,2,1,1,0,1

1,1,1,1,2,3,1,1,0,1,1,0,2,1,1,0,1

1,1,1,2,2,3,1,1,1,1,2,0,2,1,1,1,1

1,0,1,1,2,1,1,0,0,1,0,0,2,2,1,1,0

1,1,1,1,2,3,2,1,1,1,0,0,2,1,1,0,0

1,1,1,1,2,3,1,1,0,1,2,0,2,1,1,0,1

1,1,2,1,2,3,1,1,0,1,6,0,2,1,1,0,1

2,0,3,1,2,1,1,1,0,1,2,0,2,1,1,0,1

1,1,1,1,2,1,2,1,1,1,0,0,2,1,1,1,1

1,0,3,1,2,1,1,1,0,1,2,0,2,1,1,1,0

1,0,1,1,1,2,1,1,1,1,0,0,2,1,1,1,0

1,0,1,1,2,3,1,1,0,1,1,0,2,1,1,1,1

1,0,3,1,2,1,1,1,0,0,2,0,2,1,1,1,1

1,1,1,1,2,3,1,1,0,1,0,0,2,1,1,1,1

1,0,1,1,2,1,1,1,1,1,2,0,2,1,1,0,1

1,0,1,2,3,1,1,1,1,1,0,0,2,1,1,0,1

1,1,0,1,2,1,2,1,1,1,2,0,2,1,1,0,0

1,0,1,2,2,2,1,1,1,1,0,0,2,1,1,0,0

1,1,0,1,2,3,2,1,1,1,6,0,2,1,1,1,0

1,0,1,1,2,1,2,1,1,1,2,0,2,1,1,0,1

1,0,1,2,2,2,1,1,0,1,0,0,2,1,1,1,0

1,0,3,1,2,2,1,1,0,1,1,0,2,1,1,1,0

1,1,1,1,2,3,1,1,0,1,4,0,2,1,1,1,1

1,0,1,1,2,1,1,1,0,1,0,0,2,1,1,1,0

1,0,1,1,2,2,1,1,0,1,0,0,2,1,1,1,1

7,0,3,1,1,1,1,1,1,1,0,0,2,1,1,1,0

1,0,1,1,2,1,1,1,1,1,2,0,2,1,1,1,0

1,0,3,1,2,1,1,1,0,1,1,0,2,1,1,1,0

1,0,0,1,2,2,1,0,1,1,0,0,2,1,1,1,0

1,0,1,1,2,1,1,1,0,1,0,0,2,1,1,0,1

1,0,1,1,1,1,1,1,0,1,0,0,2,1,1,1,1

1,0,1,1,2,1,1,1,0,1,0,0,2,1,1,1,1

1,0,1,1,2,1,1,1,1,1,2,0,1,1,1,1,0

1,0,3,1,2,1,2,1,1,1,0,0,2,1,1,0,1

1,1,1,1,2,1,1,1,0,1,0,0,2,1,2,1,1

1,0,1,1,2,1,1,1,0,1,2,0,2,1,1,1,0

1,0,0,1,2,3,1,1,0,1,6,0,2,2,1,1,0

1,0,2,1,2,2,1,1,0,1,2,0,1,1,1,1,0

1,0,1,1,2,1,1,0,0,0,2,0,2,1,1,1,1

1,1,1,1,2,3,1,1,1,1,0,0,2,2,2,0,0

1,1,1,1,2,3,1,1,1,1,2,0,2,1,1,0,1

1,1,1,1,1,3,2,1,1,1,2,0,2,1,1,0,0

1,0,0,1,2,3,2,1,1,1,2,0,2,1,1,1,0

1,1,1,1,2,3,2,1,1,1,0,0,2,1,1,0,0

1,1,3,1,1,3,1,1,0,1,0,0,2,3,1,1,0

1,0,1,1,2,1,2,1,1,1,0,0,1,1,1,1,1

1,1,3,1,1,1,1,1,1,1,0,1,2,1,1,0,0

1,0,1,1,3,1,1,0,1,1,0,0,2,1,1,1,0

1,1,1,1,2,1,1,1,1,1,0,0,2,1,1,0,0

1,1,0,1,2,3,1,1,1,1,6,0,2,1,1,0,1

1,0,1,1,2,2,1,1,1,1,1,0,2,1,1,1,0

1,0,1,1,2,3,1,1,1,1,2,0,2,1,1,1,0

1,0,0,1,2,1,2,1,1,1,2,0,2,1,1,1,0

1,0,1,1,2,3,1,1,0,1,0,0,2,1,1,1,1

1,1,1,1,2,1,2,1,0,0,0,0,1,1,1,1,0

1,0,1,1,3,1,1,1,1,1,1,0,2,1,1,1,0

1,0,1,2,2,1,1,1,0,1,0,0,2,1,1,0,1

1,0,0,1,2,3,2,1,0,1,2,0,1,1,1,1,0

1,0,1,1,2,1,1,1,0,1,0,0,2,1,1,1,1

1,0,3,1,2,1,2,1,1,1,1,0,1,2,1,1,1

1,0,2,1,2,1,1,1,0,1,0,1,2,1,1,0,0

1,0,1,1,2,1,1,1,0,1,0,0,2,1,1,1,0

1,1,1,1,1,1,1,1,0,1,0,0,2,1,1,0,1

1,0,0,1,2,1,1,1,1,1,1,0,2,1,13,0,1

1,1,1,1,2,3,1,1,1,1,0,0,2,1,1,0,1

1,0,1,1,2,3,1,1,0,1,6,0,2,1,1,1,0

1,0,1,1,2,1,1,1,0,1,0,0,2,1,1,1,1

1,0,1,1,2,1,2,1,1,1,0,0,1,1,1,1,0

1,0,1,1,2,1,1,1,0,1,0,0,2,1,1,1,0

1,0,2,1,2,4,1,1,0,1,0,0,2,1,1,1,1

1,0,1,1,2,3,2,1,0,0,0,0,1,1,1,1,1

1,0,1,1,2,1,2,1,1,1,0,0,1,1,1,1,0

1,1,1,1,2,2,2,1,1,1,0,0,2,1,1,0,1

1,0,3,1,2,1,1,1,0,0,2,0,2,1,1,1,0

1,0,1,1,2,2,1,1,0,1,0,0,2,2,1,1,0

1,0,1,1,2,1,1,1,0,1,1,0,2,2,1,1,0

8,0,1,1,1,2,1,1,0,1,0,0,2,1,1,1,1

1,0,1,1,1,2,1,1,1,1,0,0,2,1,1,1,0

1,0,2,1,2,1,1,1,1,1,12,0,2,1,1,0,1

1,1,1,1,2,1,1,1,0,1,0,0,2,1,1,1,0

1,0,3,1,2,2,1,1,1,1,1,0,2,1,1,1,0

1,1,1,1,2,3,1,1,1,1,2,0,2,1,1,1,0

1,1,1,1,2,3,1,1,0,1,0,0,2,1,1,1,1

1,1,1,1,2,3,2,1,1,1,4,0,2,1,1,0,1

1,1,1,1,2,1,2,1,1,1,2,0,2,1,1,0,1

1,1,1,1,2,3,1,1,0,1,1,0,2,1,1,0,1

1,0,1,1,2,1,1,1,1,1,1,0,2,1,1,1,1

1,0,1,1,2,1,2,1,1,1,2,0,1,1,1,1,1

1,0,0,1,2,3,1,1,0,1,6,0,2,1,1,1,0

1,0,1,1,2,2,2,1,0,1,0,0,2,1,1,1,1

1,0,3,1,2,2,2,1,0,1,2,0,2,1,1,1,0

1,0,1,1,1,3,1,1,0,1,2,0,2,1,1,1,1

1,0,1,1,2,2,1,1,0,1,0,0,2,1,1,1,1

1,1,1,1,2,3,1,1,0,1,0,0,2,1,1,1,0

1,0,1,1,2,3,1,1,0,1,2,0,2,1,1,1,0

1,0,1,1,2,1,2,1,1,0,0,0,1,1,1,1,1

1,0,3,1,2,1,1,1,0,1,2,0,2,1,1,1,0

1,1,1,1,2,3,1,1,0,1,1,0,2,1,1,1,1

1,1,0,1,2,3,2,1,0,1,5,0,2,1,1,1,0

1,0,2,2,3,2,1,1,0,1,0,0,2,1,1,0,0

1,0,0,1,2,3,1,1,1,1,6,0,2,2,1,1,0

1,1,1,1,1,3,1,1,1,1,6,0,2,1,1,0,0

1,1,0,1,2,3,2,1,0,1,2,0,1,1,1,1,1

1,1,3,1,1,1,1,1,1,1,0,0,2,1,1,0,1

1,0,0,1,2,3,1,1,1,1,2,0,2,1,1,1,0

1,1,2,1,1,3,1,1,0,1,2,0,2,1,1,1,0

1,0,1,1,2,2,1,1,0,1,1,0,2,1,1,1,0

1,0,3,1,2,1,1,1,0,1,0,0,2,1,1,1,1

1,0,2,1,2,1,2,1,1,0,0,0,2,1,1,1,0

1,0,1,1,2,1,1,1,0,1,0,0,2,1,1,1,1

1,0,1,1,2,1,1,0,0,0,0,0,2,1,1,1,1

1,1,1,1,2,2,1,1,0,1,4,0,2,1,1,0,1

1,1,0,1,2,3,1,1,1,1,2,0,5,1,1,0,1

1,0,1,1,2,2,1,1,0,1,6,0,2,1,1,1,0

1,1,1,1,2,3,1,1,0,1,6,0,2,1,1,0,1

2,1,1,1,2,3,1,1,1,1,0,0,2,1,1,0,0

1,0,1,1,2,3,1,1,0,1,1,0,2,1,1,0,1

1,1,1,1,2,3,1,1,1,1,2,0,2,1,1,0,1

1,0,1,1,2,2,2,1,1,1,0,0,1,1,1,1,1

1,0,1,2,3,2,1,1,0,1,0,0,2,1,1,1,1

1,0,1,1,2,2,1,1,1,1,0,0,2,1,1,0,1

1,0,0,1,2,3,1,1,1,1,6,0,2,1,1,1,0

1,1,2,1,2,1,1,1,1,1,2,0,2,1,1,0,0

1,1,3,1,2,2,2,1,1,1,2,0,2,1,1,1,0

1,0,1,1,2,1,2,1,0,1,2,0,2,1,1,1,1

1,0,1,1,3,1,1,1,0,1,6,0,2,1,1,1,1

1,1,1,1,2,3,1,1,0,1,1,0,2,1,1,0,1

1,0,1,1,3,1,2,1,0,0,0,0,2,1,13,0,1

1,0,1,1,2,1,1,1,0,0,2,0,2,1,1,1,1

1,0,3,1,2,2,1,1,1,1,0,0,2,2,1,1,0

1,1,1,1,1,2,2,1,1,0,2,0,2,2,1,0,0

3,1,0,1,1,3,1,1,0,1,5,0,2,1,1,0,1

1,1,1,1,2,3,1,1,1,1,0,0,2,1,1,0,1

1,1,1,1,2,3,1,1,0,1,0,0,2,1,1,0,1

1,0,3,1,1,1,1,1,1,1,0,0,2,1,1,1,1

1,0,3,1,2,2,1,1,0,1,0,0,2,1,1,0,0

1,0,1,1,2,1,1,1,0,1,0,0,2,1,1,1,1

1,0,0,1,2,3,2,1,1,1,6,0,2,1,1,1,0

1,0,1,1,2,1,1,1,0,1,0,0,2,1,1,1,1

1,0,0,1,1,2,1,1,0,1,0,0,2,1,1,1,0

1,0,1,1,2,1,1,1,0,1,0,0,2,1,1,1,0

1,1,2,1,2,3,1,1,0,1,2,0,2,1,1,0,1

1,1,1,1,2,3,1,1,0,1,4,0,2,1,1,1,1

1,0,2,1,2,3,1,1,0,1,0,0,2,1,1,1,1

1,1,0,1,2,3,2,1,0,1,6,0,2,1,1,1,0

1,1,1,1,2,1,1,0,0,1,2,0,2,1,1,0,1

2,1,1,1,2,3,1,1,0,1,1,0,2,1,1,0,1

1,1,1,1,2,3,1,1,1,1,1,0,2,1,1,0,1

1,1,2,1,2,3,1,1,0,1,2,0,2,1,1,1,1

1,0,1,1,2,1,1,1,0,1,2,0,2,1,1,0,0

1,1,1,1,2,3,1,1,0,1,1,0,2,1,1,0,1

1,0,1,1,2,1,1,1,0,1,5,0,2,1,1,1,1

1,0,2,1,2,1,1,1,0,1,1,0,2,1,1,1,0

1,1,1,1,2,3,1,1,0,1,2,0,2,1,1,1,0

1,1,1,1,2,3,1,1,1,1,1,0,2,1,1,0,1

1,1,1,1,2,3,1,1,0,1,0,0,2,1,1,0,1

1,1,1,1,2,3,1,1,0,1,0,0,2,1,1,0,1

1,0,1,1,2,1,2,0,0,0,0,0,1,3,2,1,1

1,0,1,1,2,1,2,1,1,1,0,0,2,1,1,1,0

1,0,1,1,2,3,1,1,0,1,0,0,2,1,1,1,0

1,1,3,1,1,1,1,1,1,1,0,0,2,1,1,0,0

1,0,1,1,2,1,1,1,1,1,0,0,2,1,1,0,1

1,0,3,1,2,1,1,1,0,1,1,0,2,1,1,1,0

1,0,1,1,3,1,1,1,0,1,1,0,2,1,1,1,0

1,1,1,1,2,1,1,1,0,1,0,0,2,1,1,1,0

1,1,2,1,2,1,1,1,0,1,0,0,2,1,1,1,0

1,0,0,1,2,3,1,1,0,1,0,0,2,1,1,1,0

1,0,1,1,2,2,1,1,1,1,2,0,2,1,1,1,0

1,0,1,1,2,3,2,1,0,0,0,0,2,1,1,1,1

1,0,1,1,2,3,1,1,1,1,0,0,2,1,1,1,1

1,1,1,1,2,1,2,1,1,1,0,0,1,1,1,1,0

12,0,1,1,2,2,2,1,1,1,0,0,1,1,1,1,0

1,0,1,1,2,1,2,1,0,0,2,0,2,1,1,0,0

1,1,1,1,2,3,1,1,0,1,1,0,2,1,1,1,0

1,1,1,1,2,3,1,1,0,1,1,0,2,1,1,1,1

1,0,0,1,1,1,1,1,1,1,1,0,2,1,1,1,0

1,1,2,1,1,3,2,1,1,1,2,0,2,1,1,0,0

1,0,0,1,2,1,2,1,1,1,0,0,1,1,1,1,0

1,0,1,1,2,1,1,1,0,1,1,0,2,1,1,1,1

1,1,3,1,1,3,1,0,0,1,6,0,2,1,1,1,0

1,0,0,1,2,3,2,1,1,1,2,0,2,1,1,1,0

1,0,1,1,2,1,1,1,0,1,2,0,2,1,1,1,1

1,0,1,1,1,3,2,1,0,0,2,0,2,1,1,1,1

1,0,1,1,1,3,2,1,0,0,2,0,1,1,1,1,1

1,0,1,1,2,1,2,1,1,1,2,0,2,1,1,1,0

1,1,0,1,2,3,2,1,1,1,2,0,2,1,1,1,0

1,1,1,1,1,3,1,1,0,1,6,0,2,1,1,1,0

1,0,1,1,2,1,1,1,1,1,0,0,2,1,1,1,0

1,1,1,1,2,2,2,1,1,1,1,0,1,1,1,1,0

1,0,1,1,2,1,2,1,1,1,0,0,1,1,1,1,0

1,0,1,1,2,1,1,1,0,1,0,0,2,1,1,1,0

1,0,1,1,2,1,2,1,1,1,2,0,2,1,1,1,0

1,0,1,1,2,1,1,1,0,1,0,0,2,1,1,1,0

1,0,1,1,2,2,2,1,1,1,0,0,1,1,1,1,0

1,0,1,1,1,3,1,1,0,1,6,0,2,1,1,1,0

1,0,1,1,2,1,2,1,1,1,0,0,2,1,13,0,1

1,0,0,1,2,3,1,1,1,1,6,0,2,1,1,1,0

1,0,1,1,2,1,1,1,0,1,1,0,2,1,1,0,0

1,1,1,1,1,4,1,1,0,1,0,0,2,1,1,0,1

1,1,1,1,2,3,1,1,1,1,2,0,2,1,1,1,0

1,1,1,1,2,3,1,1,0,1,4,0,2,1,1,0,1

1,0,1,1,2,1,1,0,0,1,2,0,2,1,1,1,0

1,1,3,1,2,1,1,1,0,1,0,0,2,1,1,1,0

1,0,2,1,2,2,1,1,0,1,1,0,2,1,1,1,0

1,1,2,1,3,3,1,1,1,1,1,0,2,1,1,0,1

1,0,1,1,2,1,1,1,1,0,0,0,2,1,1,0,1

1,0,1,1,2,1,1,1,1,1,2,0,2,1,1,1,0

1,0,1,1,1,1,1,1,0,1,0,0,2,1,2,1,1

1,0,1,1,2,2,1,1,0,1,0,0,2,1,1,1,1

1,0,1,1,2,1,1,1,0,1,6,0,2,1,13,0,1

1,0,2,2,2,2,1,1,1,1,0,0,2,1,1,0,0

2,0,1,1,1,1,1,1,0,1,0,0,2,1,1,1,0

1,1,1,1,2,3,2,1,0,0,0,0,2,1,1,1,0

1,1,3,1,2,3,1,1,0,1,0,0,2,1,1,0,1

1,0,1,1,2,1,1,1,0,1,0,0,2,1,1,1,1

1,0,1,1,2,1,1,1,0,1,0,0,2,1,1,1,1

1,0,1,1,2,3,2,1,1,1,0,0,2,1,1,0,1

1,1,1,1,2,3,1,1,0,1,2,0,2,1,1,0,1

1,0,1,1,2,1,1,1,1,1,2,0,2,1,1,0,1

1,0,1,1,2,1,1,1,1,1,0,0,2,1,1,0,1

1,1,1,1,2,3,1,1,1,1,0,0,2,1,1,0,0

1,1,1,1,2,3,1,1,1,1,2,0,2,1,1,0,0

1,0,1,1,2,1,1,0,1,0,0,0,2,3,1,0,1

1,1,1,1,2,3,2,1,1,1,0,0,2,1,1,0,0

1,1,1,1,2,3,1,1,1,1,0,0,2,1,1,1,1

1,0,3,1,1,1,1,1,0,1,0,0,2,1,1,1,0

1,0,1,1,1,2,1,1,1,1,0,0,2,1,1,1,0

1,0,1,1,2,1,1,1,0,1,0,0,2,1,1,1,1

1,1,1,1,2,2,2,1,1,1,1,0,1,1,1,1,1

1,0,0,1,2,3,1,1,0,1,6,0,2,2,1,1,0

1,1,3,1,2,3,1,1,1,1,2,0,2,1,1,1,0

1,0,0,1,2,3,1,1,1,1,2,0,2,1,1,0,1

1,0,1,1,2,1,1,1,0,1,4,0,2,1,1,1,0

1,0,1,1,1,1,1,1,0,1,0,0,2,3,1,1,0

1,1,1,1,2,1,1,1,0,1,0,0,2,1,1,1,0

1,0,2,1,2,1,1,1,0,1,0,0,2,1,1,0,0

1,0,1,1,2,1,1,1,0,1,0,0,2,1,1,1,0

1,0,2,1,2,1,2,1,0,1,0,0,1,1,1,1,0

1,1,1,1,2,1,1,1,0,1,0,0,2,1,1,1,0

1,0,1,1,1,3,1,1,0,1,6,0,2,1,1,1,0

1,1,1,1,2,2,1,1,0,1,0,0,2,1,1,1,0

1,0,3,1,1,2,1,1,0,1,0,0,2,1,1,0,0

1,1,1,1,1,3,1,1,1,1,0,0,2,1,1,1,1

1,1,2,1,2,1,2,1,1,1,0,0,2,1,1,1,1

1,0,0,1,2,3,1,1,0,1,6,0,2,1,1,1,0

1,1,0,1,2,3,2,1,1,1,6,0,1,1,1,1,0

1,0,2,1,3,1,1,1,1,1,0,0,2,1,1,0,1

1,1,3,1,1,3,2,1,1,1,0,0,2,1,1,1,0

1,1,2,1,2,3,2,1,1,1,0,0,1,1,1,1,1

1,0,1,1,2,1,2,1,0,1,0,0,1,1,1,1,0

1,0,1,1,2,1,1,1,0,1,2,0,2,1,1,1,1

1,1,1,1,1,3,1,1,0,1,0,0,2,1,1,0,1

1,1,2,1,2,1,1,1,0,1,6,0,2,1,1,0,1

1,1,1,1,2,1,1,1,1,1,2,0,2,1,1,0,0

1,0,1,1,2,1,1,1,0,1,0,0,2,1,1,1,0

1,0,2,1,2,1,1,1,0,1,2,0,2,1,1,0,1

1,0,2,1,2,1,1,1,0,1,0,0,2,1,1,1,0

1,1,1,1,1,3,1,1,0,1,0,0,2,1,1,1,0

1,0,3,1,2,1,2,1,1,1,0,0,2,1,1,1,1

1,0,2,1,2,1,1,1,0,1,2,0,1,1,1,1,0

1,0,2,1,2,1,2,1,1,1,0,0,2,1,1,1,0

1,0,0,1,2,1,1,1,0,1,0,0,2,1,1,0,0

1,1,3,1,2,1,1,1,0,1,0,0,2,1,1,1,0

1,0,2,1,2,1,2,1,1,0,0,0,1,1,1,1,0

1,0,1,1,2,1,2,1,1,1,2,0,1,1,1,1,0

1,0,1,1,2,1,1,1,0,1,0,0,2,1,1,1,0

1,0,1,1,2,2,1,1,0,1,2,0,2,1,1,0,1

1,0,1,1,2,2,1,1,0,1,0,0,2,1,1,1,0

1,0,1,1,2,1,1,0,0,1,2,0,2,2,1,1,0

1,0,1,1,1,1,2,1,1,1,0,0,1,1,1,1,0

1,0,1,1,2,1,1,1,1,1,0,0,2,1,1,1,0

1,0,1,1,2,3,1,1,1,1,4,0,2,1,1,0,1

1,0,1,1,2,1,2,1,1,0,0,0,1,1,1,1,0

1,1,1,1,2,3,2,1,0,0,0,0,1,2,1,0,1

1,1,1,1,2,1,2,1,0,1,0,0,2,1,1,0,1

1,0,2,1,2,1,2,1,1,1,2,0,2,1,1,0,1

1,0,1,1,1,3,1,1,0,1,0,0,2,1,1,1,1

1,1,1,1,2,3,1,1,1,1,2,0,2,1,1,0,1

1,1,1,1,2,1,1,1,1,1,0,0,2,1,1,0,1

1,0,1,1,1,1,1,1,0,1,0,0,2,1,1,1,1

1,0,1,1,2,1,1,1,0,1,0,0,2,1,1,1,0

1,0,1,2,3,1,1,0,0,1,6,0,2,1,13,0,1

1,1,2,1,2,2,1,1,1,1,2,0,2,1,1,0,0

1,0,1,1,2,1,1,1,1,1,0,0,2,1,1,0,1

1,0,0,1,2,3,1,1,0,1,2,0,2,1,1,1,1

1,1,1,1,2,1,2,1,1,1,0,0,1,1,1,1,1

1,0,1,1,2,1,2,1,1,1,0,0,2,1,13,0,1

1,0,1,1,3,3,1,1,1,1,6,0,2,1,1,0,1

2,1,3,1,2,3,1,1,1,1,1,0,2,1,1,0,0

1,0,1,1,2,1,1,1,1,1,0,0,2,1,1,1,0

1,0,3,1,2,2,1,1,0,1,1,0,2,1,1,1,0

1,0,0,1,1,1,1,0,0,1,0,0,2,2,1,1,1

1,0,1,1,2,2,1,1,0,1,0,0,2,1,1,1,0

1,0,1,1,2,1,1,1,1,1,0,0,2,1,1,0,1

1,0,1,1,2,1,1,1,0,1,0,0,2,1,1,1,0

1,0,1,1,3,1,2,1,1,1,0,0,2,1,1,1,0

1,0,1,1,3,1,1,1,0,1,0,0,2,1,1,1,1

1,1,1,1,2,2,1,1,0,1,0,0,2,1,1,1,0

1,0,1,1,2,1,1,1,0,1,0,0,2,1,1,1,1

1,0,2,1,2,1,2,1,1,1,0,0,2,1,1,0,1

1,1,1,1,2,2,1,1,0,1,1,1,2,1,1,0,1

1,1,0,1,2,3,1,1,1,1,6,0,2,1,1,0,0

1,0,3,1,1,1,1,1,0,1,2,0,2,1,1,1,0

1,0,1,1,2,1,1,1,0,1,0,0,2,1,1,1,0

1,0,2,1,2,1,1,1,0,1,0,0,2,1,13,0,0

1,0,1,1,1,2,1,0,0,1,4,0,2,2,1,1,0

1,0,1,1,2,1,1,1,0,1,2,0,2,1,1,1,0

1,1,3,1,2,1,1,1,1,1,0,0,2,1,1,1,0

1,1,1,1,2,1,1,1,1,1,0,0,2,1,1,1,1

1,0,3,1,1,2,1,1,0,1,0,0,2,1,1,0,1

1,0,1,1,2,2,1,1,1,1,1,0,2,1,1,1,0

1,1,3,1,2,3,2,1,1,1,0,0,1,1,1,1,1

1,0,2,1,2,2,1,1,0,1,1,0,2,1,1,1,0

1,1,1,1,2,2,1,1,0,1,0,0,2,1,1,1,0

1,1,1,1,2,2,1,1,0,1,1,0,2,1,1,1,0

1,1,1,1,2,3,1,1,1,1,2,0,2,1,1,0,1

1,0,1,1,2,1,2,1,1,1,6,0,2,1,1,1,1

1,0,2,1,2,3,1,1,0,1,1,0,2,1,1,0,1

1,1,1,1,2,1,2,1,0,1,0,0,2,1,1,1,1

1,0,2,1,2,1,1,1,0,1,1,0,2,1,1,1,0

1,0,2,1,3,1,2,1,0,0,4,0,1,1,2,1,0

1,0,1,1,1,4,1,1,0,1,0,0,2,1,2,1,1

1,0,1,1,2,3,1,1,0,1,0,0,2,1,1,0,1

1,0,1,1,3,1,1,1,1,1,0,0,2,1,13,0,1

1,1,1,1,2,3,1,1,1,1,0,0,2,1,1,0,1

1,0,1,1,2,1,2,1,1,1,0,0,1,1,1,1,0

1,0,1,1,2,2,1,0,1,0,0,0,2,1,1,0,1

1,0,0,1,2,2,1,1,0,1,0,0,2,1,1,1,0

1,0,1,1,2,2,1,1,1,1,0,0,2,1,1,0,1

1,0,1,1,2,3,2,1,1,1,1,0,2,1,1,1,0

1,0,1,1,2,1,2,1,1,1,0,0,1,1,1,1,0

1,1,1,1,1,3,2,0,0,0,6,0,1,1,1,1,0

1,0,1,1,3,3,1,1,0,1,2,0,2,1,1,1,0

1,0,1,1,1,1,1,1,0,1,1,0,2,3,1,1,1

1,0,2,1,3,1,1,1,1,1,1,0,2,1,1,0,1

1,0,1,1,2,1,2,1,1,1,0,0,1,1,1,1,0

1,0,1,1,2,1,1,0,1,0,2,0,2,1,1,1,1

1,0,1,1,1,2,1,1,0,1,0,0,2,1,1,0,0

1,0,1,2,3,2,1,1,0,1,0,0,2,1,1,0,0

1,0,1,1,2,3,1,1,0,1,6,0,2,1,13,1,0

1,0,1,1,2,1,1,1,0,1,0,0,1,1,13,0,1

1,0,1,1,2,1,1,1,0,1,6,0,2,1,1,0,0

2,0,1,1,2,1,1,2,1,2,1,0,2,1,1,1,0

1,0,3,1,2,1,1,1,1,1,0,0,2,1,1,1,0

1,0,3,1,2,1,1,1,0,1,6,0,2,1,13,0,1

1,1,0,1,2,3,2,1,1,1,6,0,2,1,1,0,0

1,1,1,1,1,3,1,1,0,1,2,0,2,1,1,1,0

1,1,1,1,2,3,1,1,1,1,6,0,2,1,1,0,1

1,1,0,1,2,3,1,1,1,1,2,0,2,1,1,0,1

1,0,1,1,2,1,2,1,0,1,0,0,1,1,1,0,0

1,1,0,1,2,3,2,1,0,0,2,0,1,1,1,1,0

1,1,1,1,1,1,2,1,1,1,2,0,2,1,1,0,1

1,0,1,1,2,1,1,0,0,1,0,0,2,1,1,1,1

1,0,1,1,2,1,1,0,0,0,1,0,2,1,1,1,1

1,0,1,1,2,1,2,1,1,1,0,0,2,1,1,1,0

1,1,1,1,1,3,2,1,1,1,2,0,2,1,1,0,0

1,1,3,1,2,1,1,1,0,1,2,0,1,1,1,1,1

1,0,1,1,2,3,1,1,0,1,6,0,2,1,13,1,1

1,1,3,1,1,1,1,1,0,1,0,0,2,3,1,1,0

1,1,1,1,2,3,2,1,1,1,4,0,1,1,1,1,1

1,1,2,1,2,3,1,1,0,1,1,0,2,1,1,1,1

1,0,2,1,3,3,2,1,0,1,4,0,2,1,1,1,0

1,0,3,1,2,1,2,1,1,1,0,0,2,1,1,1,0

1,0,3,1,2,1,2,1,1,1,0,0,2,1,1,1,1

1,1,1,1,2,3,1,1,1,1,4,0,2,1,1,0,1

1,1,1,1,2,3,1,1,0,1,2,0,2,1,1,0,1

1,0,2,1,2,3,1,1,0,1,2,0,2,1,1,1,0

2,0,1,1,2,3,1,1,1,1,1,0,2,1,1,0,1

7,0,1,1,2,3,2,1,1,0,2,0,2,1,1,1,1

1,1,0,1,2,3,1,1,1,1,2,0,2,1,1,1,0

1,0,1,1,2,3,1,1,0,1,0,0,2,1,1,1,1

1,0,1,1,1,1,2,0,1,1,0,0,2,1,1,1,0

1,0,3,1,2,1,1,0,0,1,0,0,2,2,1,1,0

1,1,1,1,2,2,2,1,0,0,1,0,1,1,1,1,1

1,1,1,1,2,2,1,1,1,1,0,0,1,1,1,1,0

2,0,1,1,1,3,3,1,0,0,2,0,3,1,1,1,0

1,0,1,1,1,1,1,1,0,1,0,0,2,1,1,1,0

1,0,1,1,1,1,1,1,0,1,1,0,2,1,1,1,0

1,0,1,1,2,1,1,1,0,1,1,0,2,1,1,0,0

1,0,3,1,2,1,1,1,0,1,0,0,2,1,1,1,0

1,1,0,1,2,1,2,1,1,0,0,0,1,1,1,1,1

1,1,1,1,2,3,1,1,0,1,1,0,2,1,1,1,1

1,1,0,1,2,3,1,1,1,1,6,0,2,1,1,0,0

1,0,3,1,1,1,1,1,0,0,2,0,2,1,1,1,1

1,0,1,1,2,1,1,1,0,1,1,0,2,1,1,1,0

1,0,1,1,2,1,2,1,1,1,0,0,2,1,1,0,1

1,0,1,1,1,3,1,1,0,1,0,0,2,1,1,1,0

1,0,2,1,2,1,1,1,1,1,0,0,2,1,1,1,0

1,0,1,1,2,1,1,1,0,1,0,0,2,1,1,1,0

1,1,1,1,2,3,1,1,1,1,4,0,2,1,1,0,1

1,1,1,1,1,4,1,1,0,1,0,0,2,1,1,1,1

1,1,1,1,2,1,2,1,1,0,0,0,1,1,1,1,0

1,0,1,1,2,1,2,1,1,1,0,0,2,1,1,0,1

1,0,1,1,2,1,2,1,1,0,1,0,1,1,1,1,0

1,1,0,1,2,3,2,1,1,1,6,0,2,1,1,0,0

1,0,1,1,2,1,1,1,1,1,1,0,2,1,1,1,0

1,0,1,1,2,2,1,1,1,1,0,0,2,1,1,1,0

1,0,1,1,2,2,1,1,1,1,1,0,2,1,1,1,0

1,0,1,1,2,2,1,1,0,1,2,0,2,1,1,1,0

1,0,2,2,2,1,2,1,1,0,6,0,2,1,13,0,1

1,0,1,1,2,2,1,1,0,1,0,0,2,2,1,1,1

1,0,1,1,2,1,1,0,0,0,0,0,2,1,1,1,1

1,0,1,1,2,1,1,0,1,0,0,0,2,1,1,0,1

1,0,1,1,1,3,1,1,0,1,0,0,2,1,1,1,1

1,1,1,1,1,3,1,1,0,1,6,0,1,1,1,1,0

1,0,0,1,2,1,1,1,0,1,0,0,2,1,1,1,0

1,0,2,1,3,1,1,1,0,1,1,0,2,1,1,0,1

1,0,1,1,2,1,1,1,0,0,2,0,2,1,1,1,1

1,0,1,1,2,1,1,1,0,1,0,0,2,1,1,0,1

1,0,3,1,2,1,1,1,0,0,2,0,2,1,1,1,1

1,0,1,1,2,2,1,1,1,1,0,0,1,1,1,1,0

1,0,1,1,1,1,1,1,0,1,0,0,2,1,1,1,1

1,0,2,2,3,2,1,1,0,1,0,0,2,1,1,0,0

1,1,1,1,2,4,1,1,0,1,4,0,2,1,1,0,1

1,0,1,1,1,1,2,1,1,0,0,0,1,1,1,1,0

1,0,2,1,2,1,2,1,1,1,0,0,1,1,1,1,0

1,0,1,1,2,1,2,1,1,1,0,0,1,1,1,1,0

1,1,3,1,1,1,1,1,1,1,6,0,2,1,1,0,0

1,1,1,1,2,2,2,1,1,1,2,0,1,1,1,1,0

1,0,2,1,2,3,1,1,0,1,6,0,2,1,1,1,0

1,0,1,1,1,1,2,1,1,1,0,0,2,1,1,0,1

1,0,3,1,2,2,1,1,0,1,6,0,2,1,1,1,0

1,0,1,1,2,1,1,0,0,1,1,0,2,3,1,1,0

1,0,3,1,2,1,1,1,0,1,0,0,2,1,2,1,0

1,0,1,1,2,1,1,1,0,1,2,0,2,1,1,1,0

1,0,3,1,2,1,1,1,0,1,0,0,1,2,1,1,0

1,0,1,1,2,1,1,1,0,1,1,0,2,1,1,1,0

1,0,0,1,2,3,2,1,1,1,2,0,2,1,1,1,0

1,0,2,2,3,2,1,1,0,1,0,0,2,1,1,0,0

1,0,2,1,2,1,2,1,1,1,2,0,2,1,1,0,1

1,1,0,1,1,3,1,1,0,2,0,0,2,1,1,0,1

1,0,1,1,2,3,1,1,1,1,1,0,2,1,1,1,0

1,0,3,1,2,1,1,1,0,0,2,0,2,1,1,1,1

1,1,1,1,2,2,1,1,0,1,0,0,2,1,1,0,1

1,0,3,1,2,1,1,1,0,0,2,0,2,1,2,1,1

1,0,1,1,2,1,1,1,0,1,4,0,2,1,1,1,0

1,0,1,1,2,1,1,1,1,1,0,0,2,1,1,0,1

1,0,1,1,2,2,1,1,1,1,2,0,2,1,1,1,1

1,1,1,1,2,3,2,1,1,1,2,0,1,1,1,1,1

1,0,1,1,2,1,2,1,1,1,2,0,1,1,1,1,0

1,0,2,1,2,3,1,1,0,1,0,0,2,1,1,1,1

1,1,1,1,2,1,2,1,0,1,0,0,1,1,1,1,0

1,0,1,1,2,1,2,1,1,1,0,0,1,1,1,1,0

1,0,0,1,1,2,2,1,0,0,1,0,1,1,1,1,0

1,1,1,1,2,3,1,1,0,1,2,0,2,1,1,0,1

1,0,0,1,2,3,1,1,1,1,6,0,2,1,1,1,0

1,1,2,1,2,4,1,1,0,1,2,0,2,1,1,1,1

1,1,1,1,2,3,1,1,0,1,2,0,2,1,1,1,0

1,0,1,1,2,1,2,1,1,1,0,0,1,1,1,0,0

1,0,2,1,3,2,2,0,0,0,0,0,2,1,1,1,0

1,0,1,2,2,2,2,1,0,1,5,0,2,1,1,0,0

1,1,1,1,2,1,2,1,1,1,5,0,1,1,1,1,0

1,0,1,1,2,1,1,1,0,1,0,0,2,1,1,1,1

1,1,1,1,2,1,1,0,0,1,0,0,2,1,1,1,1

1,0,1,1,2,1,1,1,0,1,0,0,2,1,1,1,1

1,1,3,1,2,1,1,1,0,1,0,0,1,2,1,0,1

1,0,1,1,2,2,1,1,0,1,0,0,2,1,1,1,0

1,0,1,1,2,1,1,0,0,0,2,0,2,1,1,1,0

1,0,1,1,1,2,1,1,0,1,0,0,2,1,1,0,0

1,1,2,1,2,3,1,1,1,1,2,0,2,1,1,0,1

1,1,1,1,2,3,1,1,1,1,6,0,2,1,1,0,1

1,0,1,1,2,3,1,1,0,1,0,0,2,1,13,1,1

1,0,2,1,2,1,1,1,0,1,0,0,2,1,1,1,0

1,1,1,1,2,2,1,1,1,1,2,0,1,1,1,1,0

1,0,1,1,2,1,1,1,0,1,0,0,2,1,1,1,0

1,1,1,1,2,1,1,1,0,1,0,0,2,1,1,1,0

1,0,0,1,3,3,2,1,0,1,2,0,2,1,1,1,1

1,0,2,1,3,1,2,1,0,0,0,0,1,1,2,1,0

1,1,1,1,2,1,2,1,1,1,0,0,1,1,1,1,1

1,1,3,1,2,3,1,1,0,1,0,0,2,1,1,0,1

1,0,1,1,2,1,2,1,1,1,0,0,1,1,1,1,1

1,1,1,1,1,1,1,1,0,1,0,0,2,1,1,0,1

1,1,1,1,2,2,1,1,0,1,5,0,2,1,1,0,1

1,0,3,1,2,1,2,1,1,1,0,0,2,1,1,1,1

1,1,1,1,2,3,1,1,1,1,6,0,2,1,1,1,0

1,0,2,1,2,3,2,1,1,1,2,0,1,1,1,1,0

1,0,1,1,3,1,1,1,0,1,1,0,2,1,1,1,1

1,0,0,1,2,3,1,1,0,1,6,0,2,1,1,1,0

1,0,1,1,2,3,1,1,0,1,2,0,2,1,1,1,0

1,0,1,1,2,1,2,1,1,0,0,0,1,1,1,1,1

1,0,1,1,2,1,2,1,1,1,2,0,1,1,1,1,1

1,0,1,1,2,3,1,1,0,1,2,0,2,1,1,1,1

1,0,1,1,2,1,1,1,0,1,1,0,2,1,1,1,1

1,0,1,1,2,1,2,1,0,0,1,0,1,1,1,1,0

1,1,1,1,2,1,2,1,1,1,6,0,2,1,1,1,0

1,0,1,1,2,1,1,1,0,1,0,0,2,1,1,1,0

1,0,2,1,2,3,1,1,1,1,0,0,2,1,1,1,0

1,0,0,1,2,3,1,1,0,1,0,0,2,1,1,1,0

1,0,1,1,2,2,2,1,1,1,0,0,1,1,1,0,0

1,1,1,1,2,3,1,1,0,1,0,0,2,1,1,0,1

1,0,1,1,2,1,1,1,1,1,1,0,2,1,1,0,0

1,0,0,1,2,1,1,1,0,1,1,0,2,1,1,1,1

1,0,1,1,2,1,1,1,0,0,2,0,2,1,1,1,0

1,1,1,1,2,2,2,1,1,1,1,0,2,1,1,1,1

1,0,1,1,1,2,1,1,0,1,0,0,2,1,1,1,1

1,0,3,1,2,2,1,1,0,1,1,0,2,1,1,1,0

1,0,1,1,2,1,1,1,0,1,0,0,2,1,1,1,1

1,0,1,1,2,1,1,1,0,0,2,0,2,1,2,1,0

1,0,1,1,2,1,1,1,1,1,0,0,2,1,1,1,0

8,0,1,1,3,2,1,1,0,1,0,0,2,1,1,1,1

1,1,1,1,2,3,1,1,1,1,0,0,2,1,1,1,1

1,1,2,1,2,3,1,0,0,1,1,0,2,3,1,1,1

1,0,3,1,2,2,2,1,1,1,2,0,1,1,1,1,0

1,0,3,1,2,4,2,1,1,1,0,0,2,1,2,0,0

1,0,3,1,2,1,2,1,0,1,0,0,2,1,1,1,1

1,0,1,1,2,2,1,1,1,0,1,0,2,1,1,1,0

1,1,1,1,2,2,2,1,1,1,0,0,1,1,1,1,0

1,0,0,1,2,3,1,1,1,1,6,0,2,1,1,1,0

1,1,0,1,2,1,2,1,1,1,0,0,1,1,1,1,0

1,1,0,1,2,3,2,1,0,1,2,0,1,2,1,1,0

1,0,1,1,1,2,2,1,1,1,2,0,2,1,1,1,1

1,0,1,1,2,2,1,1,1,1,0,0,2,1,1,1,0

1,0,0,1,2,3,1,1,1,1,2,0,2,2,1,1,0

1,0,1,1,1,1,1,1,0,1,0,0,2,1,1,1,0

1,0,1,2,3,2,1,1,0,1,0,0,2,1,1,0,1

1,0,1,1,2,2,1,1,0,1,1,0,2,1,1,1,0

1,1,3,1,2,1,1,1,0,1,1,0,2,1,1,1,1

1,0,3,1,1,1,2,1,0,0,6,0,1,1,1,1,0

1,1,1,1,2,3,1,1,0,1,0,0,2,1,1,1,1

1,1,1,1,2,3,1,1,0,1,6,0,2,1,1,0,1

7,1,1,1,2,1,1,1,0,1,0,0,2,1,1,0,0

1,1,3,1,1,1,1,1,0,1,0,0,2,1,1,1,1

1,0,1,1,2,1,2,1,0,1,0,0,2,1,1,1,0

2,1,1,1,2,1,2,1,1,1,0,0,2,1,1,0,1

1,0,1,1,1,1,1,1,1,1,6,0,1,1,1,1,0

1,1,1,1,2,3,1,1,0,1,0,0,2,1,1,1,0

1,1,2,1,2,1,2,1,0,1,0,0,2,1,1,1,1

1,0,2,1,2,3,1,1,0,1,2,0,2,1,1,1,0

1,0,1,1,2,2,1,1,0,1,0,0,1,1,1,1,1

1,0,1,1,2,1,1,1,0,1,0,0,2,1,1,1,1

1,0,1,1,2,1,2,1,0,0,0,0,2,1,13,0,1

1,0,2,2,13,2,1,1,0,1,0,0,2,1,1,0,0

1,1,1,1,2,2,1,1,1,1,12,0,2,1,1,1,0

1,1,1,1,2,3,1,1,0,1,0,0,2,1,1,1,1

1,1,0,1,2,3,2,1,1,1,2,0,2,1,1,0,0

1,1,1,1,2,1,2,1,1,1,0,0,2,1,1,0,0

1,0,1,1,2,2,2,1,1,1,2,0,2,1,1,0,0

1,0,1,1,2,4,1,1,1,1,0,0,2,1,2,0,1

1,0,3,1,2,1,1,1,0,1,1,0,2,1,1,1,1

1,1,1,1,2,3,1,1,0,1,0,0,2,1,1,1,0

1,0,1,1,2,1,1,1,1,1,2,0,2,1,1,1,0

1,0,1,1,2,3,1,1,0,1,4,0,2,1,1,1,1

1,1,1,1,2,3,1,1,0,1,2,0,2,1,1,0,1

1,0,0,1,2,1,1,1,0,1,0,0,1,2,1,1,0

1,0,1,1,2,2,1,1,0,0,0,0,2,1,1,1,0

1,0,1,1,2,3,2,1,1,1,0,0,1,1,1,1,0

1,0,1,1,2,1,1,1,1,1,0,0,2,1,1,1,1

1,1,1,1,2,1,2,1,1,1,2,0,2,1,1,1,1

1,0,1,1,2,2,1,1,1,1,0,0,2,2,1,0,1

1,0,1,1,2,1,1,1,1,1,0,0,2,1,13,0,0

1,1,1,1,2,3,1,1,0,1,2,0,2,1,1,0,1

1,1,1,1,2,1,2,1,0,0,0,0,2,1,2,0,1

1,0,1,1,2,1,1,1,0,1,1,0,2,1,1,1,0

1,0,2,1,1,4,1,1,0,1,0,0,2,1,1,1,0

1,0,1,1,3,1,2,1,1,1,0,0,1,1,2,1,0

1,0,2,1,4,1,1,1,0,1,0,0,2,1,1,1,1

1,0,1,1,1,2,1,1,0,1,0,0,2,1,1,1,0

1,1,2,1,2,3,1,1,0,1,0,0,2,1,1,1,0

1,0,1,1,2,3,2,1,1,0,0,0,1,3,1,1,1

1,1,1,1,1,2,2,1,0,1,0,0,2,3,1,0,1

1,0,1,1,2,1,1,1,1,1,0,0,1,1,1,1,1

1,0,3,1,2,3,1,1,0,1,0,0,2,1,1,1,1

1,1,0,1,2,3,1,1,0,1,2,0,2,1,1,1,1

1,0,1,1,2,1,1,0,1,1,0,0,2,1,1,1,0

1,1,1,1,2,1,1,1,0,1,1,0,1,1,1,1,0

1,1,1,1,1,2,1,1,0,1,0,0,2,3,1,0,1

1,1,3,1,2,3,1,1,0,1,1,0,2,1,1,0,1

1,1,1,1,2,4,1,1,0,1,2,0,2,1,2,1,0

1,0,1,1,2,1,1,1,0,1,0,0,2,1,1,1,1

1,0,1,1,3,1,1,1,1,1,0,0,2,1,1,1,0

1,1,0,1,2,3,1,1,0,1,6,0,2,1,1,1,0

1,0,2,1,2,2,2,1,1,1,2,0,1,1,1,1,0

1,1,1,1,2,3,1,1,0,1,6,0,2,1,1,0,1

1,0,1,1,2,1,1,1,1,1,0,0,1,1,1,1,0

1,0,1,1,2,1,2,1,0,1,0,0,1,1,1,1,0

2,0,1,1,1,1,1,1,0,1,0,0,2,1,1,1,1

1,0,1,1,2,2,2,1,1,1,1,0,1,1,1,1,0

1,0,2,1,3,1,1,1,0,1,1,0,2,1,1,1,1

1,1,3,1,2,3,1,1,0,1,1,0,2,1,1,0,1

1,0,0,2,2,3,1,1,1,1,6,0,2,2,1,0,0

1,0,1,1,2,2,1,1,0,1,5,0,2,1,1,1,1

1,1,1,1,2,1,1,1,0,1,0,0,2,1,1,0,1

1,1,1,1,2,2,1,1,0,1,0,0,2,1,1,1,1

1,0,2,1,3,1,2,1,1,1,0,0,1,1,1,1,0

1,0,0,1,2,3,1,1,0,1,6,0,2,1,13,1,1

1,1,1,1,2,1,1,1,0,1,0,0,2,1,1,0,1

1,0,2,1,2,3,1,1,0,1,0,0,2,1,13,0,0

1,1,3,1,1,3,2,1,1,1,0,0,5,1,1,0,1

1,1,2,1,2,3,1,1,0,1,6,0,2,1,1,0,0

1,1,1,2,2,1,1,1,0,0,0,0,2,1,1,0,0

1,1,1,1,2,2,2,1,1,1,0,0,2,1,1,0,0

1,1,2,1,2,3,1,1,0,1,6,0,2,1,1,0,1

1,0,3,1,2,1,1,1,0,0,0,0,2,1,1,1,0

8,0,1,1,1,2,1,1,0,1,6,0,2,1,1,1,0

1,1,1,1,2,4,2,0,1,1,5,0,2,1,1,0,1

1,1,0,1,2,3,1,1,0,1,6,0,2,1,1,1,0

1,0,0,1,2,3,2,1,0,1,6,0,2,1,1,1,0

1,0,1,1,2,1,1,0,0,0,0,0,2,1,1,1,1

1,0,1,1,2,2,1,1,1,1,2,0,2,1,1,0,1

1,0,1,1,2,3,1,1,0,1,0,0,2,1,1,1,0

1,1,2,1,2,2,2,1,1,0,2,0,2,1,1,1,1

1,0,1,1,2,2,1,1,1,1,2,0,2,1,1,1,0

1,0,1,1,1,3,1,1,0,1,6,0,2,1,1,1,1

1,1,1,1,3,3,1,1,1,1,1,0,2,1,1,0,1

1,0,1,1,2,2,2,1,1,0,1,0,1,1,1,1,0

1,0,1,1,2,4,1,1,0,1,2,0,2,1,1,1,0

1,0,1,1,3,1,1,1,0,1,1,0,2,1,1,1,0

1,1,1,1,2,1,2,1,0,2,0,0,1,1,1,1,0

1,1,1,1,2,1,1,1,0,0,0,0,2,1,1,1,1

1,1,2,1,2,1,1,1,0,1,0,0,2,1,1,0,1

1,0,2,1,1,3,2,1,0,0,2,0,2,1,1,0,0

1,1,1,1,2,3,1,1,1,1,1,0,2,1,1,0,1

12,0,2,1,3,1,2,1,1,0,0,0,1,1,1,1,0

1,1,3,1,2,1,1,1,0,1,1,0,2,1,1,1,0

1,0,3,1,2,1,2,1,1,1,1,0,1,1,1,1,0

1,0,1,1,2,2,2,1,1,1,2,0,1,1,1,1,0

1,1,2,1,2,2,1,1,1,1,0,0,1,1,1,1,0

1,1,1,1,2,2,1,1,1,1,0,0,1,1,1,0,1

1,0,1,1,2,1,1,1,0,1,0,0,2,2,1,1,0

1,0,1,1,2,2,1,1,0,1,1,0,2,1,1,1,0

1,0,1,1,2,2,1,1,0,1,4,0,2,1,1,0,0

1,1,1,1,2,1,1,1,1,1,0,0,2,1,1,0,0

1,0,1,1,2,3,1,1,0,1,1,0,2,1,1,1,0

1,1,0,1,2,1,2,1,1,1,2,0,1,1,1,1,1

1,0,3,1,1,1,1,1,1,1,1,0,2,1,1,1,1

1,1,0,1,2,3,1,1,1,1,2,0,2,1,1,0,0

1,1,0,1,2,3,2,1,1,1,2,0,2,1,1,0,0

1,0,1,1,1,1,1,1,0,1,0,0,2,1,1,1,0

1,0,3,1,1,3,2,1,0,1,2,0,1,1,1,1,0

1,0,3,1,2,2,1,1,1,1,0,0,2,1,1,0,0

1,0,1,1,1,3,1,1,0,1,0,0,2,1,1,1,0

1,1,1,1,2,2,2,1,1,1,0,0,2,1,1,0,0

1,1,1,1,2,1,1,1,1,1,0,0,2,1,1,1,0

1,0,1,1,1,3,2,0,0,0,2,0,1,1,1,1,0

1,1,1,1,2,1,1,1,1,1,2,0,1,1,1,1,0

1,1,1,1,2,1,1,1,0,0,0,0,2,1,1,0,1

1,0,0,1,2,3,2,1,1,1,2,0,2,1,1,1,0

1,0,1,1,2,1,1,1,1,1,0,0,2,1,13,0,1

1,0,2,2,3,1,1,1,0,0,0,0,1,1,1,0,0

1,0,1,1,2,1,1,1,0,1,0,0,1,1,1,1,0

1,0,1,1,3,1,1,1,1,1,6,0,2,1,13,0,1

1,0,1,1,2,2,1,1,0,1,0,0,2,1,1,1,0

1,1,1,1,2,3,1,1,0,1,2,0,2,1,1,1,0

1,1,1,1,2,3,2,1,1,1,0,0,1,1,1,1,1

1,0,3,1,2,1,1,1,0,1,0,0,2,1,1,1,1

1,1,3,1,2,1,1,1,1,1,2,0,2,1,1,0,1

1,0,1,1,2,1,2,1,0,1,0,0,2,1,1,1,0

1,0,3,1,1,3,1,0,0,1,6,0,2,3,1,1,0

1,1,1,1,2,1,1,1,0,1,0,0,2,1,1,0,1

1,0,1,1,2,2,1,1,0,1,0,0,2,1,1,1,1

1,0,1,1,2,3,1,1,0,1,0,0,2,1,1,0,0

1,0,1,1,2,1,2,1,0,1,0,0,2,1,1,1,0

1,1,3,1,2,1,1,1,0,1,0,0,2,1,1,1,0

1,0,1,1,2,1,2,1,1,1,2,0,2,1,1,1,0

1,0,2,1,2,2,2,1,1,1,0,0,1,1,1,1,0

1,0,1,1,2,1,2,1,1,1,0,0,1,1,1,1,0

1,0,3,1,2,1,1,0,0,1,2,0,2,2,1,1,0

1,1,1,1,2,2,2,1,1,1,0,0,1,1,1,1,1

1,0,1,1,2,1,1,1,1,1,0,0,2,1,1,1,1

1,1,1,1,2,1,1,1,1,1,2,0,2,1,2,0,0

1,1,1,1,2,3,1,1,1,1,3,0,2,1,1,0,1

1,0,3,1,2,2,2,1,1,1,0,0,1,1,1,1,0

1,0,1,1,2,1,1,1,0,0,2,0,2,1,1,1,0

1,1,1,1,2,1,1,1,0,1,2,0,2,1,1,0,1

1,1,3,1,2,1,1,1,0,1,1,0,2,1,1,0,1

1,1,1,1,2,3,2,1,1,0,0,0,1,1,1,1,1

1,1,1,1,3,3,1,1,0,1,4,0,2,1,1,0,1

1,1,2,1,2,3,2,1,1,1,0,0,2,1,1,1,1

1,0,1,1,2,2,1,1,1,1,1,0,2,1,1,1,0

1,1,1,1,2,3,1,1,1,1,2,0,2,1,1,0,1

1,1,1,1,2,3,1,1,1,1,2,0,2,1,1,1,1

1,0,1,1,2,1,1,0,0,0,0,0,2,3,1,1,0

1,1,1,1,2,1,2,1,1,1,0,0,1,1,1,1,0

1,0,3,1,2,3,2,0,1,0,0,0,1,1,13,1,1

1,0,1,1,2,1,1,1,0,1,0,0,2,1,1,1,1

1,0,1,1,2,1,1,1,0,1,0,0,2,1,1,1,0

1,0,1,1,2,3,2,1,1,1,2,0,2,1,1,0,1

1,0,1,1,1,1,1,1,1,0,1,0,1,1,1,1,0

1,0,1,1,2,2,2,1,1,1,0,0,2,1,1,0,1

1,0,1,2,3,2,1,1,1,1,0,0,2,1,1,0,0

1,0,1,1,2,1,1,1,0,1,2,0,2,1,1,1,0

1,1,0,1,2,3,1,1,0,1,2,0,2,1,1,1,1

1,1,1,1,2,2,1,1,0,1,0,0,2,1,1,1,0

1,0,0,2,2,3,1,1,0,1,2,0,2,1,1,0,1

1,0,0,1,2,3,1,1,0,1,6,0,2,1,1,0,0

1,0,1,1,2,2,1,1,0,1,2,0,2,1,1,1,1

1,0,3,1,2,2,1,1,1,1,26,0,2,1,1,0,1

1,0,1,1,2,3,1,1,0,0,0,0,2,1,1,1,0

1,0,1,1,2,1,1,0,0,1,0,0,2,1,1,1,0

1,0,1,1,2,1,1,1,0,0,2,0,2,1,1,1,0

1,1,3,1,2,3,1,1,0,1,0,0,2,1,1,1,0

1,1,1,1,2,1,1,1,1,1,0,0,2,1,1,1,0

1,0,1,1,2,1,1,1,0,0,2,0,2,1,1,1,1

1,0,1,2,2,1,2,1,1,1,6,0,2,1,1,0,0

1,1,1,1,2,1,1,1,0,1,0,0,2,1,1,1,0

1,1,0,1,2,3,1,1,1,1,2,0,2,1,1,0,0

1,1,0,1,2,3,1,1,0,1,6,0,2,1,1,1,1

1,0,1,1,2,1,1,0,1,0,0,0,2,1,1,0,1

1,1,1,1,1,3,1,0,0,1,6,0,2,1,1,1,0

1,1,1,1,1,4,1,1,0,1,0,0,2,3,1,1,1

1,1,1,1,2,3,1,1,0,1,0,0,2,1,1,1,0

1,0,1,1,2,1,1,1,0,1,0,0,2,1,1,1,0

1,0,1,1,2,2,1,1,0,1,0,0,2,1,1,0,0

1,1,3,1,1,1,1,1,1,0,0,0,2,1,1,0,1

1,0,1,1,1,3,2,1,1,1,2,0,2,2,1,1,0

1,0,1,1,1,3,1,0,1,1,2,0,2,3,1,1,0

1,1,1,1,2,1,1,1,0,1,0,0,2,1,1,1,0

1,1,3,1,2,1,1,1,0,1,0,0,2,1,1,0,1

1,0,1,1,2,3,1,0,0,1,0,0,2,1,1,1,1

1,0,1,1,2,1,2,1,0,1,0,0,1,1,1,1,0

1,1,1,1,2,3,1,1,0,1,2,0,2,1,1,1,0

1,1,0,1,2,3,1,1,1,1,6,0,2,1,1,0,1

1,0,2,1,2,2,1,1,0,1,0,0,2,1,1,1,1

1,0,1,1,2,1,1,1,0,1,2,0,2,1,1,1,0

1,0,1,1,2,1,2,1,1,1,0,0,2,1,1,1,0

1,0,1,1,2,2,2,1,1,1,2,0,1,1,1,1,1

1,0,1,1,2,1,1,1,0,1,0,0,2,1,1,1,1

1,1,3,1,2,1,1,1,0,1,1,0,2,1,1,1,0

1,1,1,1,2,3,1,1,1,1,0,0,2,1,1,0,1

1,1,1,1,2,2,1,1,0,1,2,0,2,1,1,0,1

1,1,3,1,2,3,1,1,0,1,1,0,2,1,1,0,1

1,1,1,1,2,3,1,1,0,1,1,0,2,1,1,1,1

1,0,1,1,2,3,1,1,0,1,0,0,2,1,1,1,1

1,0,1,1,2,3,1,1,0,1,1,0,2,1,1,1,1

1,1,1,1,2,3,1,1,0,1,2,0,2,1,1,1,1

1,0,1,1,2,3,1,1,0,1,3,0,2,1,1,1,1

1,1,1,1,2,3,1,1,1,1,1,0,2,1,1,0,1

1,1,1,1,2,3,1,1,1,1,1,0,2,1,1,0,1

1,1,1,1,2,3,2,1,1,0,0,0,1,1,1,1,1

1,1,1,1,2,3,1,1,0,1,2,0,2,1,1,0,1

1,1,1,1,2,3,2,1,0,0,6,0,1,1,1,0,1

1,0,1,1,2,3,1,1,1,1,2,0,2,1,1,1,0

1,1,1,1,2,3,1,1,1,1,1,0,2,1,1,0,1

1,1,1,1,2,3,1,1,1,1,2,0,2,1,1,0,1

1,1,1,1,2,3,1,1,0,1,2,0,2,1,1,1,0

1,1,1,1,2,3,1,1,0,1,0,0,2,1,1,0,1

1,1,1,1,2,3,2,1,0,0,1,0,1,1,1,1,1

1,1,1,1,2,3,1,1,0,1,1,0,2,1,1,0,1

1,1,1,1,2,3,1,1,0,1,2,0,2,1,1,1,1

1,1,1,1,2,3,1,1,0,1,2,0,2,1,1,1,1

1,1,1,1,2,3,1,1,0,1,1,0,2,1,1,1,0

1,1,1,1,2,3,1,1,0,1,1,0,2,1,1,0,1

1,0,1,1,2,3,1,1,0,1,4,0,2,1,1,1,0

1,1,1,1,2,3,2,1,1,0,2,0,1,1,1,1,1

1,1,1,1,2,3,1,1,0,1,4,0,2,1,1,0,1

1,0,1,1,2,3,1,1,1,1,2,0,2,1,1,0,1

1,1,2,1,2,3,1,1,0,1,0,0,2,1,1,1,0

1,0,1,1,2,3,1,1,0,1,0,0,2,1,1,1,0

1,0,1,1,2,3,1,1,1,1,2,0,1,1,1,1,1

1,0,1,1,2,3,1,1,1,1,0,0,2,1,1,0,1

1,1,1,1,2,3,1,1,0,1,0,0,2,1,1,1,0

1,1,1,1,2,3,1,1,0,1,1,0,2,1,1,1,0

1,1,1,1,2,3,1,1,1,1,0,0,2,2,1,1,1

1,1,2,1,2,3,2,1,1,0,0,0,2,1,1,0,1

1,0,1,1,2,3,2,1,1,1,5,0,2,1,1,0,1

1,1,1,1,2,3,1,1,0,1,6,0,2,1,1,0,1

1,1,1,1,2,3,1,1,0,1,0,0,2,1,1,0,1

1,1,1,1,2,3,1,1,0,1,0,0,2,1,1,0,1

1,0,1,1,2,1,2,1,1,0,2,0,1,1,2,1,1

1,0,1,1,2,1,1,1,1,1,2,0,2,1,1,0,1

1,1,1,1,2,3,1,1,0,1,2,0,2,1,1,0,1

1,0,1,1,2,3,1,1,0,1,0,0,2,1,2,0,1

1,0,2,1,2,1,1,1,1,1,2,0,2,1,1,0,1

1,0,3,1,2,1,1,1,0,1,0,0,2,1,1,1,0

1,0,1,1,2,2,1,0,0,1,0,0,2,2,1,1,0

1,0,1,1,2,1,1,1,0,1,2,0,2,1,1,0,1

1,0,3,1,2,3,2,1,1,0,0,0,1,1,1,1,1

1,1,1,1,2,3,1,1,0,1,4,0,2,1,1,1,0

1,0,2,1,2,3,1,1,0,1,1,0,2,1,1,0,1

1,1,1,1,2,3,1,1,0,1,4,0,2,1,1,0,1

1,0,1,1,2,3,1,1,0,1,2,0,2,1,1,0,1

1,0,3,1,2,1,1,1,0,1,1,0,2,1,1,1,0

1,0,1,1,2,2,1,1,0,1,0,0,2,1,1,0,1

1,1,1,1,2,3,1,1,0,1,0,0,2,1,1,1,0

1,1,0,1,2,3,1,1,1,1,2,0,2,1,1,0,0

1,1,0,1,2,3,2,1,1,1,0,0,2,2,1,0,0

1,0,0,1,2,2,1,1,0,1,5,0,2,1,1,1,1

1,1,1,1,2,2,1,2,0,1,0,0,2,1,1,0,1

1,1,0,1,2,3,1,1,0,1,6,0,2,1,1,1,0

1,0,1,1,2,2,1,1,0,1,2,0,2,1,1,1,0

1,0,1,1,2,3,2,1,0,1,5,0,2,1,1,1,0

1,0,1,1,2,1,2,1,1,1,2,0,2,1,1,1,1

1,1,1,1,2,2,1,1,0,1,1,0,2,1,1,1,1

12,1,3,1,1,2,1,1,0,1,0,0,2,1,1,0,0

1,0,2,1,3,1,1,1,0,1,0,0,2,1,1,0,0

1,0,3,1,2,2,2,1,1,1,2,0,1,1,1,1,1

1,1,1,1,1,1,1,0,0,1,0,0,2,2,1,1,0

1,0,1,1,2,3,1,1,1,1,0,0,2,1,1,1,1

1,0,1,1,2,1,2,1,1,1,2,0,1,1,1,1,0

1,0,2,1,3,1,2,1,1,1,0,0,1,2,1,1,0

1,1,1,1,2,3,1,1,1,1,2,0,1,1,1,1,1

1,1,1,1,2,1,1,1,1,1,0,0,2,1,1,0,1

1,0,3,1,2,2,1,1,0,1,0,1,2,1,1,0,1

1,0,1,1,2,2,1,1,0,1,0,0,5,1,1,1,0

1,0,1,1,1,2,1,0,1,1,0,0,2,1,1,0,1

1,1,1,1,1,1,2,1,1,1,0,0,2,2,1,0,0

1,1,1,1,2,2,1,1,0,1,0,0,1,1,1,0,1

1,0,1,1,1,1,2,1,1,0,0,0,1,1,1,1,0

1,0,1,1,2,4,1,1,0,1,1,0,2,1,1,1,0

1,1,3,1,1,3,1,1,0,1,2,0,2,1,1,1,0

1,1,1,1,2,3,1,1,0,1,0,0,2,1,1,1,0

1,1,1,1,2,3,1,1,0,1,0,0,2,1,1,0,1

1,0,1,1,2,3,1,1,1,1,6,0,2,1,1,0,1

1,0,1,1,2,3,1,1,1,1,0,0,2,1,1,0,1

1,1,1,1,2,3,1,1,0,1,2,0,2,1,1,0,1

1,1,0,1,2,3,1,1,1,1,6,0,2,1,1,0,0

1,0,1,1,2,1,2,1,1,1,2,0,1,1,1,1,1

1,1,1,1,2,3,1,0,1,1,6,0,2,1,1,0,0

1,0,3,1,2,3,2,1,1,0,6,0,2,1,13,0,0

1,0,1,1,2,2,1,1,0,1,0,0,2,1,1,1,0

1,0,0,1,2,2,1,1,0,1,1,0,2,1,1,0,0

1,1,0,1,2,3,1,1,0,1,6,0,1,1,1,1,0

1,1,0,1,2,3,2,1,1,1,2,0,2,1,1,0,0

1,1,0,1,2,3,1,1,1,1,2,0,2,1,1,0,1

1,0,1,1,1,3,1,0,1,1,2,0,2,3,1,1,0

1,0,0,1,2,3,2,1,1,1,2,0,2,1,1,1,0

1,1,1,1,2,1,1,1,0,1,5,0,2,1,1,1,0

1,0,3,1,2,1,1,1,1,1,0,0,2,1,1,0,1

1,0,3,1,2,1,2,1,1,1,0,0,2,1,1,1,0

1,0,1,1,2,1,1,1,1,1,0,0,2,1,1,1,0

1,0,1,1,2,1,1,1,1,1,2,0,2,1,1,0,0

1,0,2,1,2,1,1,1,0,1,1,0,2,1,1,1,0

1,1,0,1,2,3,1,1,1,1,6,0,2,1,1,1,1

1,1,1,1,1,1,1,1,0,1,0,0,2,1,1,0,1

1,0,1,1,2,1,1,0,0,1,0,0,2,3,1,1,0

2,0,1,1,2,3,1,1,0,1,0,0,2,1,1,1,1

1,0,1,1,1,2,1,1,1,1,0,0,2,1,1,1,1

1,0,1,1,2,1,2,1,1,1,0,0,2,1,1,0,1

1,0,0,1,2,3,1,1,0,1,2,0,1,2,1,1,1

1,0,1,1,2,1,1,0,1,0,0,0,2,1,1,1,0

1,1,1,1,2,1,1,1,0,1,6,0,2,1,1,1,0

1,1,1,1,2,2,1,1,0,1,2,0,1,1,1,1,0

1,1,3,1,2,3,2,1,0,0,1,0,1,2,1,1,1

1,1,3,1,1,1,1,1,0,1,0,0,2,3,1,0,1

1,0,0,1,2,1,2,0,0,0,2,0,1,1,1,1,0

1,0,1,1,2,3,1,1,0,0,0,0,2,1,1,1,1

1,0,2,1,2,1,2,1,1,1,2,0,1,3,1,1,0

1,1,0,1,2,3,1,1,0,1,2,0,2,1,1,1,0

1,0,1,1,2,1,1,1,1,1,0,0,2,1,1,0,1

1,0,1,1,2,1,1,1,1,1,0,0,2,2,1,1,0

1,0,1,1,2,3,1,1,1,1,0,0,2,1,1,1,0

1,0,0,1,2,1,1,1,0,1,6,0,2,1,13,0,0

1,1,1,1,2,1,1,1,0,1,0,0,2,1,2,1,0

2,1,1,1,2,3,1,1,1,1,2,0,2,1,1,1,1

1,1,1,1,2,2,1,1,0,1,1,0,2,1,1,0,1

1,1,1,1,2,2,2,1,1,1,2,0,2,1,1,1,0

1,1,1,1,2,2,2,1,1,1,2,0,2,1,1,0,0

1,0,1,1,2,2,2,1,1,1,0,0,2,1,1,1,0

1,1,2,1,2,1,1,1,0,1,6,0,2,1,1,1,0

1,0,1,1,2,3,1,1,0,1,6,0,2,1,1,1,1

1,1,2,1,2,2,1,1,0,1,6,0,2,1,1,1,0

1,0,1,1,2,1,1,1,1,1,6,0,2,1,1,1,0

1,1,1,1,2,1,1,1,0,1,0,0,2,1,1,0,1

1,0,0,1,2,3,1,1,0,1,6,0,2,1,1,1,0

1,0,3,1,2,1,2,1,1,1,2,0,2,1,1,0,1

1,1,1,1,1,3,1,1,1,1,0,0,2,1,1,1,0

1,0,1,1,2,1,2,1,1,1,0,0,2,1,1,1,0

1,0,1,1,2,1,1,1,1,0,2,0,2,1,1,1,0

1,0,1,1,2,1,1,0,0,0,0,0,2,3,1,1,1

1,0,0,1,2,2,1,1,1,1,6,0,2,1,1,1,0

1,0,0,1,2,3,1,1,0,1,6,0,2,1,1,1,0

1,0,0,1,2,3,1,1,0,1,6,0,2,1,1,1,0

1,0,3,1,2,3,2,1,1,1,2,0,2,1,1,0,1

2,0,2,1,1,1,2,1,0,0,6,0,2,1,13,1,1

1,0,1,1,2,3,1,1,0,1,2,0,2,1,1,1,0

1,1,0,1,2,3,1,1,0,1,6,0,2,1,1,1,0

1,1,0,1,2,3,1,1,1,1,6,0,2,2,1,0,0

1,0,1,1,2,1,2,1,1,1,0,0,2,1,1,0,1

1,0,1,1,2,2,1,1,0,1,0,0,2,1,1,1,1

1,0,3,1,2,1,1,1,1,1,6,0,2,1,1,1,0

1,0,1,1,2,1,2,1,0,1,3,0,2,1,1,1,0

2,0,1,1,2,3,1,1,0,1,6,0,2,1,1,1,1

1,1,1,1,2,2,1,1,1,1,2,0,2,1,1,1,1

1,0,1,1,2,2,1,1,1,1,0,0,2,1,1,1,0

1,0,2,1,2,4,1,1,0,1,0,0,2,1,1,1,0

1,0,0,1,2,3,1,1,0,1,6,0,2,1,1,1,0

1,0,1,1,2,1,1,1,1,1,1,0,2,1,1,0,1

1,0,1,1,3,1,2,1,0,1,5,0,2,1,1,1,0

1,0,1,1,2,4,1,1,0,1,0,0,2,1,1,1,1

1,0,1,1,2,1,1,1,0,1,0,0,2,1,1,1,1

1,0,1,1,3,1,1,1,0,1,1,0,2,1,1,1,0

1,0,1,1,2,3,1,1,0,1,0,0,2,2,1,1,0

1,0,1,1,2,1,2,1,1,1,2,0,2,1,1,1,0

1,0,0,1,1,1,1,1,0,1,0,0,2,1,1,1,0

1,1,2,1,2,3,2,1,0,1,0,0,2,1,1,1,0

1,0,1,1,2,3,1,1,1,1,2,0,2,1,1,0,1

1,1,2,1,2,3,2,1,1,1,0,0,2,1,1,0,1

1,0,1,1,2,2,2,1,1,0,0,0,1,1,1,1,0

1,0,2,1,2,1,1,1,0,1,0,0,2,1,1,1,1

1,0,3,1,2,1,1,1,1,1,1,0,2,1,1,1,1

1,1,1,1,2,4,2,0,1,0,0,0,2,1,2,0,1

1,1,1,1,2,2,2,1,1,1,0,0,1,1,1,1,0

1,0,1,1,2,1,1,1,0,1,2,0,2,1,1,1,0

12,0,1,1,2,1,2,1,1,1,0,0,1,1,2,1,0

1,0,2,1,3,2,2,1,0,1,0,0,1,1,1,1,0

1,1,1,1,2,2,2,1,1,1,0,0,2,1,1,0,1

1,1,0,1,2,1,1,1,0,1,0,0,2,1,1,1,0

1,0,1,1,2,3,1,1,0,1,1,0,2,1,1,1,0

1,0,1,1,2,3,1,1,0,1,2,0,2,1,1,1,0

1,0,3,1,2,1,2,1,1,1,0,0,2,1,1,1,0

1,1,1,1,2,3,1,1,0,1,4,0,2,1,1,1,1

1,1,1,1,2,2,1,1,0,1,0,0,2,1,1,1,0

1,0,1,1,2,2,2,1,1,1,0,0,2,2,1,1,0

1,0,2,1,3,2,1,1,1,1,0,0,1,1,1,1,0

1,0,1,1,1,1,1,1,1,1,0,0,2,1,1,1,1

1,1,1,1,2,3,1,1,1,1,2,0,2,1,1,1,0

1,1,2,1,2,3,1,1,1,1,2,0,2,1,1,1,1

1,0,3,1,2,2,1,1,0,1,0,0,2,1,1,1,0

1,0,1,1,2,1,1,1,0,1,0,0,2,1,1,1,0

1,0,3,1,2,1,1,1,0,1,0,0,2,1,1,1,1

1,0,1,1,2,1,1,1,1,0,2,0,2,1,1,1,0

1,0,3,1,2,2,1,1,0,1,1,0,2,1,1,0,0

1,1,1,1,2,1,1,1,0,1,1,0,2,1,1,1,0

1,0,1,1,2,1,2,1,1,1,2,0,1,1,1,1,1

1,1,1,1,2,1,1,1,0,1,0,0,2,1,1,1,0

1,0,1,1,2,3,1,0,0,1,0,0,2,1,13,0,0

1,1,1,1,2,2,1,1,1,1,0,0,1,1,1,0,1

1,1,1,1,2,1,2,1,0,1,0,0,2,1,1,1,0

1,0,1,1,1,3,1,1,0,1,0,0,2,1,1,1,1

1,1,1,1,3,3,1,1,0,1,0,0,2,1,1,0,1

1,1,2,1,2,3,1,1,1,1,2,0,2,1,1,1,0

1,0,1,1,2,4,1,1,0,1,2,0,2,1,1,1,0

1,1,1,1,2,3,1,1,1,1,2,0,2,1,1,0,1

1,0,1,1,2,2,1,1,0,1,0,0,2,1,1,1,0

1,1,2,1,2,2,1,1,1,1,2,0,2,1,1,1,0

1,0,1,1,2,1,2,0,0,0,2,0,1,1,1,1,1

1,0,1,1,2,2,1,1,0,1,2,0,2,1,1,1,0

1,0,1,1,2,3,2,1,1,1,2,0,2,1,1,1,1

1,0,2,1,2,1,1,0,0,1,0,0,2,1,1,1,0

1,0,0,1,2,3,1,1,1,1,2,0,2,1,1,1,0

1,1,2,1,2,2,1,1,0,1,0,0,2,1,1,0,1

1,1,1,1,2,3,1,1,0,1,0,0,2,1,1,0,1

1,0,1,1,2,1,2,1,1,1,0,0,1,1,1,1,0

1,0,0,1,2,1,2,1,1,1,0,0,2,1,1,1,0

1,0,1,1,2,1,2,1,1,1,2,0,5,2,1,0,0

1,0,1,1,2,1,1,1,0,1,2,0,2,1,1,1,0

1,0,1,1,2,3,1,1,1,1,2,0,2,1,1,1,0

1,0,2,1,1,1,1,1,1,0,0,0,2,1,1,1,0

1,0,3,1,2,1,1,1,0,1,1,0,2,1,1,0,0

2,0,3,1,1,4,1,1,0,1,0,0,2,1,1,1,1

7,1,1,1,2,3,1,1,1,1,6,0,2,1,1,0,0

1,0,1,1,2,2,1,1,0,1,6,0,2,1,1,1,1

1,0,1,1,2,1,1,1,0,1,0,0,2,1,1,1,0

1,0,1,1,2,2,1,1,0,1,2,0,2,1,1,1,0

1,0,1,1,2,1,2,1,0,1,2,0,1,1,1,1,0

1,1,2,1,2,1,2,1,1,1,0,0,2,1,1,0,1

1,1,1,1,2,3,1,1,0,1,0,0,2,1,1,0,1

1,0,1,1,2,3,1,0,0,1,0,0,2,1,1,1,0

1,0,1,1,2,1,1,1,0,1,0,0,1,1,1,0,0

1,0,1,1,2,1,1,1,0,1,0,0,2,1,1,1,0

1,0,1,1,2,1,2,1,1,1,0,0,2,1,1,0,1

1,0,1,1,2,1,1,0,0,1,0,0,2,1,1,1,0

1,0,1,1,2,3,1,1,1,1,1,0,2,1,1,0,1

1,1,0,1,2,3,2,1,0,1,6,0,1,1,1,1,0

1,1,1,1,2,1,1,1,0,1,0,0,2,1,1,1,0

1,0,0,1,2,3,1,1,1,1,2,0,2,1,1,1,0

1,1,1,1,2,1,1,1,1,1,2,0,2,1,1,1,0

1,0,2,1,2,1,1,1,1,1,0,0,2,1,1,0,1

1,0,1,1,2,1,1,0,0,0,0,0,1,3,1,1,0

1,0,1,1,2,2,1,1,1,0,1,0,2,1,1,1,0

1,1,0,1,2,1,2,1,1,1,0,0,1,1,1,1,1

1,0,1,1,1,1,1,1,1,1,6,0,2,1,1,1,0

1,1,0,1,2,3,2,1,0,0,2,0,1,1,1,1,0

1,1,1,1,2,2,1,1,0,1,0,0,2,1,1,1,0

1,1,1,1,2,3,1,1,1,1,3,0,2,1,1,1,1

1,1,1,1,2,2,2,1,1,1,0,0,2,1,1,0,0

1,1,1,2,2,1,2,1,1,1,0,0,2,2,1,0,0

1,0,2,1,3,1,1,1,0,1,0,0,2,1,1,0,0

1,0,1,1,2,3,1,0,0,1,6,0,2,1,1,1,1

1,0,1,1,2,2,1,1,1,1,0,0,1,1,1,1,0

1,0,1,1,1,3,1,1,1,1,2,0,2,1,1,0,1

1,0,1,1,2,1,1,1,0,0,2,0,2,1,1,1,1

1,0,0,1,2,3,1,1,0,1,2,0,2,1,1,1,0

1,1,0,1,2,3,1,1,0,1,6,0,2,1,1,1,1

1,0,1,1,2,1,2,1,1,1,2,0,2,1,1,0,1

1,0,1,1,2,2,1,1,0,1,6,0,2,1,1,1,0

1,1,2,1,1,1,1,1,0,1,0,0,2,1,1,0,1

1,0,1,1,1,1,1,1,0,1,0,0,2,3,1,1,0

1,0,1,1,2,1,2,1,1,1,2,0,2,1,1,1,0

1,0,1,1,2,2,1,1,0,1,0,0,2,1,1,1,1

1,0,1,1,2,2,1,1,0,1,0,0,2,1,1,1,0

1,0,3,1,2,1,1,1,0,1,0,0,2,1,1,1,0

1,0,2,2,2,2,1,1,0,1,0,0,2,1,1,1,1

1,0,1,1,1,1,1,0,0,1,0,0,1,1,1,1,1

1,1,1,1,2,1,1,1,0,1,2,0,2,1,1,1,1

1,0,3,1,2,1,1,1,0,1,1,0,2,1,1,1,1

1,0,1,1,1,1,1,1,0,1,0,0,2,1,1,1,0

1,1,3,1,2,1,1,1,0,1,0,0,2,2,1,1,0

1,0,1,1,1,3,1,1,0,1,0,0,2,1,1,1,0

1,1,3,1,2,3,1,1,1,1,1,0,2,1,1,1,1

1,1,0,1,1,3,1,1,0,1,6,0,2,1,1,1,0

1,0,3,1,2,1,2,1,1,1,0,0,1,1,1,1,1

1,0,1,1,2,1,1,1,1,1,2,0,2,1,1,1,1

1,0,2,1,2,2,2,1,1,1,0,0,1,1,1,1,0

1,0,1,1,2,2,2,1,1,1,2,0,2,1,1,0,1

1,1,1,1,2,1,1,1,0,1,0,0,2,1,1,0,1

1,0,2,1,2,1,1,1,0,1,6,0,2,1,13,0,1

1,0,2,1,4,1,1,1,1,1,0,0,2,1,13,1,1

1,0,1,1,2,1,1,1,0,1,0,0,2,1,2,1,1

5,0,1,1,2,2,1,1,0,1,0,0,2,1,1,1,1

1,0,2,1,2,3,1,1,1,1,0,0,2,1,13,1,1

1,0,1,1,2,1,1,1,0,1,0,0,2,1,1,1,0

1,0,1,2,2,2,1,1,1,1,0,0,2,1,1,0,1

1,0,1,1,2,1,2,1,0,1,4,0,1,1,1,1,1

1,1,1,1,1,3,1,1,1,0,2,0,2,1,1,0,0

1,0,3,1,2,1,1,0,0,1,0,0,2,1,1,1,1

1,0,0,1,2,3,1,1,0,1,6,0,2,1,1,1,0

1,1,1,1,2,1,1,1,0,1,4,0,2,1,1,0,0

1,0,1,1,2,2,1,1,1,1,0,0,2,1,1,1,0

1,0,1,1,2,3,1,0,0,0,0,0,2,2,1,1,0

1,1,1,1,2,2,1,1,1,1,0,0,2,1,1,0,0

1,1,3,1,1,3,1,1,1,1,2,0,2,2,1,0,0

1,1,3,1,2,3,1,1,0,1,6,0,2,1,1,1,1

1,0,1,1,1,3,1,1,0,0,0,0,2,1,1,1,1

1,1,1,1,2,3,1,1,0,1,0,0,2,1,1,0,1

0,1,1,1,2,2,1,1,0,1,1,0,2,1,1,1,1

1,1,1,1,2,2,1,1,1,1,6,0,2,1,1,1,0

1,1,1,1,2,1,1,1,0,1,0,0,2,1,1,0,1

1,1,1,1,2,1,1,1,1,1,0,0,2,1,1,0,0

1,1,3,1,2,1,1,1,0,1,0,0,2,2,1,1,0

1,0,1,1,1,3,1,0,0,1,2,0,2,3,1,1,0

1,0,1,1,2,1,1,0,0,0,0,0,2,1,1,1,1

1,0,0,1,2,1,1,1,0,1,0,0,2,1,1,1,1

11,0,1,1,2,1,2,1,1,1,0,0,2,1,1,1,0

1,1,1,1,2,3,2,1,1,1,2,0,2,1,1,1,0

1,1,1,1,2,1,2,1,1,1,0,0,1,1,1,1,1

1,0,1,1,2,2,2,1,1,1,2,0,2,1,1,1,0

1,1,1,1,2,3,1,1,0,1,5,0,2,1,1,0,1

1,0,1,1,2,1,2,1,1,1,2,0,2,1,1,0,1

1,0,3,1,2,3,1,1,0,1,0,0,2,1,1,1,1

1,1,3,1,2,2,2,1,0,1,0,0,1,1,1,1,1

1,1,1,1,2,2,1,1,1,1,0,0,2,1,1,1,1

1,0,1,1,2,1,1,1,1,1,0,0,2,1,1,1,0

1,0,1,1,2,1,1,1,0,1,2,0,2,1,1,1,1

1,1,3,1,2,1,2,1,1,1,0,0,2,1,1,0,1

1,0,3,1,1,2,1,1,0,1,0,0,2,1,1,1,0

1,1,0,1,2,3,2,1,1,1,2,0,2,1,1,0,1

1,0,1,1,1,1,2,1,0,1,1,0,2,1,1,0,0

1,1,1,1,2,1,2,1,0,1,0,0,1,1,1,1,0

1,0,1,1,2,1,1,1,0,0,2,0,2,1,1,1,0

1,0,1,1,2,1,1,1,0,1,1,0,2,1,1,1,0

1,0,1,1,2,3,2,1,0,0,4,0,2,1,1,1,1

1,0,1,1,2,1,2,1,0,1,0,0,2,1,1,1,1

1,0,2,1,2,4,1,1,1,1,2,0,2,1,1,1,0

1,0,1,1,2,4,2,1,0,1,0,0,2,1,2,1,0

1,1,2,1,2,3,1,1,0,1,2,0,2,1,1,1,1

1,0,1,1,2,2,1,1,0,1,0,0,2,1,1,1,1

1,0,1,2,3,2,2,1,1,1,0,0,2,1,1,0,1

1,1,1,1,2,1,1,1,1,1,0,0,2,1,1,1,0

1,1,3,1,2,2,1,1,0,1,1,0,2,1,1,0,1

1,0,2,1,3,1,1,1,0,1,6,0,2,1,1,1,0

1,0,1,1,2,3,1,1,0,1,3,0,2,1,1,1,1

1,0,2,1,2,3,1,1,0,1,2,0,2,1,1,0,1

1,1,1,1,2,1,1,1,1,1,0,0,2,1,1,0,0

1,1,1,1,2,2,1,1,1,1,0,0,2,1,1,0,1

2,1,1,1,2,4,2,1,0,1,0,0,2,1,1,1,1

1,0,1,1,2,1,1,1,1,1,0,0,1,1,1,1,0

1,0,1,1,2,1,2,1,1,1,0,0,1,1,1,1,1

11,0,3,1,2,3,1,1,0,1,0,0,2,1,1,1,0

1,0,0,1,2,1,1,1,1,1,1,0,2,1,1,1,0

1,1,3,1,2,1,1,1,1,1,1,0,2,1,1,1,0

2,0,1,1,2,1,2,1,0,1,0,0,1,1,1,1,0

1,0,1,1,2,2,2,1,1,1,0,0,1,1,1,1,0

1,0,2,1,2,1,1,1,0,1,0,0,2,1,13,0,0

1,1,1,1,2,3,2,1,1,1,0,0,1,1,1,1,0

1,1,1,1,1,3,1,1,0,1,0,0,2,3,1,0,1

1,0,1,1,2,1,2,1,1,1,2,0,2,1,1,1,0

1,1,1,1,2,1,1,1,1,1,2,0,2,1,1,1,1

1,0,1,1,2,1,2,1,1,1,0,0,1,1,1,1,0

1,0,1,1,2,2,2,1,1,1,2,0,1,1,1,1,1

1,0,1,1,2,1,1,1,0,1,0,0,2,1,1,1,0

1,1,1,1,2,3,1,1,1,1,2,0,2,1,1,0,1

1,0,1,1,2,2,1,1,0,1,1,0,2,1,1,0,0

1,0,1,1,2,1,1,1,1,1,0,0,2,1,1,0,1

1,0,3,1,2,3,1,1,1,1,6,0,2,1,1,1,0

1,0,1,1,2,1,1,0,0,1,0,0,2,2,1,1,0

1,1,1,1,1,4,1,1,0,1,0,0,2,1,1,0,1

1,0,1,1,2,1,1,1,1,1,0,0,2,1,1,1,0

1,0,1,1,1,2,1,1,0,1,0,0,2,1,1,1,0

1,0,0,1,3,1,1,1,0,1,1,0,2,1,1,1,1

1,0,3,1,2,3,1,1,0,1,0,0,2,1,1,1,0

1,1,2,1,2,1,1,1,0,1,6,0,2,2,1,1,0

1,0,1,1,2,1,2,1,0,1,0,0,2,1,1,1,0

1,0,1,1,2,2,2,1,1,1,0,0,1,1,1,1,0

1,1,1,1,2,3,2,1,1,0,0,0,1,1,1,1,0

1,0,3,1,2,4,1,1,0,1,1,0,2,1,1,1,1

1,0,0,1,2,3,1,1,0,1,6,0,5,1,1,1,1

1,0,1,1,2,1,1,1,1,1,0,0,2,1,1,1,0

1,0,1,1,2,1,1,1,0,1,0,0,2,1,1,1,0

1,0,1,1,2,1,1,1,0,1,2,0,2,1,1,1,0

1,0,0,1,2,3,1,1,1,1,2,0,2,2,1,1,0

1,0,2,1,2,2,1,1,0,1,1,0,2,1,1,1,0

1,0,1,1,2,1,2,1,1,1,2,0,2,1,1,1,1

1,0,0,1,2,3,1,1,1,1,6,0,2,1,1,1,0

1,0,1,1,2,3,1,1,1,1,1,0,2,1,1,0,1

1,0,3,1,2,1,1,1,0,1,1,0,2,1,1,1,0

1,1,1,1,2,1,1,1,0,1,0,0,2,1,1,1,0

1,1,0,1,2,3,2,1,0,1,0,0,2,1,1,1,0

1,1,0,1,2,2,2,1,1,1,0,0,2,1,1,1,1

1,0,2,1,2,1,1,1,1,1,6,0,2,2,1,1,0

1,1,1,1,2,3,1,1,0,1,0,0,2,1,1,1,0

2,0,1,1,2,1,1,1,0,0,2,0,2,1,1,1,1

1,0,3,1,2,3,1,1,0,1,0,0,2,1,1,1,1

1,0,1,1,1,3,1,1,1,1,6,0,2,1,1,1,0

1,0,1,1,2,1,2,1,1,1,0,0,2,1,1,1,0

2,1,3,1,1,3,1,0,0,1,0,0,2,1,1,1,1

1,0,1,1,2,1,1,1,0,1,1,0,1,2,1,1,1

1,0,1,1,2,3,1,1,0,1,0,0,2,1,1,1,1

1,1,1,1,2,1,1,1,0,1,0,0,2,1,1,1,0

1,0,1,1,2,1,2,1,0,1,0,0,1,1,1,1,1

1,0,1,1,2,1,1,1,0,1,2,0,2,1,1,1,0

1,0,3,1,2,2,1,1,0,1,1,0,2,1,1,1,0

1,1,1,1,2,3,1,1,0,1,0,0,2,1,1,1,0

1,0,1,1,2,1,1,0,0,0,2,0,2,1,1,1,1

1,0,1,1,3,1,1,1,0,1,6,0,2,1,13,0,1

1,0,1,1,2,1,1,1,0,0,2,0,2,1,1,1,1

1,1,1,1,2,2,1,1,0,1,0,0,2,1,1,0,1

2,0,1,1,1,1,1,1,0,1,0,0,3,2,2,0,1

1,1,1,1,2,3,1,1,0,1,0,0,2,1,1,1,0

1,0,1,1,2,2,1,1,1,1,2,0,2,1,1,0,1

1,1,1,1,2,3,1,1,1,1,2,0,2,1,1,0,0

1,1,1,1,2,1,1,1,0,1,0,0,2,1,1,1,0

1,0,1,1,2,2,1,1,0,1,5,0,2,1,1,1,1

1,1,1,1,2,1,1,1,0,1,0,0,2,1,1,0,1

1,1,1,1,2,1,1,1,0,1,0,0,2,1,1,0,1

1,1,1,1,2,1,1,1,1,1,0,0,2,3,1,0,0

1,0,1,1,2,1,1,1,1,1,2,0,2,1,1,0,1

1,0,1,1,2,1,1,1,0,1,0,0,2,1,1,1,1

1,0,1,1,2,1,2,1,0,1,0,0,2,1,1,1,1

1,0,1,1,2,2,2,0,0,0,0,0,1,1,1,1,0

1,1,0,1,2,3,2,1,1,1,2,0,2,1,1,0,0

1,0,1,2,1,1,1,0,1,1,0,0,2,3,1,0,0

1,1,1,1,2,1,2,1,1,1,2,0,1,1,1,1,0

1,1,3,1,1,3,1,1,0,1,2,0,2,1,1,1,0

1,0,1,1,2,2,1,1,1,1,6,0,2,1,1,1,0

1,0,1,2,3,2,1,1,0,1,0,0,2,1,1,1,0

1,0,1,1,1,1,1,1,0,1,0,0,2,1,1,1,0

1,0,1,1,2,1,1,1,0,1,2,0,2,1,1,1,0

1,1,1,1,2,1,1,1,0,1,0,0,2,1,1,1,1

1,1,3,1,2,1,1,1,0,1,0,0,2,2,1,1,0

1,0,0,1,2,3,2,1,1,1,2,0,2,1,1,1,0

1,0,2,1,2,4,1,1,0,1,0,0,2,1,1,1,1

1,0,1,1,2,2,2,1,1,1,0,0,2,1,1,0,1

1,0,1,1,2,1,1,0,0,1,0,0,2,1,1,1,1

1,0,1,1,1,1,1,1,0,1,0,0,2,1,1,1,0

1,0,2,1,2,2,1,1,1,1,0,0,2,1,1,0,1

1,0,1,1,2,1,1,1,0,0,2,0,2,1,1,1,0

1,0,1,1,2,4,1,1,0,1,0,0,2,1,1,1,0

1,1,1,1,2,2,1,1,1,1,0,0,2,1,1,1,0

1,1,1,1,2,1,1,1,0,1,1,0,2,1,1,1,0

1,0,1,1,3,1,1,1,1,1,0,0,2,1,1,1,0

1,1,1,1,2,1,2,1,1,1,0,0,1,1,1,1,0

1,0,0,1,2,3,1,1,1,1,2,0,2,1,1,0,1

1,1,1,1,2,3,2,1,0,1,6,0,2,1,1,0,1

1,0,1,1,2,1,1,1,0,1,0,0,2,1,1,1,0

1,0,1,1,2,2,2,0,1,0,6,0,1,1,1,1,0

1,1,3,1,2,1,1,1,0,1,0,0,2,2,1,0,1

1,0,3,1,2,1,1,1,1,1,0,0,2,1,1,0,0

1,0,1,2,3,1,1,1,0,1,2,0,2,1,1,0,1

1,0,1,1,2,1,1,1,0,1,0,0,2,1,1,1,1

1,0,1,1,3,1,2,1,1,1,0,0,2,1,2,1,0

1,0,3,1,2,3,1,1,0,1,0,0,2,1,1,1,1

1,0,0,1,2,3,1,1,0,1,0,0,2,1,1,1,1

3,1,0,1,1,3,1,1,0,1,0,0,2,1,1,1,0

1,0,1,1,2,3,1,1,1,1,0,0,2,1,1,0,1

1,0,1,1,2,2,2,1,1,1,2,0,1,1,1,1,1

1,1,1,1,1,1,1,1,1,1,5,0,2,1,1,0,0

1,1,1,1,2,3,2,1,0,1,6,0,1,1,1,1,0

1,1,2,1,2,3,1,1,0,1,3,0,1,2,1,1,0

1,0,1,1,3,1,1,1,1,1,6,0,2,1,13,1,1

1,0,2,1,3,2,1,1,0,1,0,0,2,1,1,1,0

1,0,1,1,2,2,1,1,0,1,0,0,2,1,1,1,0

1,0,2,1,2,1,1,0,0,1,0,0,2,1,1,1,1

1,0,1,1,2,1,2,1,1,1,2,0,1,1,1,1,0

1,0,3,1,1,2,1,1,0,1,0,0,2,1,1,1,0

1,1,1,1,2,4,1,1,0,1,5,0,2,1,1,1,1

1,0,1,1,2,2,1,1,0,1,0,0,2,1,1,0,0

1,0,1,1,2,1,1,1,1,1,0,0,2,1,1,1,0

1,0,1,1,1,2,1,1,0,1,0,0,2,1,1,0,0

1,1,2,1,2,1,2,1,1,0,0,0,1,3,1,1,0

1,1,3,1,1,1,1,1,1,1,0,0,2,1,1,0,0

1,0,1,1,2,2,1,1,0,1,0,0,2,1,1,1,0

1,0,3,1,1,1,1,1,1,1,0,0,1,1,1,1,0

12,0,1,1,2,1,2,1,1,1,6,0,1,1,1,1,0

1,1,3,1,2,2,1,1,0,1,2,0,2,1,1,1,0

1,0,3,1,2,2,1,1,0,1,0,0,2,1,1,1,0

1,0,1,1,1,2,1,1,1,1,0,0,2,1,1,1,0

1,0,1,1,2,2,1,1,0,1,2,0,1,1,1,1,1

1,0,1,1,2,1,1,1,0,1,0,0,2,1,1,1,0

1,0,1,1,2,2,2,1,1,1,0,0,2,1,1,1,1

1,1,3,1,2,2,1,1,1,1,0,0,2,1,1,0,0

1,0,1,1,2,2,1,1,0,1,0,0,2,1,1,1,0

1,0,1,1,1,2,1,1,1,1,0,0,2,1,1,0,0

2,0,0,1,1,3,1,1,0,1,0,0,2,1,1,1,0

1,0,3,1,1,2,1,1,0,1,0,0,2,1,1,0,1

1,1,0,1,2,1,1,1,0,1,6,0,2,1,1,1,1

1,1,1,1,2,2,1,1,1,1,0,0,2,1,1,0,1

1,0,1,1,2,2,2,1,1,1,0,0,2,1,1,1,0

1,0,2,1,2,1,1,1,1,1,0,0,2,1,1,1,0

1,0,3,1,2,1,1,1,0,1,6,0,2,1,13,0,1

1,0,2,1,2,3,1,1,0,1,0,0,2,2,1,1,0

2,0,0,1,1,2,1,1,1,1,0,0,2,1,1,0,0

1,0,1,1,2,1,1,1,0,1,0,0,2,1,1,1,1

1,0,1,1,2,3,1,1,0,1,2,0,2,1,1,1,0

1,0,1,1,2,2,2,1,1,1,2,0,1,1,1,1,1

1,0,1,1,2,3,1,1,0,1,0,0,2,1,1,1,1

1,0,1,1,1,1,1,1,0,1,0,0,2,1,1,0,0

1,0,0,1,1,2,1,1,0,1,0,0,2,1,1,0,0

1,0,1,1,2,2,1,1,0,1,0,0,2,1,1,1,0

1,0,0,1,2,1,1,1,0,1,1,0,2,1,1,1,1

1,0,3,1,2,1,1,0,0,1,0,0,2,2,1,1,0

1,0,1,1,2,1,1,1,0,1,0,0,2,1,1,1,0

1,0,2,1,2,1,2,1,1,1,2,0,2,1,1,0,1

1,0,3,1,1,2,1,1,0,1,0,0,2,1,1,1,1

1,1,1,1,2,1,1,1,0,1,0,0,2,1,1,0,1

1,1,0,1,2,3,2,1,1,1,2,0,2,1,1,0,1

1,0,1,1,2,1,1,1,1,1,0,0,1,1,1,1,0

1,0,1,1,2,1,2,1,1,0,2,0,1,1,1,1,1

1,0,1,1,2,1,1,1,1,1,0,0,2,1,1,0,1

1,1,1,1,2,2,1,1,0,0,0,0,2,1,1,1,0

1,1,2,1,2,2,1,1,0,1,0,0,2,1,1,0,1

1,1,1,1,2,3,1,1,1,1,0,0,2,1,1,1,1

1,0,3,1,3,2,1,0,0,1,0,0,2,2,1,1,0

1,0,3,1,2,1,1,1,0,1,0,0,2,1,1,1,0

1,1,1,1,2,2,1,1,0,1,1,0,2,1,1,1,1

1,0,1,1,2,2,2,1,1,0,1,0,1,1,1,1,0

1,0,1,1,2,1,1,1,1,1,2,0,2,1,1,0,1

1,0,1,1,3,1,2,1,0,1,0,0,1,1,2,1,0

1,0,1,1,2,3,1,1,0,1,0,0,2,1,1,1,1

1,0,2,1,3,1,1,1,0,1,0,0,2,1,1,1,0

1,0,1,1,2,1,1,0,0,0,5,0,2,1,1,1,0

1,0,3,1,1,2,1,1,0,1,0,0,2,1,1,1,0

1,1,1,1,2,1,1,1,0,1,1,0,2,1,1,1,1

1,0,0,1,2,3,1,1,1,1,2,0,2,1,1,1,0

1,1,0,1,2,3,1,1,1,0,0,0,2,2,1,0,1

1,0,1,1,2,2,1,1,0,1,0,0,2,1,1,1,0

1,0,1,1,2,2,1,1,0,1,0,0,2,1,1,1,0

1,0,1,1,2,2,1,1,1,1,2,0,2,1,1,0,1

1,0,3,1,2,1,2,1,0,1,2,0,2,1,1,1,0

1,1,1,1,2,3,1,1,0,1,1,0,2,1,1,0,1

1,1,1,1,2,1,1,1,0,1,0,0,2,1,1,1,1

1,0,1,1,2,1,1,1,1,1,2,0,2,1,1,0,1

12,0,2,1,3,2,2,1,0,1,0,0,1,1,1,1,0

1,0,1,1,3,1,1,1,1,1,0,0,1,1,1,1,0

1,0,1,1,2,1,1,1,1,1,4,0,2,1,1,0,1

1,1,1,1,1,3,1,1,1,1,2,0,2,1,1,1,0

1,0,1,1,2,2,1,1,0,1,0,0,2,1,1,1,0

1,0,1,1,3,1,2,1,1,1,6,0,2,1,13,1,1

1,1,1,1,2,2,1,1,0,1,0,0,2,1,1,1,1

1,0,2,1,2,1,1,1,1,1,0,0,2,1,1,1,1

1,1,1,1,1,4,1,1,0,1,0,0,2,1,1,1,0

1,1,1,1,2,1,1,1,0,1,0,0,2,1,1,1,0

1,0,1,1,2,1,2,1,0,1,0,0,1,1,1,1,1

1,0,1,1,2,1,1,1,0,1,0,0,2,1,1,1,0

1,0,1,1,2,3,1,1,1,1,2,0,2,1,1,1,0

1,1,3,1,2,3,2,1,1,0,0,0,2,1,1,0,1

1,0,3,1,2,1,1,1,0,1,4,0,2,1,1,1,1

1,0,1,1,2,1,1,0,0,1,0,0,2,1,1,1,0

1,1,1,1,2,3,1,1,1,1,3,0,2,1,1,1,1

1,0,1,1,1,2,1,1,0,1,0,0,2,1,1,1,1

1,0,1,1,2,3,1,1,0,1,2,0,2,1,1,1,0

1,1,2,1,1,1,1,1,0,1,6,0,2,1,1,0,1

1,0,2,1,2,4,1,1,1,1,0,0,2,1,1,1,1

1,0,1,1,2,1,1,2,0,1,0,0,2,1,1,1,0

1,1,2,1,3,3,2,1,0,1,0,0,2,1,1,1,0

1,0,3,1,2,2,1,1,0,1,0,0,2,1,1,1,1

1,0,3,1,2,2,1,1,0,1,1,0,2,1,1,1,1

1,0,1,1,1,2,1,1,0,1,2,0,2,1,1,1,1

1,0,1,1,1,1,1,1,0,1,0,0,2,3,1,1,0

1,0,1,1,2,1,1,1,0,1,0,0,2,1,2,1,0

1,0,1,1,2,2,1,1,0,1,2,0,2,1,1,1,1

1,0,2,1,2,2,1,1,0,1,0,0,2,1,1,0,0

1,0,1,1,2,1,2,1,1,1,0,0,2,1,1,0,1

1,0,1,1,2,2,1,1,0,1,2,0,2,1,1,1,1

1,1,1,1,2,1,2,1,1,1,2,0,1,1,1,1,1

1,0,1,1,2,1,1,1,0,1,0,0,2,1,1,1,0

1,1,2,1,2,1,1,1,1,1,2,0,2,1,1,1,1

1,0,0,1,2,1,2,1,1,1,0,0,2,2,1,1,0

1,1,2,1,2,2,1,1,0,1,0,0,2,1,1,0,1

1,0,1,1,2,1,2,1,1,1,2,0,2,1,1,1,1

1,0,3,1,2,2,2,1,0,1,0,0,2,1,1,1,0

1,0,3,1,2,2,1,1,0,1,0,0,2,1,1,1,0

1,0,1,1,2,2,1,1,0,1,0,0,2,1,1,1,0

1,0,1,1,2,1,1,1,1,1,0,0,2,1,1,1,0

1,1,3,1,1,3,1,1,0,1,0,0,2,1,1,0,1

1,0,1,1,2,1,2,1,1,0,0,0,1,1,1,1,1

1,1,1,1,2,2,1,1,1,1,1,0,2,1,1,1,1

1,0,1,1,2,1,1,1,0,1,0,0,1,1,1,1,0

1,1,3,1,2,1,1,1,1,1,0,0,2,1,1,0,0

1,0,1,1,2,2,1,1,0,1,0,0,2,1,1,1,0

1,1,1,1,2,1,2,1,0,0,0,0,1,1,1,1,1

5,0,0,1,1,1,1,1,0,1,0,0,2,1,1,1,0

1,0,1,1,2,1,2,0,0,0,0,0,1,3,1,1,1

1,0,2,1,2,1,2,1,1,1,0,0,2,1,1,1,1

1,0,1,1,2,2,2,1,1,0,0,0,1,1,1,1,0

1,0,3,1,2,1,2,1,1,1,2,0,1,1,1,1,0

1,0,1,1,2,1,2,1,1,1,0,0,1,1,1,1,0

1,0,1,1,2,3,2,1,0,0,0,0,1,1,1,1,0

1,0,1,1,2,1,1,1,0,1,1,0,2,1,1,1,1

1,1,3,1,1,3,1,1,0,1,0,0,2,3,1,1,0

1,1,1,1,2,3,2,1,1,1,6,0,2,1,1,0,1

1,1,1,1,2,3,1,1,0,1,2,0,2,1,1,0,1

1,1,1,1,1,2,1,1,1,1,1,0,2,1,1,0,1

1,1,2,1,2,3,1,1,1,1,0,0,2,1,1,1,1

1,0,1,1,2,3,2,1,0,1,4,0,1,1,1,1,1

1,0,2,1,2,3,1,1,1,1,1,0,2,1,1,0,1

1,0,3,1,2,3,1,1,0,1,0,0,2,1,1,1,0

1,0,1,1,2,3,1,1,0,1,0,0,2,1,1,1,0

1,0,1,1,2,4,1,1,0,1,0,0,2,1,1,1,1

1,1,1,1,2,1,1,1,0,1,0,0,2,1,1,1,0

1,0,1,1,2,1,2,1,1,1,2,0,1,1,1,1,0

1,1,2,1,2,2,1,1,0,1,0,0,1,1,1,1,0

1,1,1,1,2,1,1,1,1,1,4,0,2,1,1,0,0

1,0,1,1,2,2,1,1,1,1,2,0,2,1,1,1,0

1,1,1,1,2,3,1,1,0,1,4,0,2,1,1,0,1

1,0,1,1,2,2,1,1,0,1,0,0,2,1,1,1,1

1,1,0,1,2,3,2,1,0,1,2,0,1,1,1,1,1

8,0,1,1,3,3,2,1,1,1,0,0,2,1,1,1,0

1,1,1,1,2,3,2,1,1,1,0,0,2,1,1,0,1

1,0,1,1,2,1,1,1,0,0,0,0,2,1,1,1,1

1,1,1,1,2,3,1,1,1,1,2,0,2,1,1,0,1

1,1,1,1,2,3,2,1,1,1,0,0,2,1,1,0,1

1,1,1,1,2,1,1,1,0,1,0,0,2,1,1,1,1

1,0,0,1,1,1,1,1,0,1,5,0,2,1,1,1,0

1,0,1,1,2,1,2,1,1,1,2,0,2,1,1,0,1

1,0,1,1,2,2,1,1,0,1,2,0,2,1,1,1,0

1,0,3,1,2,1,1,1,0,1,0,0,2,1,1,1,0

1,0,1,1,2,2,2,1,1,1,2,0,1,1,1,0,0

1,1,1,1,2,1,2,0,1,0,0,0,1,2,1,1,0

1,0,3,1,2,1,1,1,0,1,2,0,2,1,1,1,0

1,0,2,1,2,1,1,1,0,1,1,0,2,1,1,0,0

1,0,1,1,2,1,2,1,1,1,2,0,1,1,1,1,1

1,1,1,1,1,1,1,1,0,1,0,0,2,1,2,1,0

1,0,1,1,2,3,1,1,1,0,0,0,2,1,1,1,0

1,1,1,1,2,1,2,1,1,1,0,0,1,1,1,0,0

1,1,1,1,2,1,1,1,0,1,0,0,2,1,1,1,0

1,1,1,1,2,1,1,1,1,1,0,0,2,1,1,0,0

1,1,0,1,2,1,2,1,1,1,2,0,2,1,1,0,1

1,0,1,1,2,1,1,1,0,1,0,0,2,2,1,1,0

1,0,1,1,3,1,2,1,1,1,1,0,2,1,1,0,1

1,0,1,1,2,3,1,0,0,1,0,0,2,2,1,1,0

1,0,1,1,2,2,3,1,0,1,0,0,2,1,1,1,1

1,1,1,1,2,3,1,1,1,1,1,0,2,1,1,0,0

1,0,1,1,2,3,2,1,1,1,0,0,1,1,1,1,0

1,0,3,1,1,1,2,1,1,1,0,0,1,1,1,1,0

1,0,2,1,3,2,1,0,0,1,1,0,2,2,1,1,0

1,1,1,1,2,3,1,1,1,1,0,0,2,1,1,0,1

1,0,3,1,2,2,1,1,0,1,0,0,2,1,1,1,0

1,0,1,1,2,2,1,1,0,1,2,0,2,1,1,1,0

1,1,1,1,2,3,1,1,0,1,2,0,2,1,1,0,1

8,1,1,1,2,1,1,1,1,1,0,0,2,1,1,0,1

1,0,1,1,2,2,1,1,1,1,1,0,2,1,1,1,0

1,1,1,1,2,3,1,1,0,1,4,0,2,1,1,1,1

1,0,2,1,2,3,1,1,0,1,1,0,2,1,1,1,1

1,1,1,1,2,3,1,1,1,1,0,0,2,1,1,1,1

1,0,1,1,2,3,1,0,0,1,0,0,2,1,13,0,1

1,1,2,1,3,3,1,1,1,1,2,0,2,1,1,0,1

7,1,2,1,2,3,1,1,1,1,0,0,2,1,1,1,1

1,1,1,1,2,3,2,1,0,0,2,0,1,1,1,1,1

1,1,1,1,2,3,1,1,1,1,4,0,2,1,1,0,1

1,1,2,1,2,3,1,1,1,1,2,0,2,1,1,0,1

1,0,1,1,2,2,2,1,1,1,0,0,2,1,1,1,1

1,1,1,1,2,1,1,1,0,1,1,0,2,1,1,1,0

1,0,3,1,2,1,1,1,0,1,1,0,2,1,1,1,1

1,1,2,1,2,3,1,1,0,1,0,0,2,1,1,0,1

1,0,1,1,2,1,1,1,0,1,2,0,2,1,1,1,1

1,0,1,1,2,2,1,1,0,1,0,0,2,1,1,1,1

1,1,1,1,1,3,2,1,1,1,0,0,1,1,1,1,1

1,1,0,1,2,3,1,1,0,1,6,0,2,1,1,1,0

1,0,1,1,2,3,1,1,1,0,5,0,2,1,1,0,1

6,0,1,1,1,1,1,1,1,1,0,0,2,1,1,1,0

1,1,0,1,2,3,2,1,1,1,6,0,2,1,1,0,0

1,1,1,1,2,1,2,1,1,0,0,0,1,1,1,1,1

1,0,2,1,2,1,1,1,1,0,0,0,2,1,1,1,1

1,1,1,1,2,1,1,1,0,1,1,0,2,1,1,1,1

1,1,1,1,2,3,1,1,0,1,1,0,2,1,1,0,1

1,1,1,1,2,2,2,1,1,1,0,0,2,1,1,1,0

1,0,1,1,2,2,1,1,0,1,0,0,2,2,1,1,1

1,1,1,1,2,1,1,1,0,1,0,0,2,1,1,1,0

1,1,1,1,1,1,1,1,1,1,0,0,2,1,1,0,0

1,1,1,1,2,3,1,1,0,1,0,0,2,1,1,0,0

1,1,1,1,2,2,2,1,1,1,0,0,1,1,1,1,0

1,1,2,1,2,2,1,1,0,0,0,0,2,1,1,1,1

1,0,1,2,2,1,1,1,1,1,0,0,2,2,1,0,0

1,1,1,1,2,1,1,1,1,1,0,0,2,1,1,0,1

1,0,3,1,2,1,2,1,1,1,6,0,2,1,13,0,1

1,0,3,1,2,3,1,1,0,1,0,0,2,1,1,1,1

1,1,1,1,2,3,1,1,1,1,0,0,2,1,1,0,0

1,0,3,1,1,1,1,1,0,1,0,0,2,1,1,0,0

1,1,0,1,2,3,2,1,1,1,2,0,2,1,1,0,1

1,0,0,1,3,2,1,1,0,1,0,0,2,1,1,0,0

1,1,1,1,2,1,1,1,1,1,6,0,2,1,1,1,1

1,1,1,1,2,2,1,1,1,1,0,0,2,1,1,0,1

1,1,1,1,2,3,2,1,0,1,0,0,2,1,1,1,0

1,0,1,1,2,1,1,1,0,1,0,0,2,1,1,1,1

1,0,1,1,2,1,1,1,1,1,1,0,2,1,1,1,0

1,0,1,2,2,2,4,1,0,1,0,0,2,1,1,0,0

2,0,1,1,2,1,2,1,0,1,0,0,2,1,1,1,1

1,1,1,1,2,1,2,1,1,1,2,0,2,1,1,0,1

1,0,0,1,2,1,1,1,0,0,2,0,2,1,1,1,0

1,1,2,1,2,3,1,1,1,1,0,0,2,1,1,0,1

1,0,1,1,2,3,1,1,1,1,4,0,2,1,1,0,1

1,0,3,1,2,1,1,1,0,1,1,0,2,1,1,1,0

1,1,1,1,1,1,1,1,1,1,0,0,2,1,1,0,1

1,1,1,1,2,2,1,1,0,1,2,0,2,1,1,1,0

1,1,0,1,2,3,1,1,0,1,2,0,2,1,1,1,0

1,1,0,1,2,3,1,1,1,1,2,0,2,1,1,0,0

1,0,1,1,2,1,1,1,1,1,0,0,2,1,1,0,1

1,0,1,1,1,3,1,1,1,1,6,0,2,1,1,1,0

1,0,1,1,1,3,1,1,0,1,6,0,2,1,1,1,1

1,0,0,1,1,2,1,1,0,1,0,0,2,1,1,1,0

1,0,1,2,2,2,1,1,0,1,0,0,2,1,1,1,1

1,0,1,1,1,2,1,1,1,1,0,0,2,1,2,1,1

1,0,0,3,1,2,1,1,1,1,5,0,2,1,1,0,0

1,0,0,1,2,3,1,1,1,1,2,0,2,1,1,1,1

1,0,1,1,2,1,2,1,0,0,0,0,1,1,1,1,0

1,0,1,1,2,2,2,1,0,1,2,0,2,1,1,1,1

1,0,0,1,2,1,1,1,0,1,6,0,2,1,1,1,1

1,0,3,1,2,1,1,1,0,1,1,0,2,1,1,1,1

1,0,1,1,2,2,1,0,0,1,0,0,2,2,1,1,0

1,1,1,1,2,3,1,1,1,1,0,0,2,1,1,1,1

1,0,3,1,2,1,1,1,1,1,6,0,2,1,1,1,0

1,1,3,1,2,1,1,1,0,1,0,0,2,1,1,1,1

1,0,1,1,2,4,1,1,1,1,5,0,2,1,1,0,0

1,1,1,1,1,3,1,1,1,1,6,0,2,1,1,0,0

1,0,1,1,2,1,1,1,0,1,0,0,2,1,1,0,1

1,0,3,1,2,1,2,1,1,1,1,0,1,2,1,1,0

1,0,1,1,2,3,1,1,1,1,1,0,2,2,1,0,1

1,1,1,1,2,3,1,1,0,1,2,0,2,1,1,0,1

1,0,3,1,2,3,1,1,0,1,1,0,2,1,1,1,1

1,1,1,1,3,3,1,1,1,1,4,0,2,1,1,0,1

1,0,1,1,2,3,1,1,0,1,0,0,2,1,1,1,1

1,1,1,1,2,2,1,1,0,1,0,0,2,1,1,1,0

1,1,3,1,2,1,1,1,0,1,0,0,2,1,1,1,0

1,1,3,1,2,1,1,1,1,1,6,0,2,1,1,0,1

1,0,0,1,2,3,1,1,1,1,2,0,2,1,1,0,1

1,1,0,1,2,3,1,1,0,1,6,0,1,1,1,0,1

1,1,1,1,1,3,2,0,1,0,2,0,2,3,1,1,0

1,0,0,1,2,3,1,1,1,1,2,0,2,2,1,1,0

1,0,0,1,2,3,2,1,0,0,6,0,1,1,1,1,0

1,0,1,1,2,3,1,1,0,1,0,0,2,2,1,1,0

1,0,1,1,2,1,1,1,0,1,1,0,2,1,1,0,1

1,0,1,1,2,2,1,1,0,1,1,0,2,1,1,1,0

1,0,1,1,2,2,1,1,0,1,2,0,2,1,1,1,0

1,0,1,1,2,1,2,1,1,1,2,0,2,1,1,0,1

1,0,1,1,2,1,1,1,1,1,0,0,2,1,1,1,0

11,1,1,1,2,3,1,1,1,1,0,0,2,1,1,1,1

1,0,3,1,1,3,1,0,0,1,2,0,2,3,1,1,0

1,1,1,1,2,1,1,1,1,1,0,0,2,1,1,0,0

1,0,1,1,2,3,1,1,1,1,0,0,2,1,1,1,0

1,1,1,1,2,3,1,1,0,1,2,0,2,1,1,0,1

1,1,1,1,2,3,1,1,0,1,1,0,2,1,1,1,1

1,1,1,1,1,3,1,1,0,1,2,0,2,1,1,1,0

1,0,1,1,2,2,1,1,0,1,0,0,2,2,1,1,1

1,0,3,1,2,1,1,1,1,1,1,0,2,1,1,1,0

1,1,3,1,2,4,1,1,0,1,2,0,2,1,1,0,1

1,1,1,1,2,3,1,1,1,1,2,0,2,1,1,1,1

1,1,1,1,2,3,1,1,0,1,1,0,2,1,1,0,1

1,1,3,1,2,1,1,1,0,1,0,0,2,1,1,1,1

1,0,1,1,2,1,1,1,0,1,2,0,2,1,1,1,0

1,0,1,1,2,1,2,1,1,0,0,0,2,2,1,1,0

1,0,2,1,2,3,1,1,0,1,0,0,2,1,13,0,0

1,0,3,1,2,1,2,1,0,1,6,0,2,1,13,1,0

1,1,1,1,2,3,1,1,0,1,4,0,2,1,1,0,1

1,0,1,1,2,1,2,1,1,1,0,0,2,1,1,0,1

1,1,1,1,2,3,1,1,0,1,0,0,2,1,1,0,1

1,0,1,1,2,2,2,1,1,1,0,0,1,1,1,1,1

1,0,3,1,2,3,1,1,0,1,0,0,2,1,1,0,0

1,0,1,1,2,1,1,1,0,1,0,0,2,1,1,1,0

1,1,1,1,2,1,1,1,1,1,0,0,2,1,1,0,0

1,0,1,1,2,1,1,1,1,1,0,0,2,2,2,0,1

1,0,2,1,2,1,1,0,0,1,0,0,2,1,1,1,0

1,0,3,1,2,1,1,1,0,1,0,0,2,1,1,0,1

1,0,0,1,3,3,2,1,1,1,6,0,2,1,1,1,0

1,1,3,1,1,3,1,1,1,1,2,0,2,1,1,0,0

1,0,0,1,2,3,1,1,1,1,2,0,2,1,1,1,0

1,1,1,1,2,3,1,1,0,1,1,0,2,1,1,1,0

1,0,3,1,1,3,3,0,0,0,0,0,2,1,1,0,0

1,0,0,1,1,2,1,1,0,1,0,0,2,1,1,0,0

1,0,2,1,2,1,2,1,1,1,2,0,2,1,1,1,0

1,1,1,2,2,1,1,3,0,1,0,0,2,1,1,1,1

1,0,1,1,2,3,1,1,1,1,4,0,2,1,1,1,1

1,1,1,1,2,1,1,1,0,1,0,0,2,1,1,1,0

1,0,2,2,3,1,1,0,0,1,4,0,2,1,1,0,1

1,0,1,1,2,1,1,1,0,1,0,0,2,1,1,1,0

1,0,1,1,2,1,1,1,0,1,1,0,2,1,1,0,0

12,0,1,1,2,2,1,1,0,1,0,0,2,1,1,1,0

1,0,1,1,3,1,2,1,0,1,0,0,1,1,1,1,0

1,0,2,1,3,1,1,1,0,1,5,0,2,1,1,1,0

1,1,1,1,2,3,1,1,1,1,2,0,2,1,1,1,0

1,0,1,1,2,1,1,0,0,1,0,0,2,1,1,1,0

1,0,3,1,2,4,1,1,1,1,0,0,2,1,2,1,1

1,0,1,1,2,3,1,1,1,1,6,0,2,1,1,0,1

1,0,1,1,2,1,1,1,0,1,6,0,2,1,1,1,1

1,0,2,1,2,1,1,1,0,1,2,0,2,1,1,1,0

1,0,1,1,2,2,1,1,0,1,0,0,2,1,1,1,0

1,0,1,1,2,1,1,1,0,1,1,0,2,1,1,1,0

1,1,3,1,2,1,2,1,1,1,5,0,2,1,1,1,1

1,1,1,1,2,3,1,1,0,1,1,0,2,1,1,1,1

1,0,1,1,1,3,1,1,1,1,2,0,2,2,1,1,0

1,1,0,1,2,3,1,1,1,1,2,0,2,1,1,0,0

1,0,1,1,1,3,1,1,0,1,6,0,2,1,1,1,0

1,0,1,1,2,2,1,1,0,1,0,0,2,1,1,1,1

1,0,1,1,2,1,1,1,1,1,0,0,2,1,1,0,1

1,1,1,1,2,1,1,1,0,1,0,0,2,1,1,1,0

1,1,1,1,2,3,1,1,0,1,0,0,2,1,1,1,1

1,0,1,1,2,1,1,1,1,0,2,1,2,1,1,0,0

1,1,1,1,2,2,1,1,0,1,1,0,2,1,1,1,1

1,0,1,1,2,2,2,1,1,0,1,0,1,1,1,1,0

1,0,1,1,2,1,1,1,0,1,1,0,2,1,1,1,1

1,0,1,1,2,1,1,1,0,1,0,0,2,1,13,0,1

1,0,2,1,2,3,1,1,1,1,4,0,2,1,1,0,1

1,1,1,1,2,3,1,1,1,1,0,0,2,1,1,0,1

1,1,1,1,2,3,1,1,0,1,1,0,2,1,1,0,1

1,1,1,1,2,3,2,1,0,1,1,0,1,1,1,1,1

1,0,1,1,2,3,1,1,1,0,0,0,2,1,1,0,1

1,0,1,1,2,1,2,1,1,1,0,0,2,1,1,1,1

2,1,1,1,2,2,1,1,1,1,0,0,2,1,1,0,1

1,0,1,1,1,2,1,1,0,1,0,0,2,1,1,1,0

1,0,2,2,2,2,1,1,0,1,0,0,2,1,1,0,0

1,0,3,1,1,1,1,1,1,1,0,0,2,1,1,1,0

1,0,1,1,1,1,1,1,1,1,0,0,2,1,1,1,0

1,1,2,1,2,1,2,1,0,1,0,0,2,2,1,0,1

1,1,3,1,2,1,2,1,1,1,0,0,2,1,1,0,1

1,0,1,1,2,1,1,1,0,1,0,0,2,1,2,1,1

1,0,1,1,3,1,1,1,0,1,1,0,2,1,1,0,0

1,0,1,1,1,1,1,1,0,1,0,0,2,1,1,1,1

1,0,1,1,2,3,1,1,0,1,0,0,2,1,1,1,1

1,0,1,1,2,3,1,1,0,1,2,0,2,1,1,1,0

1,0,1,1,2,3,1,1,0,1,2,0,2,1,1,1,0

1,1,1,1,2,2,2,1,1,1,0,0,2,1,1,1,0

1,1,1,1,3,3,1,1,0,1,4,0,2,1,1,0,1

1,0,1,1,2,3,1,1,0,1,5,0,2,1,1,1,1

1,0,1,1,2,3,1,1,0,1,0,0,2,1,1,1,0

1,0,1,1,1,3,2,0,0,1,2,0,1,3,1,1,0

1,0,1,1,2,2,1,1,0,1,0,0,2,1,1,1,1

1,0,2,2,3,3,1,1,0,1,0,0,2,1,1,1,1

1,0,2,1,3,2,2,0,0,1,0,0,1,1,1,1,0

1,1,1,1,2,1,2,1,1,1,2,0,2,1,1,0,1

1,1,1,1,1,1,1,0,1,1,6,0,2,2,1,1,0

1,0,1,1,2,2,2,1,1,1,0,0,1,1,1,1,0

1,0,1,1,2,1,1,1,0,1,0,0,2,1,1,1,0

1,0,1,1,2,2,2,1,1,1,2,0,2,1,1,1,1

1,0,1,1,2,2,1,1,0,1,0,0,2,1,1,1,0

1,1,2,1,2,2,2,1,1,1,0,0,2,1,1,1,1

1,0,1,1,1,3,1,1,0,1,0,0,2,1,1,1,0

1,1,1,1,2,3,2,1,0,0,0,0,1,1,1,1,1

1,1,1,1,2,1,1,1,1,1,0,0,2,1,1,1,1

1,1,1,1,2,3,1,1,1,1,2,0,2,1,1,0,0

1,0,1,1,1,1,1,1,0,1,0,0,2,1,1,1,1

1,0,1,1,2,2,2,1,1,1,2,0,1,1,1,1,0

1,0,1,1,2,2,2,1,1,1,2,0,2,1,1,0,1

1,1,3,1,2,1,1,1,0,1,0,0,2,1,1,1,0

1,0,1,1,2,2,1,0,1,1,0,0,2,2,1,1,0

1,0,3,1,2,3,2,1,1,0,0,0,1,2,2,1,1

1,0,1,1,1,1,1,1,0,1,0,0,2,1,1,1,0

1,0,1,1,2,1,2,1,0,0,6,0,2,1,1,1,1

1,0,1,1,2,1,1,1,1,1,0,0,2,1,1,1,0

1,0,2,1,3,1,1,0,0,1,0,0,2,2,1,1,0

1,0,1,1,2,1,1,0,1,0,0,0,2,1,1,0,1

1,1,1,1,2,2,1,1,0,1,2,0,2,1,1,0,1

1,1,3,1,1,3,1,1,0,1,0,0,2,1,1,1,0

1,0,3,1,2,1,1,1,0,1,2,0,2,1,1,1,0

1,0,1,1,2,1,1,1,0,1,0,0,2,1,1,0,1

1,0,1,1,3,2,1,1,1,1,0,0,2,1,1,1,1

1,0,1,1,2,2,1,1,0,1,2,1,2,1,1,0,1

1,1,1,1,2,3,1,1,1,1,2,0,2,1,1,1,1

1,0,1,1,2,1,1,1,0,1,1,0,2,1,1,0,0

1,0,1,1,2,2,2,1,1,1,2,0,1,1,1,1,0

1,0,1,1,2,2,2,1,1,1,2,0,2,1,1,1,0

1,1,2,1,2,2,1,1,1,1,0,0,2,1,1,0,0

1,0,1,1,2,3,1,1,0,1,0,0,2,1,1,1,1

1,1,1,1,2,3,1,1,0,1,0,0,2,1,1,1,0

1,0,1,1,1,1,1,0,0,1,0,0,2,1,1,1,0

7,0,2,1,2,1,1,0,1,1,0,0,2,1,1,1,0

1,0,1,1,2,1,1,1,0,1,1,0,2,1,1,1,0

1,0,1,1,2,3,1,1,0,1,4,0,2,1,1,1,1

1,1,1,1,2,2,1,1,0,1,1,0,2,1,1,0,1

1,0,1,1,2,1,1,1,0,1,0,0,2,1,1,0,0

1,1,1,1,2,1,1,1,1,1,0,0,2,1,1,1,1

1,1,1,1,1,3,1,1,0,1,0,0,2,1,1,1,0

1,0,1,1,2,2,2,1,1,1,2,0,1,1,1,1,1

1,0,1,1,2,1,1,1,1,0,0,0,1,1,1,1,1

1,1,0,1,2,3,2,1,0,1,6,0,2,1,1,1,0

1,0,1,1,1,2,1,1,1,1,0,0,5,1,1,0,0

1,1,3,1,2,1,2,1,1,1,0,0,2,1,1,1,1

1,0,3,1,2,1,2,1,1,1,0,0,1,1,1,1,1

1,0,1,1,2,1,2,1,0,0,0,0,2,1,1,1,1

1,1,0,1,2,3,2,1,0,1,6,0,1,2,1,1,1

1,0,1,1,2,1,1,1,0,1,0,0,2,1,1,1,1

1,0,1,1,2,4,1,1,0,1,0,0,2,1,1,1,1

1,0,1,1,3,1,1,1,1,1,2,0,2,1,1,1,1

1,0,1,1,2,1,2,1,1,1,2,0,2,1,1,1,0

1,0,1,1,2,1,1,1,0,1,0,0,2,1,1,1,1

1,0,3,1,1,1,1,0,0,1,0,0,2,3,1,1,0

1,1,1,1,1,1,1,1,1,1,0,0,2,1,1,0,1

1,0,1,1,2,3,1,1,0,1,1,0,2,1,1,1,1

1,0,3,1,2,3,1,0,0,1,6,0,2,1,13,1,0

12,0,0,1,2,1,1,1,0,1,6,0,2,1,13,1,1

1,0,1,1,2,1,1,1,0,1,0,0,2,1,1,1,0

1,0,2,1,2,1,1,1,0,1,0,0,2,2,1,1,0

1,0,3,1,2,1,1,1,1,1,1,0,2,1,1,0,1

1,1,0,2,3,3,1,1,1,1,0,0,2,1,1,1,1

1,0,1,1,2,1,1,0,0,0,0,0,2,3,1,1,1

1,0,1,1,2,1,1,1,0,1,0,0,1,1,1,1,0

2,0,1,1,2,1,1,1,0,1,0,0,2,2,1,0,0

1,0,0,1,2,3,1,1,1,1,2,0,5,1,1,0,1

1,0,1,1,2,1,2,1,0,1,0,0,1,1,1,1,1

1,0,1,1,2,1,1,1,1,1,0,0,2,1,1,0,1

1,1,1,1,2,1,1,1,1,1,0,0,2,1,1,0,0

1,0,0,1,1,2,1,1,1,1,0,0,2,1,1,1,0

1,0,1,1,2,1,1,1,0,1,0,0,2,1,1,1,0

1,0,1,1,2,2,2,1,1,1,2,0,1,1,1,1,0

1,0,2,1,3,3,2,1,1,1,0,0,1,1,1,1,0

1,0,0,1,2,2,1,1,0,1,0,0,2,1,2,1,0

1,0,3,1,2,1,1,1,0,1,0,0,2,2,1,1,1

1,0,3,1,2,2,1,1,0,1,0,0,2,2,1,1,1

1,0,1,1,2,1,2,1,1,1,0,0,1,1,1,1,1

1,1,1,1,2,1,2,1,1,1,0,0,2,1,1,0,0

1,0,1,1,2,1,1,1,0,1,0,0,2,1,1,1,1

1,1,1,1,2,1,1,1,0,1,2,0,2,1,1,1,1

1,0,1,1,2,1,1,1,0,1,0,0,2,1,1,1,1

1,0,1,1,2,1,2,1,1,1,0,0,2,1,1,1,0

1,1,1,1,1,3,2,1,1,1,2,0,2,1,1,0,0

1,0,1,1,2,2,1,0,0,1,6,0,2,2,1,1,0

1,0,3,1,2,2,1,1,0,1,0,0,2,1,1,1,1

1,0,0,1,2,3,1,1,1,1,2,0,2,1,1,0,1

1,1,1,1,2,1,2,1,1,0,0,0,1,1,1,1,1

1,1,3,1,1,3,2,1,1,0,1,0,2,1,1,0,1

1,1,1,1,2,3,1,1,0,1,0,0,2,1,1,1,1

1,0,1,1,2,1,1,1,0,1,2,0,2,1,1,1,0

1,0,0,1,2,3,1,1,0,1,2,0,2,1,1,1,0

1,1,0,1,2,3,1,1,0,1,6,0,2,2,1,1,0

1,0,1,1,2,1,1,1,1,1,0,0,2,1,1,1,1

1,0,1,1,2,1,1,0,0,1,0,0,2,1,1,1,0

1,0,3,1,1,1,1,1,0,1,0,0,2,1,1,1,1

1,1,1,1,2,3,1,1,0,1,0,0,2,1,1,0,1

1,1,1,1,2,2,1,1,1,1,0,0,2,1,1,1,0

1,0,0,1,2,3,1,1,1,1,2,0,2,1,1,1,0

1,0,0,1,2,1,1,1,0,1,0,0,2,1,1,1,0

1,0,1,1,1,2,1,1,0,1,0,0,2,1,1,1,0

1,0,2,1,2,2,1,1,0,1,1,0,2,1,1,1,0

1,0,3,1,2,1,1,1,0,1,0,0,2,1,13,0,1

1,0,1,1,2,2,1,1,1,1,5,0,2,1,1,1,0

1,0,0,2,3,3,1,1,1,1,6,0,2,1,1,1,0

1,1,1,1,2,1,1,1,1,1,0,0,2,1,1,0,1

1,0,1,1,2,1,1,0,0,1,0,0,2,2,1,1,0

1,0,1,1,2,1,2,1,1,1,2,0,1,1,1,1,1

1,0,1,1,3,1,1,1,0,1,0,0,2,1,1,0,1

1,1,1,1,2,1,1,1,0,1,1,0,2,1,1,0,1

1,1,1,1,1,1,1,1,0,1,4,0,2,1,1,1,0

1,0,0,1,2,1,1,1,0,1,0,0,2,1,1,1,0

1,0,1,1,2,2,1,1,0,1,0,0,2,1,1,1,1

1,1,1,1,2,4,1,1,0,1,2,0,2,1,1,1,0

1,0,1,1,2,1,1,1,0,1,1,0,2,1,1,1,0

1,1,1,1,2,3,1,1,0,1,4,0,2,1,1,1,0

1,1,1,1,2,1,1,1,1,1,2,0,2,1,1,1,0

1,1,3,1,2,1,1,1,0,1,0,0,2,1,1,0,1

1,0,1,1,2,1,2,1,1,0,6,0,1,1,13,1,1

1,0,3,1,2,2,1,1,0,1,1,0,2,1,1,1,0

1,0,1,1,2,1,2,1,1,1,0,0,1,1,1,1,0

1,0,3,1,2,2,1,1,1,1,0,0,2,1,1,0,0

1,0,1,1,2,1,1,0,1,1,0,0,2,3,1,1,0

1,0,3,1,2,1,1,1,0,1,0,0,2,1,1,1,1

1,1,1,1,2,3,1,1,1,1,1,0,2,1,1,1,1

1,0,1,1,2,1,1,1,0,1,0,1,2,1,1,0,0

1,0,1,1,2,1,1,1,1,1,0,0,2,1,1,0,1

1,1,1,1,2,3,1,1,1,1,0,0,2,1,1,1,1

1,0,1,1,2,3,1,1,0,1,0,0,2,1,1,1,1

1,1,1,1,2,3,1,1,0,1,0,0,2,1,1,1,1

1,0,1,1,2,3,1,1,0,1,6,0,2,1,1,1,1

1,0,1,1,2,2,1,1,0,1,0,0,2,1,1,1,0

2,1,1,1,1,1,1,1,0,1,0,0,2,3,1,1,0

1,0,1,1,2,1,1,1,0,1,2,0,2,1,1,1,1

1,1,1,1,2,3,1,1,1,0,0,0,2,1,1,0,0

1,0,0,1,2,1,1,1,1,1,6,0,2,1,1,1,1

1,0,1,1,2,1,2,0,1,0,0,0,2,1,1,1,1

1,1,1,1,1,1,1,1,0,0,0,0,2,1,1,0,1

1,0,1,1,2,1,1,1,0,1,0,0,2,1,1,1,1

1,1,1,1,2,1,1,1,1,1,0,0,2,1,1,0,1

1,0,1,2,3,2,1,1,1,1,0,0,2,1,1,0,0

1,0,1,1,2,1,1,0,0,0,5,0,2,1,1,1,1

1,0,3,1,2,1,1,1,0,1,6,0,2,1,1,1,1

1,1,1,1,2,3,2,1,0,1,3,0,2,1,1,1,1

1,1,0,1,1,1,1,1,0,1,0,0,2,1,1,1,1

1,1,1,1,2,3,1,1,1,1,2,0,2,1,1,1,1

1,1,1,1,2,3,2,1,1,0,0,0,1,1,1,1,1

1,0,3,1,2,2,1,1,1,1,0,0,2,1,1,0,0

1,0,0,1,1,2,1,1,0,1,0,0,2,1,1,0,1

1,0,1,1,1,2,1,1,0,1,0,0,2,2,1,1,1

1,1,1,1,2,3,1,1,0,1,2,0,2,1,1,1,0

1,0,1,1,3,2,1,1,0,1,0,0,2,2,1,1,0

1,0,1,1,2,1,2,1,1,1,2,0,2,1,1,1,1

1,0,1,1,2,1,1,1,0,1,2,0,2,1,1,1,0

1,0,1,1,2,2,1,1,0,1,1,0,2,1,1,1,0

1,0,1,1,2,1,1,1,0,1,1,0,2,1,2,0,0

1,0,1,1,2,1,1,0,0,1,1,0,2,1,1.3,1,0

1,0,1,1,1,3,1,1,1,1,0,0,2,1,1,1,0

8,1,1,1,3,2,1,1,0,1,1,0,2,1,1,1,0

1,0,1,1,1,1,1,1,0,1,2,0,2,1,1,1,0

1,0,2,1,2,1,2,1,1,1,1,0,2,1,1,0,1

1,1,1,1,2,2,1,1,1,1,0,0,2,1,1,0,1

1,1,3,1,2,2,1,1,0,1,0,0,2,1,1,1,1

1,0,1,1,2,1,1,1,0,1,1,0,1,1,1,1,0

1,0,0,1,2,1,1,1,0,1,1,0,2,1,1,1,0

8,0,1,1,2,2,1,1,0,1,0,0,2,1,1,1,0

1,1,1,1,2,2,1,1,0,1,0,0,2,1,1,1,0

1,1,1,2,3,1,1,1,0,1,0,0,2,1,1,0,1

1,0,0,1,2,3,1,1,0,1,2,0,2,1,1,1,1

1,0,1,1,2,1,2,1,1,0,0,0,1,1,1,1,1

1,0,1,1,1,3,1,1,0,1,0,0,1,1,1,1,0

1,1,1,1,2,3,1,1,0,1,0,0,2,1,1,1,0

1,0,1,1,2,1,1,0,0,0,0,0,2,1,1,1,1

1,0,1,1,2,2,1,1,1,1,0,0,2,1,1,1,0

1,0,1,1,2,2,1,1,1,1,1,0,2,1,1,0,1

1,1,1,1,2,3,2,1,1,0,6,0,1,1,1,1,1

1,1,2,1,2,3,1,1,0,1,0,0,2,1,1,0,1

1,1,1,1,2,3,2,1,1,0,6,0,1,1,1,1,0

1,1,1,1,2,1,1,1,0,1,0,0,2,1,1,0,1

1,0,1,1,2,1,1,1,0,1,0,0,2,1,1,1,0

1,1,3,1,2,1,1,1,0,1,1,0,2,1,1,1,0

1,0,0,1,1,1,1,1,0,1,1,0,2,1,1,1,0

1,0,1,1,2,1,1,0,1,1,0,0,2,1,1,1,0

1,1,1,1,2,3,1,1,1,1,1,0,2,1,1,0,1

1,0,3,1,2,1,1,1,0,1,0,0,1,1,13,0,1

1,0,1,1,2,1,1,1,0,1,0,0,2,1,2,1,0

1,0,2,1,3,3,1,1,0,1,0,0,2,1,1,1,1

1,0,2,1,2,2,1,1,0,1,0,0,2,1,1,1,0

1,0,1,1,1,3,1,0,1,1,2,0,2,3,1,1,0

1,1,1,1,2,3,1,1,0,1,1,0,1,1,1,0,1

1,0,1,1,2,1,1,1,0,1,0,0,1,1,1,1,0

1,0,1,1,1,1,1,2,1,2,1,0,2,1,1,1,0

1,0,1,1,2,2,2,1,0,1,4,0,1,1,1,1,1

1,0,1,1,2,1,1,1,0,1,1,0,2,1,1,1,0

1,0,1,1,3,1,1,0,0,1,2,0,2,1,1,1,0

1,1,1,1,2,1,1,2,0,1,0,0,2,1,1,1,0

1,0,1,1,2,3,2,1,1,1,0,0,2,1,1,1,1

1,1,1,1,1,3,1,0,0,1,0,0,2,1,1,1,0

1,1,3,1,2,3,1,1,0,1,0,0,2,1,1,1,0

1,0,1,1,2,2,1,1,0,1,2,1,2,1,1,0,0

1,1,2,1,1,1,1,1,0,1,0,0,2,1,1,1,0

1,0,1,1,2,1,2,1,0,1,5,0,2,1,2,0,0

1,0,1,1,2,1,1,0,1,0,0,0,2,1,1,1,1

1,1,0,1,2,3,2,1,0,1,6,0,2,1,1,1,0

1,0,1,1,2,1,1,1,0,1,0,0,2,1,1,1,1

2,0,0,1,1,3,1,1,0,1,0,0,2,1,1,1,1

1,1,1,1,2,1,1,1,0,1,0,0,2,1,1,0,1

1,0,1,1,1,4,1,1,0,1,0,0,2,3,1,1,1

1,0,1,1,2,3,1,1,0,1,0,0,2,1,1,1,0

1,0,2,1,1,3,1,1,0,1,0,0,2,1,1,1,1

1,0,1,1,3,1,1,1,1,1,4,0,2,1,1,0,1

1,0,1,1,2,3,1,0,0,1,0,0,2,1,1,1,1

1,0,1,1,2,3,1,1,1,1,0,0,2,1,1,1,1

1,0,1,1,2,2,1,1,0,1,0,0,2,1,1,1,1

1,0,3,1,2,1,1,1,1,1,2,0,2,1,1,1,0

1,0,1,1,2,1,1,1,1,0,2,0,2,1,1,0,1

1,0,1,1,2,1,1,1,0,0,2,0,2,1,1,1,1

1,0,1,1,2,3,1,1,0,1,1,0,2,1,1,1,1

1,1,1,1,2,3,1,1,0,1,0,0,2,1,1,0,1

1,0,1,1,2,3,1,1,1,1,2,0,2,1,1,1,1

1,0,1,1,1,3,1,1,0,1,0,0,2,1,1,1,0

1,0,1,1,2,1,1,1,0,1,0,0,2,1,1,1,0

1,0,1,1,2,1,1,1,0,1,0,0,2,1,1,1,0

1,0,1,1,2,2,1,1,1,1,0,0,2,1,1,0,1

1,1,1,1,2,3,2,1,1,1,0,0,2,1,1,0,0

0,0,1,1,2,2,1,1,0,1,0,0,5,1,1,1,0

1,0,2,1,2,1,1,1,0,1,1,0,2,1,1,1,0

1,0,1,1,1,3,2,1,0,1,2,0,1,1,1,1,0

1,0,0,1,1,1,1,1,0,1,1,0,2,1,1,1,0

1,1,1,1,2,1,2,1,1,1,2,0,2,1,1,0,1

1,0,1,1,2,1,1,1,0,0,2,0,2,1,1,1,1

1,1,1,1,2,1,2,1,0,1,2,0,2,1,1,0,1

10,0,2,1,2,1,2,1,1,1,2,0,2,1,1,1,0

1,0,0,1,2,1,2,1,1,1,2,0,1,1,1,1,1

1,1,1,1,2,3,1,1,0,1,0,0,2,1,1,1,1

1,0,3,1,1,3,1,1,0,0,0,0,2,1,1,1,1

1,0,2,1,2,1,1,1,1,1,1,0,2,1,1,0,1

1,0,1,1,2,4,1,1,0,1,2,0,2,1,1,1,0

1,0,1,1,2,3,1,1,0,1,2,0,2,1,1,1,1

1,1,1,1,2,3,1,1,1,1,0,0,2,1,1,1,0

1,0,1,1,2,1,1,1,0,0,0,0,2,1,1,1,0

1,0,0,1,2,3,1,1,1,1,0,0,2,1,1,1,0

1,0,2,1,2,1,1,1,0,1,6,0,2,1,13,1,1

1,0,1,1,2,1,1,1,0,1,0,0,2,1,1,1,1

1,0,3,1,2,2,1,1,1,1,0,0,2,1,1,1,0

1,0,0,1,2,1,1,1,1,1,0,0,2,1,1,0,1

1,1,1,1,2,1,1,1,0,1,2,0,2,1,1,0,1

1,0,1,1,2,1,1,1,0,1,0,0,1,1,1,1,1

1,0,3,1,1,4,1,1,0,1,1,0,2,1,1,1,0

1,0,0,1,2,1,1,1,0,1,6,0,2,1,13,0,1

1,1,3,1,2,2,1,1,0,1,1,0,2,1,1,0,1

1,0,2,2,3,2,1,1,1,1,0,0,2,1,1,0,0

1,0,1,1,2,1,2,1,1,1,2,0,2,1,1,1,0

1,0,3,1,2,2,1,1,1,1,0,0,2,1,1,0,1

1,0,1,1,2,1,1,1,0,1,1,0,2,1,1,1,1

1,0,2,1,3,2,1,1,0,1,0,0,2,1,1,1,0

1,0,2,1,2,2,1,1,0,1,0,0,2,1,1,0,0

1,0,1,1,2,1,2,1,0,0,2,0,1,1,1,1,0

1,0,2,1,2,4,1,1,0,1,5,0,2,1,1,1,1

1,1,1,1,2,1,1,1,1,1,6,0,2,1,1,0,0

5,0,3,1,2,2,2,1,1,1,0,0,1,1,1,0,0

1,1,1,1,1,1,1,1,0,1,0,0,2,1,1,0,1

1,0,1,1,1,1,1,1,1,1,0,0,2,3,1,1,0

1,0,1,1,2,1,2,1,1,1,2,0,1,1,1,1,1

1,1,1,1,1,3,2,1,0,0,2,0,1,1,1,1,0

1,1,1,1,2,3,2,1,0,0,0,0,1,2,1,1,1

1,1,2,1,2,2,2,1,1,1,0,0,1,1,1,1,0

1,1,2,1,2,3,1,1,1,1,0,0,2,1,1,0,1

1,1,1,1,2,1,1,1,1,1,0,0,2,1,1,0,0

1,1,1,1,2,2,1,1,0,1,0,0,2,1,1,1,0

1,0,1,1,2,2,2,1,0,1,0,0,1,1,2,1,0

1,0,3,1,2,2,1,1,0,1,0,0,2,1,1,0,0

1,1,3,1,2,1,1,1,0,1,2,0,2,1,1,1,0

1,0,1,1,2,3,1,1,0,1,1,0,2,1,1,1,1

1,0,1,1,2,2,1,1,0,1,1,0,2,1,1,1,0

1,0,3,1,2,2,1,1,0,1,1,0,2,1,1,1,0

1,0,3,1,1,1,1,1,0,1,0,0,2,1,1,1,1

8,0,1,1,2,1,1,1,0,1,1,0,2,1,1,1,1

1,1,1,1,2,2,1,1,0,1,5,0,2,1,1,1,0

1,1,1,1,2,2,2,1,1,1,0,0,2,1,1,1,0

1,0,1,1,2,3,1,1,0,0,0,0,2,1,1,1,0

1,0,0,1,1,2,1,0,0,1,0,0,2,3,1,1,0

1,1,1,1,2,3,1,1,1,1,0,0,2,1,1,1,0

1,0,3,1,2,2,1,1,0,1,1,0,2,1,1,1,0

1,1,1,1,2,3,1,1,1,1,0,0,2,1,1,1,1

1,0,1,1,2,1,1,1,1,1,2,0,2,1,1,0,1

1,1,1,1,2,3,1,1,0,1,0,0,2,1,1,0,1

1,0,1,1,2,2,1,1,1,1,0,0,2,1,1,0,1

1,0,0,1,2,3,2,1,1,0,2,0,2,1,1,1,0

1,0,2,1,2,1,1,1,1,1,5,0,2,1,1,0,1

1,1,3,1,2,3,1,1,1,1,6,0,2,1,1,1,1

1,0,1,1,2,3,1,1,0,1,0,0,2,1,2,1,0

1,1,0,1,2,3,1,1,1,1,6,0,2,1,1,0,0

1,0,3,1,2,1,2,1,1,1,2,0,2,1,1,0,1

1,0,1,1,2,2,1,1,0,1,0,0,2,1,1,0,0

1,0,3,1,2,1,1,1,0,1,1,0,2,1,1,1,0

1,1,1,1,2,3,2,1,0,1,2,0,2,1,1,0,1

1,0,3,1,2,1,1,1,0,1,0,0,2,1,1,0,1

1,0,1,1,2,2,1,1,1,1,2,0,2,1,1,1,0

1,0,1,1,2,1,1,1,0,1,1,0,2,1,1,1,0

1,0,1,1,2,1,1,1,0,1,0,0,2,1,1,1,0

1,0,0,1,3,3,1,1,0,1,6,0,2,1,1,1,0

1,0,1,1,2,3,1,1,0,1,2,0,2,1,1,1,1

1,1,1,1,2,1,2,1,1,2,0,0,1,2,1,1,0

1,1,1,1,2,1,1,1,0,1,0,0,2,1,1,0,1

1,0,3,1,1,2,2,1,1,1,0,0,2,1,1,1,0

1,1,2,1,2,3,1,1,1,1,2,0,5,1,1,0,1

1,1,1,1,2,3,1,1,0,1,2,0,2,1,1,0,1

1,1,1,1,2,3,1,1,0,1,5,0,2,1,1,1,1

1,1,1,1,2,3,1,1,0,1,6,0,2,1,1,1,1

1,1,1,1,2,3,1,1,0,1,4,0,2,1,1,1,1

12,0,1,1,3,3,1,0,0,1,1,0,2,1,1,1,1

1,0,1,1,2,1,2,1,1,1,2,0,1,1,1,1,0

1,0,1,1,2,2,1,0,0,1,0,0,2,2,1,1,0

1,0,3,1,2,2,2,1,0,1,2,0,2,1,1,1,1

1,1,0,1,2,3,1,1,1,1,6,0,2,1,1,0,1

1,0,1,1,2,1,1,0,1,0,0,0,2,3,1,1,0

1,0,1,1,2,2,1,1,0,1,0,0,2,1,1,1,0

1,0,1,1,2,3,1,1,1,1,0,0,2,1,1,1,0

1,0,1,1,2,1,2,1,0,0,1,0,2,1,1,1,0

1,0,1,1,2,2,1,1,0,1,6,0,2,1,1,0,0

1,0,3,1,2,1,1,1,0,0,2,0,2,1,1,1,0

1,0,1,1,2,1,1,1,0,1,3,0,2,1,1,1,1

1,1,1,1,1,1,1,1,1,1,0,0,2,1,1,0,1

1,0,1,1,2,3,1,1,0,1,0,0,2,1,1,1,0

1,0,1,1,2,2,2,1,1,1,2,0,2,1,1,1,1

1,0,3,1,1,1,1,1,0,1,1,0,2,1,1,0,1

1,1,2,1,2,2,2,1,1,0,2,0,1,1,1,1,1

1,0,1,1,2,2,1,1,0,1,0,0,2,1,1,1,0

1,0,1,1,1,3,1,1,0,1,0,0,2,1,1,1,0

12,0,1,1,2,3,1,1,0,1,0,0,2,1,1,1,0

1,0,1,1,2,1,1,1,0,0,2,1,2,1,1,0,1

1,1,1,1,2,3,1,1,0,1,1,0,2,1,1,0,1

1,0,1,1,2,1,1,1,1,1,0,0,2,1,1,1,0

1,1,3,1,2,1,1,1,0,1,0,0,2,1,1,1,0

1,1,1,1,2,2,2,1,1,1,0,0,2,1,1,1,1

1,0,2,1,2,1,2,1,0,1,0,0,1,1,1,1,0

1,1,3,1,2,1,1,1,0,1,0,0,2,1,1,1,0

1,0,1,1,2,1,2,1,0,0,0,0,1,1,1,1,0

1,0,1,1,3,1,1,1,0,1,0,0,2,1,1,0,0

1,0,1,1,2,1,2,1,1,0,0,0,1,1,1,1,0

1,0,3,1,1,1,1,1,0,1,0,0,2,1,1,1,0

1,1,1,1,2,3,1,1,0,1,0,0,2,1,1,1,1

1,0,3,1,2,1,2,1,0,0,0,0,2,1,1,1,1

1,1,2,1,2,2,1,1,0,1,0,0,2,1,1,1,0

1,1,1,1,1,1,1,1,0,1,0,0,2,1,1,0,1

1,1,1,1,2,1,1,1,0,1,0,0,2,2,1,0,1

1,1,1,1,2,1,2,1,1,1,0,0,2,1,1,0,1

1,0,1,1,2,2,2,1,1,1,1,0,1,1,1,1,0

1,0,1,1,2,1,1,0,0,1,0,0,2,1,1,0,0

1,0,1,1,2,1,2,1,1,1,0,0,1,1,1,0,0

1,1,2,1,2,3,1,1,0,1,1,0,2,1,1,0,1

1,1,3,1,1,1,1,1,1,1,0,0,2,1,1,1,0

1,0,2,1,2,3,2,1,1,1,0,0,1,1,1,1,0

1,0,1,1,2,1,1,1,1,1,0,0,2,1,1,1,1

12,1,0,1,2,3,1,0,0,0,1,0,2,1,1,0,0

1,1,1,1,2,1,2,1,1,0,0,0,1,1,1,1,0

1,1,1,1,2,1,2,1,1,1,2,0,2,1,1,0,1

1,0,1,1,2,3,1,0,0,1,6,0,2,1,13,1,1

1,0,1,1,2,3,2,1,0,1,2,0,2,1,1,1,0

1,0,3,1,2,2,1,0,0,1,0,0,2,2,1,1,0

1,1,1,1,2,3,1,1,0,1,4,0,2,1,1,0,1

1,0,1,1,2,1,2,1,1,1,1,0,1,3,1,1,0

1,0,1,1,2,1,2,1,1,1,0,0,1,1,1,1,1

1,1,1,1,2,3,1,1,0,1,1,0,2,1,1,1,1

1,0,1,1,2,1,1,1,1,1,0,0,2,1,1,1,0

1,0,1,1,2,1,1,1,1,1,0,0,1,1,1,1,0

1,1,1,1,2,1,2,1,1,1,0,0,1,1,1,1,0

1,0,1,1,2,4,1,1,0,1,0,0,2,1,1,1,1

1,0,3,1,2,1,1,1,0,1,6,0,2,1,1,1,1

1,0,1,1,2,1,1,0,1,0,5,0,2,1,1,0,1

1,1,1,1,2,2,1,1,1,1,0,0,2,1,1,0,1

2,0,1,1,1,1,1,1,0,1,0,0,2,1,2,1,1

1,0,1,1,2,1,1,0,0,0,0,0,2,1,1,1,0

1,0,3,1,2,3,1,1,1,1,0,0,2,1,1,1,0

1,1,1,1,2,1,1,1,0,1,1,0,2,1,1,1,0

1,0,1,1,2,1,1,1,0,1,0,0,2,1,1,1,0

1,0,1,1,1,1,1,0,0,1,0,0,2,3,1,1,0

1,1,1,1,2,1,1,1,0,0,0,0,2,1,1,0,1

1,0,1,1,1,1,1,1,0,1,0,0,2,1,2,1,1

1,0,1,1,2,1,2,0,0,0,0,0,1,2,1,1,0

1,0,1,1,2,2,2,1,1,1,2,0,1,1,1,1,0

1,0,1,1,2,1,2,1,1,1,2,0,2,1,1,0,1

1,1,1,1,2,1,1,0,0,1,1,1,2,1,1,0,0

1,0,1,1,2,1,2,1,1,1,0,0,1,1,1,1,0

1,1,1,1,2,3,1,1,1,1,2,0,2,1,1,1,1

1,0,1,1,2,1,2,1,0,1,2,0,2,1,1,1,1

1,0,1,1,2,2,1,1,0,0,1,0,2,1,1,1,0

2,1,1,1,2,3,2,1,1,1,0,0,1,1,1,1,1

1,0,1,1,2,3,1,1,0,1,2,0,2,1,2,1,1

1,1,1,1,2,1,1,1,1,1,2,0,2,1,1,0,1

1,0,2,1,3,3,1,1,0,1,0,0,2,1,1,0,1

1,0,2,1,3,1,1,1,0,1,0,0,2,1,1,1,0

1,0,1,1,1,3,2,1,1,0,2,0,2,1,1,1,0

1,0,1,1,2,2,1,1,0,1,12,0,2,1,1,0,0

1,1,1,2,2,1,1,0,1,1,0,0,2,1,1,0,0

1,0,1,1,2,2,2,1,1,1,2,0,1,1,1,1,1

1,0,1,1,2,1,1,1,0,1,0,0,2,1,1,1,0

1,0,1,1,2,2,1,1,0,1,0,0,2,1,1,1,0

1,1,1,1,1,1,1,1,0,1,0,0,2,1,1,0,1

1,1,1,1,2,2,2,1,1,1,0,0,2,1,1,1,0

1,0,1,1,2,1,2,1,1,1,0,0,2,1,1,1,1

1,1,1,1,2,3,1,1,0,1,0,0,2,1,1,1,1

1,0,1,1,2,2,1,1,0,1,0,0,2,2,1,1,1

1,0,1,1,3,2,2,1,1,1,2,0,2,1,1,1,0

7,1,1,1,2,3,2,1,1,0,0,0,2,1,1,1,1

1,1,2,1,2,3,1,1,0,1,1,0,2,1,1,0,1

1,0,1,1,2,1,1,1,0,1,0,0,2,1,1,0,1

1,1,1,1,2,3,1,1,0,1,0,0,2,1,1,1,1

1,0,1,1,2,1,1,1,0,1,1,0,2,1,1,1,0

1,0,1,1,2,1,1,1,0,1,0,0,2,1,1,1,0

1,0,1,1,2,3,1,1,1,1,0,0,2,1,1,0,1

1,0,3,1,1,1,2,1,1,1,0,0,1,1,1,1,0

1,1,1,1,1,1,1,2,1,2,1,0,1,1,2,1,0

1,0,1,1,2,2,1,1,1,1,0,0,2,2,1,1,0

1,0,1,1,2,1,1,1,0,1,0,0,2,1,1,1,0

1,1,2,1,2,1,2,1,0,0,0,0,1,1,1,1,0

1,0,3,1,1,1,1,1,0,1,0,0,2,1,1,1,0

1,0,2,1,3,2,1,1,0,1,0,0,2,1,1,1,0

1,0,1,1,2,1,1,1,0,1,2,0,2,1,1,1,0

1,0,2,1,3,3,2,1,0,1,0,0,1,1,1,1,0

1,0,1,1,2,1,1,0,0,0,0,0,2,1,1,1,1

1,0,1,1,2,1,1,2,1,2,1,0,2,1,1,1,1

1,0,1,1,1,1,1,1,1,1,0,0,2,1,1,1,0

1,0,2,2,1,1,1,1,0,1,0,0,2,1,1,0,1

1,1,3,1,2,1,1,1,0,1,0,0,2,1,1,1,0

1,1,2,1,2,1,1,1,1,1,0,0,2,1,1,1,0

1,0,1,1,3,1,1,1,0,1,4,0,2,1,1,0,0

1,0,1,1,2,3,2,1,0,1,2,0,1,2,1,1,0

1,0,0,1,2,3,1,1,1,1,2,0,2,1,1,0,1

1,0,1,1,2,1,2,1,1,1,1,0,1,1,1,0,0

1,0,3,1,2,1,1,1,0,1,0,0,2,1,1,1,0

1,0,1,1,2,3,1,1,0,1,0,0,2,1,1,1,0

1,0,1,1,3,1,1,1,0,1,0,0,2,1,1,0,0

1,0,1,2,2,2,1,1,1,1,0,0,2,1,1,0,1

1,0,1,1,3,2,1,1,1,1,1,0,2,1,1,1,0

1,0,1,1,2,2,1,1,1,1,6,0,1,1,1,1,0

1,0,2,1,1,2,1,1,0,1,0,0,2,1,1,1,1

1,0,1,1,2,3,1,1,0,1,0,0,2,1,1,1,1

1,0,1,1,2,2,1,1,0,1,1,0,1,1,1,1,0

1,1,1,1,2,1,1,1,0,1,0,0,2,1,1,1,0

1,0,1,1,3,1,1,1,0,1,0,0,2,1,1,0,0

1,0,1,1,2,1,1,1,1,1,0,0,1,1,1,1,1

1,0,1,1,2,2,2,1,1,1,2,0,1,1,1,1,1

12,1,1,1,2,3,1,1,1,1,2,0,2,1,1,1,1

1,1,1,1,2,1,1,1,1,1,0,0,2,2,1,1,1

1,0,1,1,2,3,1,1,1,1,0,0,2,1,1,0,1

1,0,3,1,1,1,2,1,0,1,0,0,1,2,1,1,0

1,0,1,1,2,3,1,1,0,1,4,0,2,1,1,1,1

1,0,1,1,2,2,1,0,0,1,0,0,2,3,1,1,0

1,0,2,1,2,1,1,1,0,1,1,0,2,1,1,1,0

1,0,2,1,2,3,1,1,0,1,1,0,2,1,1,1,0

1,0,2,1,2,2,2,1,1,0,1,0,2,1,1,1,1

1,1,1,1,2,1,1,1,0,1,0,0,2,1,1,0,1

1,1,1,1,2,1,1,1,1,1,6,0,2,1,1,0,0

1,0,2,1,3,2,2,1,1,0,0,0,1,1,1,1,0

1,0,3,1,2,2,1,1,0,1,0,0,2,1,1,0,0

1,0,3,1,1,2,1,1,1,1,0,0,2,1,1,1,0

1,0,1,1,2,1,1,1,0,0,2,0,2,1,1,1,1

1,1,2,1,2,1,1,1,0,1,2,0,2,1,1,0,1

1,0,1,1,2,2,1,1,0,1,0,0,2,1,1,1,0

1,1,2,1,2,1,2,1,1,1,0,0,2,1,1,0,1

1,0,1,1,1,4,1,1,0,1,5,0,2,1,1,0,0

1,1,1,1,2,1,1,1,1,1,0,0,2,1,1,1,1

1,0,1,1,1,2,1,1,0,1,0,0,2,1,1,1,0

1,0,1,1,2,4,1,1,0,1,2,0,2,1,1,1,1

1,1,1,1,2,1,1,1,0,1,0,0,2,1,1,0,1

1,1,3,1,2,2,1,1,0,1,6,0,2,1,1,1,1

1,0,1,1,1,2,1,1,1,0,0,0,2,1,1,1,0

1,0,1,1,2,1,1,1,1,1,4,0,1,1,1,1,0

1,0,1,1,2,1,1,1,1,1,1,0,2,1,1,1,0

1,1,1,1,2,1,1,1,0,1,1,0,2,1,1,1,0

1,1,1,1,1,2,1,1,0,1,0,0,2,1,1,1,0

1,0,1,1,2,1,1,1,0,1,1,0,2,1,1,1,0

1,0,3,1,2,2,1,1,0,1,1,0,2,2,1,1,0

1,0,1,1,2,1,1,1,0,1,2,0,2,1,1,1,0

1,1,1,1,2,3,1,1,0,1,0,0,2,1,1,0,1

1,0,1,1,2,1,1,1,0,1,1,0,2,1,1,1,0

1,0,1,1,3,1,1,1,0,1,5,0,2,1,1,1,0

1,0,1,1,2,1,1,1,0,1,1,0,2,1,1,1,0

1,0,1,1,2,4,1,1,0,1,1,0,2,1,1,1,1

1,1,2,1,2,1,2,1,1,1,2,0,1,1,1,1,0

1,0,0,1,2,2,2,1,1,1,0,0,1,1,1,0,0

1,0,0,1,1,1,1,1,0,1,0,0,2,1,1,1,1

1,1,0,1,1,1,2,1,1,0,0,0,1,1,1,1,0

1,1,1,1,2,4,2,1,1,1,2,0,2,1,1,0,0

1,0,1,1,2,1,1,1,0,1,1,0,2,1,1,1,0

1,0,1,1,1,2,1,1,0,1,0,0,2,1,1,1,0

1,1,3,1,2,1,1,1,0,1,0,0,2,1,1,1,0

1,1,2,1,2,2,1,1,0,1,0,0,2,2,1,0,1

1,0,2,1,2,1,1,1,0,1,0,0,2,1,1,1,0

1,0,1,1,1,1,1,0,0,1,0,0,2,1,1,1,0

1,0,1,1,2,1,2,1,0,0,1,0,1,1,1,1,0

8,1,1,1,2,1,1,1,0,1,0,0,2,1,1,1,0

1,0,1,1,2,3,1,1,0,1,1,0,2,1,1,1,1

1,0,0,1,2,3,1,1,0,1,6,0,1,1,1,1,0

1,0,1,1,2,1,2,1,1,0,0,0,1,1,1,1,1

1,0,3,1,2,2,1,1,0,1,0,0,2,1,1,1,0

1,1,1,1,1,3,1,1,1,1,6,0,2,1,1,0,0

1,0,0,2,2,2,1,1,1,1,6,0,2,1,1,0,1

1,0,3,1,2,1,1,1,1,1,6,0,2,1,1,0,1

1,1,1,1,2,1,1,1,1,1,0,0,2,1,1,0,0

1,0,1,1,1,1,1,1,1,1,0,0,2,1,1,1,0

1,0,0,1,2,3,1,1,0,1,6,0,1,1,1,1,0

1,0,3,1,2,1,1,0,1,0,2,0,2,1,1,1,1

1,0,1,1,2,1,1,1,0,1,1,0,2,1,1,1,0

1,0,3,1,2,1,2,1,1,1,2,0,2,1,1,1,1

1,0,1,1,2,2,2,0,0,1,0,0,2,2,1,1,0

1,1,2,1,2,3,2,1,0,0,6,0,1,2,1,1,1

1,0,1,1,2,3,1,1,1,1,1,0,2,1,1,0,1

1,1,1,1,2,3,1,1,0,1,1,0,2,1,1,0,1

1,1,2,1,2,3,1,1,0,1,2,0,2,1,1,1,1

1,0,1,1,2,1,2,1,1,1,0,0,2,1,1,0,1

1,1,3,1,2,2,2,1,1,1,2,0,1,1,1,1,1

1,0,1,1,1,1,2,1,0,1,0,0,2,1,1,1,0

1,0,1,1,1,3,1,1,1,1,2,0,2,1,1,1,0

1,1,0,1,2,3,2,1,1,1,6,0,2,1,1,0,0

1,0,1,1,1,3,1,1,1,1,6,0,2,1,1,1,1

1,0,0,1,3,3,2,1,1,1,2,0,2,1,1,1,0

1,1,1,1,2,2,1,1,1,1,2,0,2,1,1,0,1

1,0,1,1,2,1,2,1,0,1,2,0,2,1,1,1,1

1,1,1,1,2,1,1,1,0,1,0,0,2,1,1,1,0

1,0,1,1,2,1,2,1,1,1,2,0,1,1,1,1,1

1,1,1,1,2,1,2,1,1,1,0,0,1,1,1,1,1

1,1,3,1,2,1,1,1,0,1,2,0,2,1,1,0,1

1,0,3,1,2,2,1,1,0,1,0,0,2,2,1,1,1

1,1,1,1,1,1,1,1,0,1,0,0,2,1,1,1,0

1,0,0,1,2,3,1,1,0,1,2,0,2,1,1,1,0

1,1,1,1,2,3,1,1,0,1,0,0,2,1,1,1,1

1,0,1,1,2,2,1,1,1,1,0,0,2,1,1,0,1

1,0,1,1,2,1,2,1,1,1,1,0,2,1,1,1,0

1,0,1,1,2,2,1,1,0,1,1,0,2,1,1,1,0

1,1,3,1,1,1,1,1,0,1,0,0,2,1,1,0,1

1,0,1,1,2,1,1,1,0,1,0,0,2,1,1,1,1

1,0,2,1,2,2,1,1,0,1,2,0,2,1,1,1,0

1,1,1,1,2,3,1,1,0,1,2,0,2,1,1,1,1

1,0,1,1,2,3,1,1,1,1,2,0,1,1,1,1,0

1,0,1,1,2,2,1,1,0,0,0,0,2,1,1,1,0

1,0,1,1,2,2,1,1,0,1,0,0,2,1,1,1,1

1,1,1,1,2,1,1,1,0,1,0,0,2,1,1,0,1

1,0,1,1,1,1,1,1,1,1,0,0,2,1,1,1,1

1,0,1,1,1,2,1,1,0,1,0,0,2,1,1,1,0

1,1,3,1,2,1,1,1,0,1,0,0,2,1,1,1,0

1,1,2,1,2,1,1,1,1,1,6,0,2,1,1,1,1

1,1,1,1,2,1,1,1,0,1,3,0,2,1,1,1,1

1,1,0,1,2,3,1,1,1,1,6,0,2,1,1,1,1

1,1,0,1,2,3,1,1,1,1,2,0,2,1,1,1,0

1,0,1,1,2,1,1,1,0,1,0,0,2,1,1,0,1

1,0,1,1,2,2,1,1,0,1,2,0,2,1,1,1,0

1,1,0,1,2,3,2,1,1,1,6,0,2,2,1,1,0

1,0,1,2,3,2,1,1,0,1,0,0,2,1,1,0,0

1,0,1,1,2,1,1,1,1,1,0,0,2,1,1,1,0

1,0,2,1,2,2,1,1,0,1,0,0,2,1,2,1,0

1,0,3,1,2,2,1,1,0,1,0,0,2,2,1,1,1

1,0,1,1,2,1,1,1,0,1,2,0,2,1,1,1,1

1,0,1,1,1,4,1,1,1,1,1,0,2,1,1,1,0

1,0,1,2,2,2,1,0,0,0,0,0,2,1,1,0,0

1,0,1,1,2,1,1,1,0,1,6,0,2,1,1,1,0

1,0,1,1,2,3,1,1,1,1,1,0,2,1,1,1,0

1,0,3,1,2,1,2,1,1,1,1,0,2,1,1,1,0

1,1,1,1,2,1,2,1,1,1,0,0,1,1,1,1,0

1,1,1,1,2,1,1,1,0,1,4,0,2,2,1,1,1

1,0,1,1,2,1,2,1,1,1,2,0,2,1,1,0,1

1,0,3,1,1,1,1,1,0,1,0,0,2,1,1,1,1

1,1,0,1,1,1,1,1,0,1,0,0,2,1,1,1,1

1,0,1,1,2,1,1,1,0,1,0,0,2,1,1,1,0

1,0,2,1,3,2,1,0,0,1,0,0,2,1,1,0,0

1,1,1,1,2,1,1,1,0,1,0,0,2,2,1,1,0

1,0,2,1,3,2,2,1,0,1,0,0,1,1,2,1,0

1,0,1,1,2,2,1,1,1,0,1,0,2,1,1,1,0

1,0,1,1,2,1,1,1,0,1,0,0,2,1,1,1,1

1,0,2,1,2,2,2,1,1,1,0,0,1,1,1,1,0

1,1,3,1,2,2,2,1,1,1,0,0,1,2,1,1,1

1,0,1,1,2,2,1,1,0,1,1,0,2,1,1,1,0

1,0,1,1,2,1,1,1,0,1,0,0,2,1,1,1,1

1,0,2,1,2,2,1,1,0,1,4,0,2,1,1,1,0

1,0,1,1,1,2,1,1,0,1,0,0,2,1,1,1,0

1,0,3,1,2,1,2,1,1,1,2,0,2,1,1,0,1

1,0,3,1,1,1,1,1,0,0,6,0,2,1,13,0,1

1,0,1,1,2,2,2,1,1,1,2,0,2,1,1,1,0

1,0,2,1,1,2,1,1,0,1,1,0,2,1,1,0,1

1,0,2,1,2,1,1,1,0,1,2,0,2,1,1,1,0

1,1,1,1,2,2,2,1,1,1,26,0,1,1,1,1,0

1,0,1,2,2,2,1,1,0,1,0,0,2,1,1,0,0

1,1,1,1,1,1,1,1,0,1,0,0,2,1,1,1,0

1,1,1,1,2,3,1,1,0,1,1,0,2,1,1,0,1

2,1,1,1,2,3,1,1,1,1,0,0,2,1,1,0,1

1,1,1,1,2,1,1,1,0,1,0,0,2,1,1,1,0

1,1,2,1,2,3,1,1,0,1,0,0,2,1,1,1,0

1,0,1,1,2,1,2,1,1,1,0,0,2,1,1,0,1

1,1,1,1,2,1,2,0,1,0,0,0,1,3,1,1,1

1,0,1,2,3,2,1,1,0,1,0,0,2,1,1,0,1

1,0,1,1,2,1,1,1,0,1,0,0,2,1,1,1,1

1,0,3,1,2,1,1,1,0,1,1,0,2,1,1,1,0

1,0,1,1,2,1,1,1,1,1,0,0,2,1,1,1,0

1,0,1,1,3,1,1,0,0,1,0,0,2,1,1,1,0

1,1,0,1,2,3,1,1,1,1,2,0,2,1,1,0,0

1,1,1,1,2,2,2,1,1,1,0,0,2,1,1,0,0

1,0,0,2,3,3,1,1,0,1,2,0,2,1,1,0,0

1,0,1,1,2,1,1,1,0,0,2,0,2,1,1,1,0

1,0,0,1,2,1,2,1,1,1,2,0,1,1,1,1,0

1,0,1,1,2,1,1,1,0,1,4,0,2,1,1,1,1

1,1,1,1,2,2,1,1,1,1,0,0,2,1,1,0,0

1,0,3,1,2,2,1,1,0,1,6,0,2,1,1,1,1

1,1,1,1,2,1,1,1,0,1,2,0,2,1,1,0,1

2,0,1,1,1,1,1,1,0,1,0,0,2,1,1,1,0

1,0,1,1,2,1,1,1,0,1,2,0,2,1,1,1,1

1,0,1,1,1,1,1,1,0,1,0,0,2,1,1,1,1

1,0,1,1,2,1,2,1,1,1,1,0,1,1,1,1,0

1,0,1,1,2,1,1,1,0,1,0,0,1,1,1,1,0

1,1,1,1,2,1,2,1,0,1,2,0,1,1,1,1,0

1,0,3,1,2,1,1,1,0,1,1,0,2,1,1,1,0

1,1,1,1,1,1,1,1,0,1,0,0,2,1,1,1,0

1,0,1,1,1,2,1,1,0,1,0,0,2,1,1,1,0

1,0,1,1,2,1,2,1,1,1,2,0,2,1,1,0,1

1,0,0,2,3,2,1,1,1,1,0,0,2,1,1,0,0

1,0,1,1,2,1,2,1,1,0,0,0,1,1,1,1,0

1,1,1,1,2,1,1,1,0,0,0,0,2,1,1,0,1

1,0,3,1,1,1,1,1,0,1,0,0,1,1,1,1,1

1,1,3,1,2,2,1,1,0,1,2,0,2,2,1,1,0

1,0,3,1,2,1,1,1,0,0,2,0,2,1,1,1,1

1,0,1,1,2,1,1,1,1,0,2,0,2,1,1,1,1

1,0,0,1,2,3,1,1,0,1,2,0,2,1,1,1,0

0,0,1,1,2,1,2,0,0,0,2,0,2,1,1,1,1

1,0,3,1,2,2,2,1,1,1,2,0,1,1,1,1,0

1,0,0,1,2,3,2,1,1,1,2,0,2,1,1,1,0

1,0,0,1,2,3,2,1,1,1,2,0,2,1,1,1,0

1,0,1,1,2,2,1,1,0,1,0,0,2,1,1,1,0

1,0,1,1,2,3,2,1,1,1,6,0,2,1,1,1,0

1,0,1,1,3,2,1,1,1,1,5,0,2,1,1,0,1

1,1,0,1,2,3,1,1,0,1,6,0,2,1,1,0,1

1,0,3,1,1,2,1,1,0,1,0,0,2,1,1,0,0

1,0,1,1,2,1,1,1,0,1,0,0,1,1,1,0,0

1,0,1,1,2,3,1,1,0,1,2,0,2,1,1,1,0

1,0,1,1,2,1,2,0,1,0,0,0,2,1,1,0,1

1,0,2,1,2,1,1,1,0,1,1,0,2,1,1,1,0

1,1,1,1,1,3,1,1,0,1,6,0,2,1,1,1,0

1,1,3,1,2,1,1,1,1,1,0,0,2,1,1,0,0

1,1,1,1,2,1,2,1,1,1,2,0,2,1,1,1,1

1,1,1,1,2,3,1,1,0,1,0,0,2,1,1,1,1

1,0,2,1,2,1,1,2,1,2,1,0,2,1,1,1,0

1,1,3,1,1,3,1,0,0,1,6,0,2,1,1,1,0

1,1,1,1,2,1,1,1,0,1,0,0,2,1,1,0,0

1,1,0,1,2,3,1,1,1,1,6,0,2,1,1,1,0

8,0,1,2,3,2,1,1,0,0,0,0,2,1,1,0,1

1,1,1,1,2,2,2,1,1,1,0,0,2,1,1,0,1

1,0,2,1,1,2,2,1,0,1,0,0,1,1,1,1,0

1,0,1,1,2,1,1,1,1,1,0,0,2,1,1,1,1

1,0,2,1,1,3,1,1,0,1,2,0,2,2,1,1,0

1,1,3,1,1,1,1,1,0,0,0,0,2,1,1,1,1

1,0,1,1,2,3,2,1,0,1,0,0,1,1,1,1,0

1,0,1,1,2,2,2,1,1,1,0,0,1,1,1,1,0

1,0,3,1,2,1,1,1,0,1,0,0,2,1,1,1,1

1,0,1,1,2,1,1,1,0,1,0,0,2,1,1,1,0

1,0,1,1,2,1,2,1,1,1,0,0,1,1,1,0,0

1,0,2,1,1,3,2,1,1,1,2,0,2,1,1,1,0

1,1,1,1,1,3,1,1,0,1,6,0,1,1,1,1,1

1,1,1,1,2,1,2,1,0,1,0,0,1,1,1,1,0

1,0,2,1,3,2,1,1,0,1,2,1,2,1,1,0,0

1,0,1,1,2,4,1,1,0,1,5,0,2,1,1,1,1

1,0,2,1,2,1,2,1,1,1,0,0,1,1,1,1,0

1,1,1,1,2,3,1,1,0,1,0,0,2,1,1,0,1

1,0,1,1,2,1,1,1,1,1,2,0,2,1,1,1,0

1,0,1,1,3,1,1,1,0,1,1,0,2,1,1,1,0

1,0,3,1,1,1,1,0,0,1,0,0,2,2,1,1,0

1,1,1,1,2,1,2,1,1,1,0,0,2,1,1,0,0

1,0,1,2,3,2,1,1,0,1,0,0,2,1,1,0,1

1,0,1,1,2,1,1,0,0,0,1,0,2,1,1,1,1

1,0,3,1,2,3,2,1,0,1,6,0,2,1,13,0,0

1,1,1,1,2,2,1,1,1,1,0,0,2,1,1,0,0

1,0,1,1,2,1,1,1,0,1,0,0,2,1,13,1,1

1,0,3,1,2,1,1,1,0,1,1,0,2,1,1,1,0

2,0,2,1,2,1,2,1,1,1,0,0,1,1,1,1,0

1,0,3,1,1,1,1,1,0,1,1,0,2,1,1,1,1

1,1,1,1,2,1,1,1,1,1,0,0,2,1,1,1,1

1,0,1,1,2,1,1,1,0,1,0,1,2,1,1,0,0

1,1,3,1,2,1,2,1,1,1,2,0,1,1,1,1,1

1,0,1,2,3,1,1,1,0,1,0,0,2,1,1,0,1

2,0,1,1,2,1,1,1,1,1,0,0,1,1,13,0,1

1,0,1,1,2,2,2,0,1,1,0,0,1,1,2,1,0

1,1,1,1,1,1,1,2,1,1,2,0,2,1,1,1,0

1,0,1,1,1,1,1,1,0,1,0,0,2,3,1,1,1

1,1,1,1,2,1,2,1,1,1,6,0,1,1,1,1,0

1,0,1,1,1,1,1,1,0,1,1,0,2,1,1,1,0

1,0,1,1,2,2,1,1,1,1,0,0,2,1,1,1,0

1,0,1,1,2,2,1,1,0,1,0,0,2,1,1,1,0

12,0,2,2,3,1,1,0,1,1,0,0,2,3,1,1,1

1,0,1,1,2,1,1,1,1,1,0,0,2,1,1,1,0

2,0,1,1,3,1,1,1,0,1,0,0,2,1,13,0,1

1,0,1,1,2,1,1,1,0,1,0,0,2,1,1,1,1

1,0,3,1,2,1,1,1,1,1,2,0,2,1,1,0,1

1,0,1,1,3,3,1,1,0,1,0,0,2,1,1,1,1

1,0,2,1,2,1,1,1,0,1,0,0,2,1,1,1,1

1,1,1,1,2,1,1,1,0,1,0,0,2,1,1,1,0

1,1,3,1,2,1,1,1,1,1,0,0,1,1,1,1,0

1,1,2,1,2,2,1,1,0,1,1,0,2,1,1,0,1

1,1,3,1,1,4,1,1,0,1,0,0,2,1,1,1,1

1,1,1,1,1,1,1,1,0,1,1,0,2,1,1,0,1

1,0,1,1,2,1,2,0,0,0,4,0,1,2,1,1,0

1,0,0,1,2,3,2,1,1,1,2,0,2,1,1,1,0

1,1,3,1,2,1,1,1,0,1,0,0,2,1,1,1,0

1,0,1,1,2,2,1,1,0,1,0,0,2,1,1,1,0

1,1,1,1,2,1,1,1,1,1,0,0,2,1,1,1,1

1,1,1,1,2,1,1,1,0,1,0,0,2,1,1,1,0

1,0,3,2,1,2,1,1,0,1,0,0,2,1,1,1,0

1,0,1,1,2,1,2,1,1,1,2,0,2,1,1,0,1

1,0,3,1,2,1,1,1,0,1,0,0,2,1,1,1,0

1,0,1,1,2,1,1,1,1,1,0,0,2,1,1,1,0

1,0,0,1,1,1,1,0,0,1,1,0,2,1,1,1,1

1,0,1,1,2,1,2,1,1,1,2,0,1,1,1,1,1

1,1,1,1,2,3,1,1,0,1,0,0,2,1,1,1,1

1,0,2,1,2,2,1,1,1,1,0,0,2,1,1,0,0

1,0,1,1,2,1,1,1,1,1,0,0,2,1,1,1,0

1,0,1,1,2,1,1,1,1,1,2,0,2,1,1,0,1

1,1,3,1,2,1,2,1,1,0,0,0,1,1,1,1,0

1,0,0,1,1,1,1,1,0,1,0,0,2,1,1,1,0

1,0,2,1,2,3,1,1,0,1,0,0,2,1,1,1,1

1,0,3,1,2,2,1,1,1,1,0,0,2,1,1,0,1

1,1,0,1,3,3,1,1,0,1,6,0,2,2,1,1,0

1,0,1,1,2,2,2,1,1,1,2,0,2,1,1,1,1

1,0,1,1,2,1,2,1,1,1,0,0,1,1,1,1,0

1,1,2,1,2,1,1,1,0,1,0,0,2,1,1,0,0

1,0,1,1,1,1,2,1,1,0,0,0,2,1,13,0,1

1,0,1,1,2,1,2,1,1,1,2,0,2,1,1,1,1

1,1,1,1,2,2,2,1,0,1,0,0,1,1,1,1,1

1,0,1,1,2,1,1,0,0,1,1,0,2,1,1,0,0

1,0,3,1,1,3,1,1,1,1,0,0,2,1,1,0,1

1,1,1,1,2,3,1,1,1,1,6,0,2,2,1,0,1

1,0,3,1,2,1,2,1,0,0,2,0,1,1,1,1,0

1,1,1,1,2,2,1,1,0,1,0,0,2,1,1,1,0

1,0,1,1,2,2,1,1,1,1,0,0,2,1,1,1,0

1,1,1,1,2,1,2,1,1,1,0,0,1,1,1,1,0

1,1,1,1,2,2,1,1,0,1,1,0,2,1,1,0,1

1,0,1,1,2,1,2,1,1,1,2,0,1,1,1,1,0

1,1,3,1,2,1,1,1,1,0,0,0,2,1,1,0,1

1,0,2,1,3,1,1,1,0,1,6,0,2,1,13,0,0

1,1,1,1,1,1,1,1,0,1,0,0,2,1,1,1,0

1,1,3,1,1,3,1,1,1,1,2,0,2,1,1,0,0

1,0,1,1,2,3,1,1,0,1,6,0,2,1,1,1,1

1,0,3,1,2,1,1,1,0,1,1,0,2,1,1,1,0

1,1,1,1,2,2,1,1,1,1,0,0,2,1,1,1,0

1,1,1,1,2,3,2,1,1,1,2,0,1,1,1,1,1

1,0,1,1,2,1,1,1,0,1,2,0,2,1,1,1,0

1,1,2,1,2,2,2,2,1,1,0,0,2,1,1,1,0

1,0,1,1,2,1,1,1,0,1,0,0,2,1,1,0,1

1,0,1,1,2,1,1,0,1,1,2,0,2,2,1,1,0

1,0,1,1,2,1,1,1,0,1,2,0,2,1,1,0,1

1,0,1,2,1,2,2,0,0,1,0,0,1,1,1,0,0

1,0,2,1,2,1,1,1,1,1,0,0,2,1,1,0,1

1,1,1,1,2,1,2,1,1,1,0,0,2,1,1,1,1

1,0,1,1,2,1,1,0,0,0,0,0,2,3,1,1,1

8,0,3,1,2,2,1,1,0,1,1,0,2,1,1,0,1

1,0,3,1,2,1,1,1,0,1,2,0,2,1,1,1,0

1,0,1,1,2,2,2,1,1,1,0,0,1,1,1,1,0

1,0,1,1,3,1,1,1,0,1,1,0,2,1,1,0,0

1,1,2,1,2,2,1,1,0,1,0,0,2,1,1,1,0

1,0,3,1,2,2,1,1,0,1,0,0,2,1,1,1,0

1,0,1,1,2,4,1,1,0,1,0,0,2,1,1,1,1

1,1,3,1,2,3,1,1,1,1,2,0,2,1,1,0,1

1,0,0,1,1,2,2,1,0,1,0,0,2,1,1,1,1

1,1,1,1,1,3,2,1,1,1,4,0,1,1,1,1,0

1,0,1,1,3,3,1,1,0,1,0,0,2,1,13,1,0

1,0,1,1,2,4,1,1,0,1,0,0,2,1,1,1,1

1,0,1,1,2,2,1,1,0,1,1,0,2,1,1,1,0

1,0,1,1,2,2,1,1,0,1,1,0,2,2,1,1,0

1,0,1,1,2,1,1,1,0,1,1,0,2,1,1,1,0

1,1,0,1,2,3,1,1,0,1,6,0,2,2,1,1,0

1,1,1,1,2,4,2,1,1,1,5,0,2,1,1,0,0

1,0,1,1,2,1,2,1,1,1,0,0,2,1,1,1,0

1,0,1,1,2,1,1,1,0,1,0,0,2,1,1,1,1

1,0,1,1,2,1,1,1,0,1,0,0,2,1,1,1,0

1,0,1,1,2,1,2,1,0,1,0,0,2,1,1,1,1

1,0,0,1,2,2,1,1,0,1,0,0,2,1,1,1,0

1,0,3,1,2,1,1,1,1,1,2,0,2,1,1,1,0

1,0,1,1,2,3,1,1,1,1,1,0,2,1,1,1,0

1,1,1,1,1,1,1,1,0,1,0,0,2,1,1,0,1

1,0,1,1,1,2,1,1,1,1,0,0,2,1,1,0,1

1,1,1,1,2,1,2,1,1,1,0,0,1,1,1,1,0

1,1,0,1,2,1,1,1,1,1,1,0,2,1,1,1,1

1,1,0,1,2,3,2,1,0,1,2,0,1,1,1,1,0

1,0,1,1,2,1,1,0,0,1,6,0,2,1,1,1,0

1,0,1,1,2,1,1,1,0,1,2,0,2,1,1,1,0

1,0,1,1,2,1,1,1,0,1,1,0,2,1,1,0,0

1,0,3,1,2,1,2,1,1,1,2,0,1,1,1,1,0

1,0,1,1,2,2,1,1,0,1,2,0,2,1,1,0,1

1,0,1,1,2,1,1,1,0,1,2,0,2,1,1,1,0

1,1,1,1,2,3,1,1,0,1,2,0,2,1,1,1,0

2,1,1,1,1,1,1,1,0,1,1,0,2,1,1,0,1

1,0,2,1,2,1,1,1,0,1,2,0,2,1,1,1,0

1,0,2,1,2,1,1,0,1,1,0,0,2,2,1,1,0

1,0,1,1,2,1,1,1,0,1,2,0,2,1,1,1,0

1,1,1,1,2,1,2,1,1,1,0,0,1,1,1,1,1

1,1,1,1,2,1,1,1,1,1,0,0,2,1,1,0,1

1,0,1,1,2,1,1,1,1,1,2,0,2,1,1,1,1

1,0,1,1,2,1,1,1,0,1,6,0,2,1,1,1,0

1,1,1,1,2,1,2,1,0,0,0,0,2,1,1,0,1

1,0,1,1,2,1,1,1,0,1,2,0,2,1,1,1,0

1,0,1,1,2,1,1,1,0,0,2,0,2,1,1,1,0

1,0,1,1,2,2,1,1,0,1,0,0,2,1,1,1,0

1,0,2,1,2,1,1,1,1,0,0,0,2,1,13,1,0

1,0,3,1,2,1,1,1,1,1,2,0,2,1,1,0,0

1,0,1,1,2,1,1,1,1,1,0,0,2,1,1,0,1

1,0,1,1,2,4,1,1,0,1,0,0,2,1,1,1,1

1,1,0,1,1,1,1,0,1,1,0,0,2,2,1,1,0

1,0,1,1,2,1,1,1,0,1,0,0,2,1,13,0,0

1,0,1,1,2,1,2,1,0,0,6,0,2,1,13,0,1

1,0,1,1,2,2,2,1,0,0,0,0,1,1,1,1,1

1,1,1,1,2,3,1,1,0,1,0,0,2,1,1,0,1

1,0,1,1,2,1,1,0,0,0,0,0,2,1,1,1,1

1,0,1,1,2,1,2,1,1,1,0,0,2,1,1,1,0

1,0,1,1,1,3,1,1,0,1,0,0,2,1,1,1,1

1,0,2,1,2,1,1,1,0,1,2,0,2,1,1,1,0

1,1,1,1,2,3,1,1,0,1,5,0,2,1,1,0,1

1,0,3,1,2,1,1,1,0,1,6,0,2,1,13,1,1

1,0,1,1,2,1,2,1,0,0,0,0,2,1,13,1,1

1,1,1,1,2,3,1,1,0,1,0,0,2,1,1,1,0

1,0,1,1,2,1,1,1,0,1,0,0,2,1,1,1,0

1,1,3,1,2,1,2,1,0,1,0,0,1,1,1,1,0

1,1,1,1,2,2,2,1,1,1,2,0,2,1,1,1,1

1,0,1,1,2,3,1,1,1,1,0,0,2,1,1,1,0

1,1,1,1,2,2,1,1,0,1,2,0,2,1,1,0,1

1,0,1,1,2,1,1,1,1,1,2,0,2,1,1,1,0

1,0,1,1,2,1,2,1,0,1,6,0,2,1,1,1,1

1,0,1,1,2,1,1,1,1,1,0,0,2,2,1,0,1

1,1,1,1,2,1,2,1,1,1,2,0,2,1,1,0,1

1,0,1,2,3,3,1,1,1,1,2,0,2,2,1,0,0

1,1,1,1,2,1,2,1,1,1,0,0,1,1,1,1,1

1,1,1,1,2,3,1,1,0,1,0,0,2,1,1,1,1

1,1,3,1,1,3,2,1,0,1,2,0,1,1,1,1,1

1,0,1,1,2,1,1,0,1,1,4,0,2,1,1,0,1

1,1,1,1,2,3,2,1,0,0,0,0,1,1,1,1,1

1,0,1,1,2,1,2,1,0,1,2,0,1,1,1,1,0

1,0,3,1,2,1,2,0,0,0,1,0,1,2,1,1,0

1,0,1,1,2,1,1,1,0,1,1,0,2,1,1,1,1

1,0,1,1,1,3,2,1,1,0,2,0,2,1,1,1,0

1,1,1,1,2,1,1,1,0,1,0,0,2,1,1,1,0

1,1,0,1,2,3,1,1,1,1,6,0,2,1,1,0,0

1,0,1,1,2,1,1,1,0,0,0,0,2,2,1,1,1

1,1,1,1,2,1,1,1,1,1,2,0,1,1,1,1,0

1,0,1,1,2,1,1,1,0,1,0,0,2,1,2,0,0

1,0,1,1,2,1,1,1,0,1,0,0,2,1,1,1,0

1,1,1,1,2,1,1,1,0,1,0,0,2,1,1,1,0

1,0,2,1,2,1,2,1,0,1,6,0,2,1,1,0,1

1,0,1,1,2,1,1,0,0,1,0,0,2,2,1,1,0

1,1,0,1,2,2,2,1,1,1,2,0,2,1,1,1,0

1,0,1,1,2,1,2,1,1,1,0,0,2,1,1,0,1

1,1,1,1,2,1,2,1,1,1,0,0,2,1,1,0,1

1,0,1,1,2,2,2,1,1,1,2,0,1,1,1,1,1

1,1,3,1,2,2,1,1,0,1,0,0,2,1,1,0,1

1,1,1,1,2,3,2,1,0,1,6,0,1,1,1,1,1

1,1,2,1,2,1,1,1,1,1,0,0,2,1,1,0,1

1,1,3,1,2,1,1,1,1,1,6,0,1,1,1,1,0

1,0,2,1,2,2,1,1,0,1,1,0,2,1,1,1,1

1,1,1,1,2,2,2,1,1,0,0,0,1,1,1,1,0

1,1,0,1,2,3,1,1,1,1,2,0,2,1,1,1,0

7,1,1,1,2,3,1,1,0,1,6,0,2,1,1,1,1

1,0,1,1,2,3,1,1,0,1,0,0,2,1,1,1,1

1,1,1,1,2,3,2,1,1,1,0,0,2,1,1,1,0

1,0,1,1,2,2,1,1,0,1,0,0,2,1,1,0,1

1,1,1,1,2,3,2,1,1,1,4,0,2,1,1,0,1

1,0,1,1,2,3,2,1,0,1,2,0,2,1,1,1,1

1,1,2,1,2,3,1,1,0,1,5,0,2,1,1,0,1

1,1,0,1,2,3,2,1,1,1,6,0,2,3,1,0,0

1,1,0,1,2,3,2,1,1,1,2,0,2,1,1,0,1

1,1,1,1,2,1,1,1,0,1,0,0,2,1,1,1,1

1,0,1,1,2,1,1,1,0,1,0,0,2,1,1,1,1

1,1,0,1,2,3,1,1,0,1,2,0,2,1,1,1,1

1,1,1,1,2,3,2,1,0,0,2,0,2,1,1,1,0

1,1,3,1,2,1,1,1,1,1,0,0,2,2,1,1,1

1,0,1,1,2,1,2,1,1,1,0,0,1,1,1,1,0

1,0,1,1,2,2,1,1,0,1,0,0,1,1,1,1,0

1,0,1,1,2,1,1,1,0,1,1,0,2,1,1,1,1

1,0,0,1,2,3,2,1,1,1,0,0,2,1,1,0,0

1,0,1,1,2,1,1,1,0,1,0,0,2,1,1,1,1

1,1,1,1,2,1,1,1,0,1,0,0,2,1,1,1,1

1,0,1,1,2,3,1,1,0,1,2,0,2,1,1,1,1

1,0,1,1,1,2,1,1,0,1,1,0,2,1,1,0,0

1,1,3,1,2,3,1,1,0,1,0,0,2,1,1,1,0

1,0,1,1,2,1,1,1,1,1,0,0,2,1,1,1,1

1,0,3,1,2,3,1,1,0,1,0,0,2,1,1,1,1

1,1,1,1,2,1,1,1,0,1,0,0,2,1,1,0,1

1,0,1,1,2,1,1,0,0,1,0,0,1,1,13,0,1

1,0,1,1,2,3,1,1,0,1,0,0,2,1,1,1,1

1,0,1,1,2,1,2,1,1,0,1,0,1,1,1,1,0

1,0,3,1,2,3,1,1,0,1,5,0,2,1,1,1,0

1,1,1,1,2,3,1,1,0,1,5,0,2,1,1,0,1

1,0,1,1,2,1,2,1,1,1,0,0,2,1,1,1,0

1,0,0,1,2,2,1,1,0,1,0,0,2,2,1,1,0

1,1,1,1,2,4,1,0,0,1,6,0,2,1,1,1,1

1,0,2,1,1,2,1,1,0,1,2,0,2,2,1,1,1

1,0,1,1,2,1,2,1,1,1,0,0,1,1,2,1,0

8,0,1,1,1,2,1,1,0,1,0,0,2,1,1,1,0

1,1,1,1,2,1,1,1,0,1,0,0,2,1,1,0,1

1,0,1,1,2,1,1,1,0,1,0,0,2,1,1,1,1

1,0,3,1,1,2,1,1,1,1,0,0,2,1,2,0,1

1,1,2,1,2,3,1,1,0,1,3,0,2,1,1,0,1

1,0,1,1,2,2,2,1,1,1,1,0,1,1,1,1,0

1,1,1,1,2,2,1,1,0,1,3,0,2,1,1,1,1

1,0,1,1,2,1,1,1,0,1,0,0,2,1,1,1,0

1,1,0,1,2,3,1,1,1,1,2,0,2,2,1,0,0

1,1,2,1,2,3,1,1,0,1,4,0,2,1,1,1,1

1,0,1,1,2,1,1,1,1,0,2,0,2,1,1,0,1

1,0,1,1,2,1,2,1,1,1,2,0,1,1,1,1,0

1,1,1,1,2,2,1,1,0,1,0,0,2,1,1,1,0

1,0,0,2,3,3,1,1,0,1,2,0,2,1,1,1,0

1,1,1,1,2,2,1,1,1,1,0,0,2,1,1,0,0

1,0,2,1,2,2,2,1,1,1,0,0,2,1,1,1,0

1,0,1,1,1,2,1,1,0,1,0,0,2,1,1,1,0

1,0,1,1,2,3,1,1,1,1,0,0,2,1,1,1,0

1,1,1,1,2,2,1,1,1,1,0,0,2,1,1,0,1

1,0,1,1,2,2,2,1,0,1,0,0,2,1,1,1,1

1,0,1,1,2,3,1,1,0,1,0,0,2,1,1,0,1

1,0,1,1,2,2,1,1,1,1,2,0,2,1,1,1,0

1,1,0,1,2,3,1,1,0,1,6,0,2,1,1,1,0

1,0,1,2,3,2,1,1,1,1,0,0,2,1,1,0,0

1,0,1,1,2,1,1,1,0,0,2,0,2,1,1,1,1

1,0,1,1,2,1,1,1,0,0,0,0,2,1,1,1,1

1,1,3,1,2,1,2,1,0,0,0,0,1,1,1,1,0

1,0,1,1,2,1,1,1,1,1,3,0,2,1,1,1,0

1,1,0,1,2,3,1,1,0,1,0,0,2,1,1,1,0

1,0,1,1,2,1,1,0,0,1,0,0,2,2,1,1,0

1,1,2,1,1,1,1,1,0,1,0,0,2,1,1,0,0

1,0,1,1,2,1,1,1,1,1,2,0,2,2,1,1,0

1,1,3,1,1,3,1,1,1,1,2,0,2,1,1,0,0

1,0,1,1,2,1,2,1,0,1,0,0,1,2,1,1,1

1,0,1,1,2,2,1,1,0,1,1,0,2,1,1,1,0

1,1,0,1,2,3,2,1,1,1,6,0,2,1,1,0,0

1,0,3,1,2,1,1,1,0,1,1,0,2,1,1,1,0

1,0,1,1,2,1,1,1,0,1,1,0,2,1,1,0,1

1,1,1,1,1,1,1,1,0,1,0,0,2,1,1,1,1

12,0,3,1,1,1,2,0,0,1,0,0,1,1,1,1,0

1,1,1,1,2,1,2,1,1,1,5,0,2,1,1,0,0

1,0,3,1,2,1,1,0,0,0,2,0,1,1,1,1,1

1,1,1,1,2,2,1,1,0,1,2,0,2,1,1,0,1

1,0,0,1,1,2,1,1,0,1,0,0,2,1,1,1,0

1,0,1,1,2,3,2,1,1,1,0,0,1,1,1,1,1

1,1,1,1,2,1,1,1,1,1,1,0,2,1,1,0,0

1,1,0,1,2,3,1,1,1,1,2,0,2,1,1,0,0

1,0,0,1,2,3,2,1,1,1,6,0,2,1,1,1,0

1,0,1,1,2,2,2,1,1,1,0,0,1,1,1,1,0

1,0,1,1,2,1,1,1,0,1,1,0,2,1,1,1,1

1,0,1,1,2,1,1,1,0,1,0,0,2,1,1,1,0

1,0,1,1,2,2,2,1,1,1,0,0,2,2,1,1,0

1,1,1,1,2,3,1,1,0,1,0,0,2,1,1,1,1

1,0,3,1,1,1,1,1,0,1,6,1,2,1,1,0,1

1,0,1,1,2,3,1,1,0,1,0,0,2,1,1,1,0

1,0,1,1,2,2,1,1,0,1,0,0,2,1,1,1,0

1,1,3,1,1,1,1,1,0,1,2,0,2,1,1,1,0

1,0,1,1,2,1,1,1,0,1,1,0,2,1,1,1,1

1,0,1,1,2,1,1,1,1,1,2,0,2,1,1,1,0

1,0,1,1,2,2,1,1,0,1,0,0,2,1,1,1,0

1,1,0,1,2,2,1,1,0,1,0,0,2,1,1,0,1

1,1,0,1,2,3,1,1,1,1,6,0,2,1,1,0,0

1,0,1,1,2,1,2,1,1,1,2,0,1,1,1,1,1

1,0,1,1,2,2,2,1,1,1,0,0,2,1,1,1,1

1,0,0,1,2,1,1,0,0,1,1,0,2,1,1,1,1

1,0,2,2,1,3,1,1,0,1,1,0,2,1,13,1,1

1,1,1,1,2,3,1,1,0,1,6,0,2,1,1,0,1

1,0,1,1,2,2,1,1,0,0,0,0,2,1,1,0,0

1,0,1,1,2,1,1,1,1,0,2,0,2,1,1,1,0

1,1,1,1,2,1,1,1,0,1,0,0,2,1,1,1,0

1,0,1,1,1,2,2,1,1,1,0,0,1,1,1,1,0

1,0,2,1,2,2,2,1,1,1,0,0,1,1,2,1,0

1,0,1,1,1,3,1,0,0,1,6,0,1,3,1,1,0

1,0,3,1,1,1,1,1,0,0,0,0,2,1,1,1,0

1,1,0,1,2,3,1,1,0,1,6,0,2,1,1,0,1

1,1,2,1,2,3,1,1,0,1,0,0,2,1,1,0,1

1,1,2,1,2,3,2,1,0,1,2,0,1,1,1,1,1

1,1,1,1,2,3,1,1,1,1,5,0,5,1,1,0,1

1,1,1,1,2,3,1,1,0,1,1,0,2,1,1,0,1

1,1,1,1,2,3,1,1,1,1,0,0,2,1,1,0,0

1,0,3,1,1,1,1,1,0,1,0,0,5,2,1,1,1

1,0,1,1,2,1,1,1,0,1,0,0,2,1,1,1,0

1,0,3,1,2,1,1,1,0,1,0,0,2,1,1,1,0

1,0,1,1,2,1,1,1,0,1,0,0,2,1,1,1,0

1,1,1,1,3,2,1,1,1,1,6,0,2,1,1,1,1

1,1,3,1,1,2,2,1,1,1,4,0,2,1,1,0,0

1,0,2,1,2,1,1,0,0,1,0,0,2,2,1,1,0

1,1,3,1,2,3,1,1,0,1,0,0,2,1,1,1,0

1,0,0,1,1,2,1,1,0,1,0,0,2,1,1,1,0

1,0,1,1,1,1,1,1,0,1,2,0,2,1,1,1,0

1,0,1,1,2,1,1,1,0,1,2,0,2,1,1,1,0

1,1,1,1,2,1,1,1,1,1,0,0,2,1,1,0,0

7,0,1,1,2,1,1,1,0,1,0,0,2,1,1,1,0

1,0,1,1,2,2,2,1,1,0,0,0,1,1,1,1,0

1,1,1,1,2,3,1,1,0,1,6,0,2,1,1,0,1

1,1,1,1,2,1,2,1,1,1,0,0,1,1,1,1,0

1,0,1,1,2,1,1,1,0,1,0,0,2,1,1,1,0

1,0,1,1,2,1,2,1,1,1,0,0,1,1,1,1,0

1,1,1,1,2,3,1,1,1,1,0,0,2,1,1,0,0

1,0,1,1,2,3,1,1,0,1,1,0,2,1,1,1,1

2,0,1,1,2,1,1,1,1,1,0,0,2,1,1,1,0

1,0,0,1,3,3,1,1,0,1,6,0,2,1,1,1,0

1,0,1,1,1,2,1,1,0,1,0,0,2,1,1,1,1

1,0,1,2,3,1,1,1,0,1,0,0,2,1,1,0,1

1,0,3,1,2,1,1,1,1,1,2,0,2,1,1,1,0

1,1,1,1,1,1,1,2,1,2,1,0,2,1,1,1,0

1,0,3,1,2,2,1,1,0,1,2,0,2,1,1,0,0

1,1,1,1,2,2,1,1,0,1,0,0,2,1,1,1,1

1,1,1,1,2,1,2,1,1,1,0,0,2,1,1,1,1

1,0,1,1,2,1,1,1,0,1,2,0,2,1,1,1,1

1,1,3,1,1,1,1,1,1,1,0,0,2,1,1,0,0

1,0,1,1,2,1,2,1,1,1,2,0,2,1,1,0,1

1,1,1,1,1,1,2,1,1,1,6,0,2,1,1,1,1

1,0,1,1,2,2,1,1,1,1,0,0,2,1,1,0,1

1,1,1,1,2,1,2,1,0,1,0,0,1,1,1,1,1

1,1,1,1,2,3,1,0,0,1,0,0,2,1,1,1,0

1,0,1,1,1,3,1,0,0,1,2,0,2,3,1,1,0

1,0,0,1,2,3,1,1,0,1,6,0,2,2,1,1,0

1,0,1,1,2,1,1,0,0,1,1,0,2,2,1,1,0

1,0,1,1,2,2,1,1,0,1,0,0,2,1,1,1,1

1,0,3,1,2,1,1,1,0,1,0,0,2,1,1,1,1

1,1,3,1,1,1,1,1,0,1,0,0,2,1,1,0,1

1,1,1,1,2,1,1,1,0,1,0,0,2,2,1,1,0

1,0,2,1,3,1,1,1,1,1,2,0,1,1,1,0,0

1,1,1,1,2,1,2,1,1,1,2,0,2,1,1,0,1

1,1,3,1,2,2,2,1,1,1,0,0,2,1,1,1,0

1,0,1,1,2,1,2,1,0,1,0,0,1,1,1,1,1

1,0,1,1,2,2,1,1,1,1,5,0,2,1,1,0,1

1,1,1,1,2,3,1,1,0,1,1,0,2,1,1,0,1

1,0,3,1,2,2,1,1,1,1,2,0,2,1,1,1,0

1,0,0,1,2,1,1,1,0,1,2,0,2,1,13,0,0

1,0,1,1,2,1,1,1,0,1,0,0,2,1,1,1,0

1,0,1,1,2,1,1,1,0,1,0,0,2,2,1,1,0

1,0,1,1,2,3,1,1,0,1,0,0,2,1,1,1,0

1,0,1,1,2,1,1,1,1,1,0,0,2,2,1,1,0

1,0,3,1,2,2,1,1,0,1,0,0,2,1,1,1,0

1,0,1,1,2,2,2,1,0,1,0,0,2,1,1,1,1

1,1,3,1,2,1,1,1,0,1,0,0,2,1,1,0,1

1,1,1,1,2,1,1,1,0,1,1,0,2,1,1,0,1

1,0,2,1,2,4,2,1,1,1,0,0,2,1,1,1,0

1,1,1,1,2,3,1,1,1,1,0,0,2,1,1,0,1

1,0,1,1,1,3,1,0,0,1,2,0,2,3,1,1,0

1,1,1,1,2,1,1,1,0,1,4,0,2,1,1,1,0

1,1,1,1,1,3,1,1,0,1,2,0,2,1,1,1,0

1,0,1,1,2,1,2,0,0,1,0,0,1,1,1,1,1

1,0,3,1,2,1,1,1,0,1,1,0,2,1,1,1,1

1,0,0,1,2,2,1,1,0,1,0,0,2,1,1,1,0

1,0,1,1,2,3,1,1,1,1,6,0,2,1,1,1,0

1,0,1,1,2,3,1,1,1,1,6,0,2,1,1,1,1

1,0,3,1,2,3,1,1,0,1,0,0,2,1,1,1,0

1,0,1,1,2,4,1,1,0,1,0,0,2,3,1,1,0

1,0,1,1,1,1,1,1,0,1,0,0,2,1,1,1,0

1,1,2,1,2,1,2,1,1,1,0,0,1,1,1,1,1

1,1,1,1,2,1,1,1,1,1,0,0,2,1,1,1,1

1,0,1,1,2,2,1,1,1,1,1,0,2,1,1,1,0

1,0,1,1,2,1,1,1,1,1,2,0,2,1,1,1,0

1,0,1,1,3,1,2,1,0,1,1,0,1,1,1,1,1

7,0,3,1,2,1,2,1,0,1,0,0,2,1,1,1,0

1,1,0,1,2,3,1,1,0,1,6,0,2,1,1,1,0

1,0,1,1,2,1,1,1,0,0,2,0,2,1,1,0,1

1,0,1,1,2,1,1,1,1,1,2,0,2,1,1,1,0

1,0,1,1,1,1,1,1,1,1,1,0,2,1,1,0,0

1,0,1,1,1,1,1,1,1,1,1,0,2,1,1,1,1

1,1,3,1,2,1,2,1,1,1,0,0,1,1,1,1,1

1,1,1,1,2,3,1,1,0,1,1,0,2,1,1,0,1

1,0,3,1,2,1,1,1,1,0,2,0,2,1,1,0,1

1,0,1,1,2,2,1,1,0,1,2,0,2,1,1,0,0

1,0,0,1,2,3,2,1,0,1,6,0,1,1,1,1,1

1,1,1,1,2,2,1,1,1,0,0,0,2,1,1,0,0

1,0,1,1,1,3,1,1,0,1,6,0,2,1,1,1,0

1,0,0,1,2,2,1,1,1,1,0,0,1,1,1,1,0

1,0,0,1,2,3,2,1,1,1,2,0,2,1,1,0,1

1,0,3,1,1,1,1,1,0,1,0,0,2,1,1,1,1

2,1,3,1,1,4,1,1,0,1,0,0,2,1,1,1,1

1,1,1,1,2,3,1,1,1,1,0,0,2,1,1,0,1

1,0,2,1,2,1,1,1,0,1,2,0,2,1,1,1,1

1,1,1,1,2,3,1,1,0,1,0,0,2,1,1,0,1

1,1,1,1,2,1,1,1,1,1,2,0,2,1,1,1,0

1,1,3,1,2,1,1,1,0,1,2,0,2,1,1,1,0

1,0,1,1,2,2,2,1,1,1,0,0,1,1,1,1,0

1,0,2,1,2,2,2,1,0,1,0,0,1,1,1,1,0

12,0,1,1,2,1,1,1,1,1,0,0,1,1,1,1,0

3,0,1,1,2,2,1,1,0,1,0,0,1,1,1,1,0

1,1,1,1,2,3,1,1,0,1,1,0,2,1,1,1,0

1,1,1,1,1,4,1,1,0,1,4,0,2,1,1,1,1

1,0,1,1,2,1,1,1,1,1,2,0,2,1,1,1,1

1,0,1,1,2,1,2,1,1,0,2,0,1,1,1,1,0

1,0,1,1,2,4,1,1,0,1,1,0,2,1,1,1,1

1,0,1,1,2,1,1,1,0,1,0,0,2,1,1,1,1

1,0,1,1,2,1,1,1,1,1,3,0,2,1,1,0,1

1,0,1,1,2,3,1,1,1,1,0,0,2,1,1,0,1

1,0,1,1,2,3,1,1,0,1,0,0,2,1,1,1,0

1,0,1,1,1,2,1,1,0,1,0,0,2,1,1,1,0

1,1,0,1,2,3,1,1,1,1,6,0,2,1,1,0,1

1,0,1,1,2,1,1,1,0,1,0,0,2,1,1,1,0

1,0,1,1,2,1,1,1,1,1,5,0,2,1,1,1,0

1,0,1,1,2,1,1,0,1,0,0,0,2,1,1,0,1

1,1,1,1,2,1,2,1,0,1,6,0,2,2,1,1,0

1,0,1,1,2,1,1,1,0,1,0,0,2,1,1,1,1

1,0,3,1,2,3,1,1,0,1,4,0,2,1,1,1,0

1,1,0,1,2,3,2,1,1,1,6,0,2,1,1,0,0

1,0,1,1,2,1,1,1,0,1,4,0,2,1,1,1,1

1,0,1,1,2,1,1,1,0,1,1,0,1,1,1,1,0

12,0,3,1,2,2,1,0,0,1,2,0,2,2,1,1,0

1,0,1,1,2,2,1,1,0,1,6,0,2,1,1,1,1

1,0,1,1,2,3,1,1,0,1,4,0,2,1,1,1,1

1,0,2,1,2,1,1,1,1,1,2,0,2,1,1,1,0

1,0,1,1,2,1,1,1,1,1,0,0,1,1,1,1,0

1,0,2,1,3,1,2,1,1,1,0,0,1,1,1,1,0

1,0,1,1,2,1,2,1,1,1,0,0,1,1,1,1,0

1,1,1,1,1,1,1,1,0,1,0,0,2,1,1,1,0

1,0,3,1,2,1,2,1,1,1,0,0,2,1,1,0,1

1,0,1,1,1,2,2,1,1,1,6,0,1,1,1,1,0

1,0,1,1,2,3,2,1,1,1,2,0,2,1,1,1,0

1,0,1,1,2,2,1,1,1,1,0,0,2,1,1,1,0

1,0,1,1,2,1,1,1,0,0,2,0,2,1,1,1,1

1,0,1,1,2,1,1,0,0,1,0,0,2,2,1,1,0

1,0,1,1,2,2,1,1,1,1,0,0,2,1,1,1,0

1,0,2,1,2,2,2,2,1,0,4,0,1,1,1,1,0

1,0,1,1,3,1,1,1,1,1,2,0,1,1,1,1,0

1,1,1,1,1,1,1,1,0,1,1,1,2,1,1,0,1

1,0,2,1,2,1,1,1,0,1,2,0,2,1,1,1,1

8,0,1,2,3,2,1,1,0,1,0,0,2,1,1,0,0

1,0,1,1,1,3,1,1,1,1,6,0,2,1,1,1,0

1,0,1,1,2,1,1,1,1,1,0,0,2,1,1,0,1

1,0,1,1,2,2,1,1,1,1,1,0,2,1,1,1,0

1,0,1,1,1,1,2,1,1,1,0,0,1,1,1,0,0

1,1,3,1,1,1,1,2,1,2,1,0,2,1,1,0,0

1,1,2,1,2,3,1,1,0,1,2,0,2,1,1,0,1

1,1,0,1,2,3,1,1,1,1,6,0,2,1,1,0,0

1,0,1,1,2,1,1,1,0,1,2,0,2,1,1,1,0

1,0,0,1,2,3,2,1,1,1,2,0,2,1,1,1,0

1,1,1,1,1,3,2,1,0,1,6,0,1,1,1,1,0

1,0,2,1,2,2,1,1,1,1,2,0,2,1,1,0,1

1,0,1,1,1,2,1,1,1,1,0,0,2,1,1,0,0

1,0,1,1,2,1,1,1,0,1,1,0,2,1,1,0,1

1,1,3,1,2,1,1,1,0,1,0,0,2,1,1,1,0

1,0,3,1,2,2,1,1,0,1,1,0,2,1,1,1,0

1,0,3,1,2,1,1,1,1,1,0,0,1,1,1,1,0

1,1,1,1,2,3,1,1,1,1,4,0,2,1,1,1,1

1,1,1,1,1,1,1,1,0,1,0,0,2,1,1,1,0

1,0,1,1,1,2,1,1,0,1,0,0,2,1,1,0,0

1,0,0,1,2,3,1,1,0,1,6,0,2,1,1,1,0

1,0,1,1,2,1,2,1,1,1,2,0,1,1,1,1,1

1,1,0,1,2,3,1,1,0,1,6,0,2,1,1,1,0

1,0,1,1,2,2,1,1,1,1,1,0,2,1,1,0,0

1,1,1,1,2,3,1,1,0,1,4,0,2,1,1,1,0

1,0,1,1,2,1,1,1,0,1,6,0,2,1,13,1,0

1,0,1,1,2,1,1,1,0,1,0,0,2,1,1,0,0

1,1,1,1,2,3,1,1,0,1,2,0,2,1,1,0,1

1,1,2,1,2,3,1,1,0,1,0,0,2,1,1,1,1

1,0,1,1,2,3,1,1,0,1,0,0,2,1,1,1,1

1,0,1,1,2,2,1,1,1,1,1,0,2,1,1,1,0

1,0,1,1,2,1,1,0,0,0,2,0,2,1,1,1,1

1,1,0,1,2,3,2,1,1,1,2,0,2,1,1,0,0

1,1,3,1,2,1,1,1,1,1,0,0,2,1,1,0,0

1,0,1,1,2,1,1,1,0,1,0,0,2,1,1,1,0

1,0,1,1,2,1,1,1,0,1,1,0,2,1,1,1,0

1,0,1,1,2,1,1,1,0,1,0,0,2,2,1,1,0

1,0,1,1,2,2,1,1,0,1,0,0,2,1,1,1,0

1,0,1,1,2,1,2,1,1,1,0,0,1,1,1,1,0

1,0,1,1,2,2,1,1,0,1,0,0,2,1,1,1,0

1,0,2,1,2,1,1,1,0,1,1,0,2,1,1,1,0

1,0,3,1,2,1,1,1,0,1,2,0,2,1,1,1,0

1,1,0,1,2,3,2,1,1,1,2,0,2,2,1,1,0

1,1,2,1,2,2,2,1,0,1,0,0,2,1,1,1,1

1,1,2,1,2,2,2,1,1,1,0,0,1,1,1,1,0

1,0,1,1,2,1,1,1,1,1,0,0,1,1,1,1,1

1,1,1,1,2,3,1,1,0,1,0,0,2,1,1,0,0

1,0,1,1,2,1,1,1,0,1,0,0,2,1,1,1,0

1,0,1,1,2,3,1,1,1,1,2,0,2,1,1,0,1

1,1,1,1,2,3,2,1,1,1,2,0,2,1,1,1,0

1,0,3,1,2,1,2,1,1,1,0,0,1,1,1,1,0

1,0,1,1,2,1,1,1,0,1,6,0,2,1,1,1,0

1,0,1,1,2,3,1,1,0,1,0,0,2,1,1,1,0

1,1,1,1,2,3,1,1,1,1,0,0,2,1,1,0,0

1,1,1,1,2,1,1,1,0,1,0,0,2,2,1,0,1

7,0,1,2,3,1,1,1,0,1,6,0,2,1,1,0,0

1,1,1,1,1,4,1,1,0,1,0,0,2,3,1,1,0

1,0,1,1,2,2,1,1,0,1,0,0,2,2,1,1,0

1,1,1,1,2,1,2,1,1,1,1,0,2,1,1,0,0

1,0,2,1,3,2,2,1,1,1,1,0,1,1,1,0,0

1,1,1,1,2,3,2,1,1,0,0,0,1,2,1,1,1

1,1,1,1,2,3,1,1,1,1,0,0,2,1,1,1,1

1,0,1,1,2,1,1,1,0,1,0,0,2,1,1,1,1

1,0,1,1,1,1,1,1,0,1,0,0,2,1,1,1,0

1,1,1,1,1,1,1,1,0,1,5,0,1,2,1,0,0

1,1,1,1,2,1,1,1,0,1,0,0,2,1,1,0,1

1,0,1,1,2,1,2,1,1,0,0,0,1,1,1,1,0

1,0,1,1,2,1,2,1,0,1,1,0,2,1,1,1,1

1,0,1,1,3,1,1,1,0,1,0,0,2,1,1,1,0

1,1,3,1,1,2,1,1,0,1,0,0,2,1,1,1,0

1,0,1,1,2,3,1,1,0,1,2,0,2,1,1,1,0

1,0,1,1,1,2,1,1,0,1,0,0,2,1,1,1,0

1,0,1,1,3,1,2,1,1,1,0,0,2,1,1,1,0

1,0,1,1,3,1,2,0,0,1,0,1,2,1,2,0,0

1,0,1,1,2,1,1,1,1,1,0,0,2,1,1,1,0

1,0,2,1,2,1,2,1,1,1,2,0,2,1,1,0,1

1,0,1,1,2,2,1,1,0,1,4,0,2,1,1,1,0

1,0,3,1,3,1,1,1,0,1,0,0,2,1,1,1,0

1,0,1,1,2,1,2,1,1,1,0,0,1,1,1,1,0

1,0,3,1,2,3,1,1,0,1,0,0,2,1,13,0,1

1,1,0,1,2,3,2,1,1,0,6,0,2,1,1,0,0

1,1,0,1,2,1,1,1,1,1,0,0,2,1,1,0,1

1,1,0,1,2,1,1,1,0,1,0,0,2,1,1,0,1

1,1,3,1,2,1,1,1,0,1,0,0,2,1,1,0,1

1,1,1,1,2,1,1,1,1,1,0,0,2,1,1,0,0

1,0,1,1,2,1,1,1,0,1,1,0,2,1,2,0,0

1,1,1,1,2,1,1,1,0,1,0,0,5,2,1,1,0

1,1,0,1,2,3,1,1,0,1,6,0,2,1,1,0,1

1,0,1,1,2,1,1,1,0,1,0,0,2,1,1,1,0

1,0,1,1,1,2,2,1,0,0,1,0,1,1,1,1,0

12,1,1,1,3,3,1,1,0,1,0,0,2,1,1,1,1

1,0,0,1,2,3,1,1,0,1,6,0,1,1,1,1,0

1,0,1,1,1,1,1,0,0,1,0,0,2,1,1,1,0

1,0,1,1,2,1,1,1,0,1,0,0,1,1,1,1,0

1,0,1,1,2,1,1,1,0,1,0,0,2,1,1,1,0

1,0,1,1,2,1,2,1,0,1,0,0,1,1,1,1,0

1,0,1,1,3,1,2,1,1,1,0,0,2,1,1,1,1

1,1,2,1,2,1,1,1,1,0,1,0,1,1,1,1,0

1,0,1,1,2,1,1,1,1,1,2,0,2,1,1,1,0

1,0,1,1,2,1,1,1,0,1,0,0,2,1,1,1,1

1,1,0,1,2,3,2,1,0,0,6,0,1,1,1,1,0

1,0,3,1,2,1,1,1,0,0,2,0,2,1,1,1,1

1,0,2,1,2,1,1,1,0,1,2,0,2,1,1,1,1

1,0,3,1,2,1,1,1,0,1,0,0,2,1,1,1,0

1,1,1,1,2,3,1,1,0,1,0,0,2,1,1,0,1

1,1,1,1,1,2,2,1,1,1,0,0,2,1,1,1,0

1,0,1,1,2,1,2,1,0,1,2,0,1,1,1,1,0

1,1,1,1,1,1,2,1,1,1,0,0,2,1,1,1,0

1,1,1,1,2,2,2,1,1,1,2,0,1,1,1,1,0

1,0,2,1,2,2,2,1,0,1,2,0,2,1,1,0,1

1,1,3,1,2,1,1,1,0,1,1,0,2,1,1,0,1

1,1,1,1,2,2,2,1,1,1,2,0,2,1,1,1,1

1,0,1,1,2,1,1,1,0,1,0,0,2,1,1,1,0

1,1,1,1,2,1,1,1,1,1,0,0,2,1,1,0,1

1,0,1,1,3,1,1,1,0,1,1,0,2,1,1,1,0

1,1,3,1,2,1,1,1,0,1,0,0,1,1,1,1,1

1,0,3,1,2,3,2,0,0,0,0,0,1,1,13,0,1

1,1,3,1,2,1,1,1,0,0,0,0,2,1,1,0,1

1,0,1,1,2,1,1,1,0,1,0,0,2,1,1,1,0

1,0,1,1,3,2,2,1,0,1,0,0,1,1,2,1,0

1,0,1,1,2,3,1,1,0,1,0,0,2,1,1,1,1

1,0,1,1,2,1,1,1,0,1,0,0,2,1,1,1,1

1,0,1,1,2,1,1,1,1,1,0,0,1,1,1,1,0

1,0,1,1,2,1,1,1,1,1,2,0,2,1,1,0,0

1,0,3,1,2,1,2,0,1,1,2,0,2,1,1,1,0

1,0,1,1,2,3,1,1,0,1,0,0,2,1,1,1,1

1,0,1,1,2,1,1,0,0,0,0,0,2,1,1,1,1

1,1,2,1,2,3,1,1,1,1,0,0,2,1,1,1,0

1,0,1,1,2,1,1,1,1,1,2,0,2,1,1,0,1

1,1,1,1,1,3,1,0,0,1,6,0,1,1,1,1,0

1,0,1,1,1,2,1,1,1,1,0,0,2,1,1,1,0

1,1,1,1,2,2,2,1,0,0,0,0,1,1,1,0,0

1,0,1,1,2,1,1,0,0,1,0,0,2,3,1,1,0

1,1,1,1,2,3,1,1,1,1,2,0,2,1,1,0,1

1,0,1,1,2,1,1,1,0,1,0,0,2,1,1,1,1

1,0,1,1,2,2,2,1,1,1,0,0,1,1,1,1,0

1,1,3,1,1,3,1,1,0,1,6,0,2,2,1,1,0

1,1,0,1,2,3,2,1,1,1,2,0,2,1,1,1,0

1,1,1,1,2,3,1,1,0,1,1,0,2,1,1,0,1

1,1,1,1,2,1,1,2,1,2,1,0,1,1,1,1,0

1,1,1,1,2,1,1,1,0,1,0,0,2,1,1,1,0

1,1,0,1,2,3,1,1,1,1,2,0,2,1,1,1,1

1,1,3,1,1,3,1,1,0,1,6,0,2,1,1,1,0

1,1,1,1,3,2,1,1,0,1,6,0,2,1,1,1,1

1,0,1,1,1,3,1,1,0,1,0,0,2,1,1,1,1

1,0,3,1,1,2,1,1,0,1,0,0,2,2,1,1,1

1,0,1,1,2,1,2,1,1,1,2,0,1,1,1,1,0

1,0,1,1,2,1,1,1,0,1,0,0,2,1,1,1,0

1,0,1,1,2,2,1,1,1,1,1,0,2,1,1,0,0

1,1,2,1,2,3,1,1,0,1,1,0,2,1,1,0,1

1,0,3,1,2,2,1,1,0,1,1,0,2,1,1,1,1

1,1,0,1,2,2,2,1,0,1,0,0,1,1,1,1,0

1,0,1,1,2,3,1,1,0,1,2,0,2,1,1,1,0

1,0,1,2,2,2,1,1,0,1,0,0,2,2,1,0,1

1,0,1,1,2,3,1,1,0,1,3,0,2,1,1,0,1

1,1,1,1,2,2,1,1,0,1,0,0,2,1,1,1,1

1,1,1,1,2,3,1,1,0,1,1,0,2,1,1,1,1

1,0,1,1,2,1,1,1,0,1,0,0,2,1,1,1,1

1,0,0,1,2,1,1,1,0,1,1,0,2,1,1,1,1

1,0,1,1,1,1,2,1,1,1,2,0,2,1,1,1,0

1,1,2,1,2,3,1,1,1,1,6,0,2,1,1,0,1

1,0,1,1,2,1,2,1,1,1,0,0,1,1,1,1,0

1,0,0,1,2,1,2,1,1,0,0,0,1,1,1,1,0

1,0,1,1,2,1,1,0,0,0,0,0,2,1,1,1,1

5,0,1,2,3,1,2,1,0,1,0,0,2,1,1,1,0

1,0,1,1,3,1,1,1,0,1,0,0,2,1,1,1,0

1,1,1,1,2,3,2,0,0,1,2,0,1,1,1,1,0

1,1,3,1,2,2,1,1,1,1,0,0,2,1,1,0,0

1,1,0,1,2,3,2,1,1,1,2,0,2,1,1,1,0

1,1,0,1,2,3,2,1,1,1,6,0,2,1,1,0,0

1,0,1,1,2,1,1,1,0,1,2,0,1,1,1,1,1

1,0,0,1,3,3,1,1,1,1,2,0,2,1,1,1,0

1,0,0,1,2,3,1,1,0,1,6,0,2,1,1,1,0

1,0,3,1,2,1,1,1,0,1,6,0,2,1,13,0,0

1,0,2,1,2,2,2,1,1,1,0,0,2,1,1,1,0

1,1,1,1,2,3,1,1,0,1,0,0,2,1,1,1,1

1,1,1,1,2,2,1,1,1,1,0,0,2,1,1,0,0

1,0,3,1,2,3,1,1,0,0,0,0,2,1,1,1,1

1,0,1,1,2,1,2,1,0,1,0,0,1,1,1,1,0

1,1,1,1,3,3,1,1,0,1,3,0,2,1,1,1,1

1,1,1,1,2,3,1,1,1,1,0,0,2,1,1,0,0

1,0,1,1,2,1,1,1,0,1,0,0,2,1,1,1,1

1,0,1,1,2,2,2,1,1,1,2,0,1,1,1,1,0

1,1,0,1,2,3,2,1,1,1,2,0,2,1,1,1,0

1,0,3,1,1,2,1,1,0,1,0,0,2,1,1,1,0

1,1,1,1,2,3,1,1,0,1,0,0,2,1,1,0,1

1,1,1,1,2,3,1,1,1,1,2,0,2,1,1,0,0

1,0,1,1,1,3,1,1,0,1,2,0,2,2,1,1,0

1,0,1,1,4,3,2,1,0,1,6,0,2,1,1,1,1

1,1,1,1,1,1,1,1,0,1,0,0,2,1,1,0,1

1,0,2,1,2,1,2,1,1,1,2,0,1,1,1,1,1

1,1,0,1,2,3,2,1,1,1,6,0,2,1,1,0,0

1,1,1,1,2,3,1,1,0,1,4,0,2,1,1,0,1

1,0,1,1,2,1,1,1,0,1,1,0,2,1,1,1,1

1,0,1,1,2,1,1,1,0,1,0,0,2,1,1,1,0

1,0,1,1,2,1,1,1,0,1,0,0,2,1,1,1,1

1,0,1,1,2,1,1,1,1,1,4,0,2,1,1,1,0

1,0,1,1,2,1,1,1,0,1,1,0,2,1,1,1,1

1,0,1,1,2,1,1,1,0,1,4,0,2,1,1,1,1

1,0,1,1,2,1,1,1,0,1,0,0,2,1,1,1,0

1,1,3,1,2,2,2,1,1,1,4,0,1,1,1,1,1

1,0,1,1,2,2,1,1,0,1,0,0,2,1,1,1,1

1,1,0,1,2,3,1,1,1,1,2,0,2,1,1,0,0

1,1,0,1,2,2,2,1,1,1,0,0,2,1,1,0,1

1,1,1,1,2,3,1,1,0,1,5,0,2,1,1,0,1

1,1,1,1,2,2,2,1,1,1,2,0,1,1,1,1,1

1,1,2,1,2,1,2,1,1,1,0,0,2,1,1,0,1

1,1,3,1,2,2,1,1,1,1,0,0,2,1,1,0,1

1,0,1,1,2,2,1,1,0,1,0,0,2,1,1,1,1

1,1,1,1,2,3,1,1,0,0,0,0,2,1,1,1,0

1,1,1,1,2,1,2,1,1,1,0,0,2,1,1,0,0
