# Supplementary material for: Comparison of three data mining models for prediction of advanced schistosomiasis prognosis in the Hubei province
Source: PLoS Negl Trop Dis. 2018 Feb 15;12(2):e0006262. doi: 10.1371/journal.pntd.0006262 (PMC5831639; doi:10.1371/journal.pntd.0006262)
Supplement: S2 Dataset — (ZIP) [file pntd.0006262.s004.zip › The weights of viariables in each model/DT model.docx]

"","name","weight"

"1"," Clinical classification ", 109.309809914972

"2"," History of splenectomy ", 104.31479917702

"3"," Cost of treatment ", 95.0786426798636

"4"," Annual Income ", 75.9096001761074

"5"," Viability ", 40.7741898555553

"6","Diagnostic Evidence2", 27.8572985284591

"7"," Means of treatment", 19.1609489460788

"8"," Diagnostic Evidence1",15.9480948002107

"9"," Other disease ",7.08344742891606

"10"," Occupation ",7.0753456868635

"11","BMI",4.80273969305027

"12"," History of ascites",3.03022780097133

"13"," Type of treating patients ",0.726660701062534

"14"," Prior treatment ",0.151854589857585
