# Supplementary material for: Comparison of three data mining models for prediction of advanced schistosomiasis prognosis in the Hubei province
Source: PLoS Negl Trop Dis. 2018 Feb 15;12(2):e0006262. doi: 10.1371/journal.pntd.0006262 (PMC5831639; doi:10.1371/journal.pntd.0006262)
Supplement: S2 Dataset — (ZIP) [file pntd.0006262.s004.zip › The weights of viariables in each model/LR model.docx]

"","name","coefficient"

"1","Intercept",7.99705615666506

"2"," Occupation ",0.136075933902856

"3"," Annual Income ",-1.3738325905187

"4","BMI",-0.00576153988096889

"5"," Viability ",-2.92138347557794

"6"," Nourishment ",-0.225348324773839

"7","Diagnostic Evidence1",0.106460210159237

"8"," Diagnostic Evidence2",0.536998255872574

"9"," Prior treatment ",-0.150641189766785

"10"," History of splenectomy ",-1.17511179658512

"11"," History of ascites ",0.0399952140900452

"12"," Other disease ",-0.0401185449125897

"13"," The extent of ascites ",-17.7140507735886

"14","Clinical classification",-1.4549747872333

"15"," Type of treating patients ",0.224817772041969

"16"," Means of treatment ",-0.198686180690758

"17"," Cost of treatment ",-1.28632608682793
